# Hematological toxicity of parp inhibitors in solid tumors: a systematic review and safety meta-analysis

**DOI:** 10.1007/s10555-025-10283-1

**Published:** 2025-08-14

**Authors:** Brigida Anna Maiorano, Martina Catalano, Mauro Francesco Pio Maiorano, Alessio Signori, Vera Loizzi, Gennaro Cormio, Michele Reni, Giandomenico Roviello, Andrea Necchi

**Affiliations:** 1 Department of Medical Oncology, IRCCS San Raffaele Hospital, Milan, Italy; 2 Department of Health Sciences, Section of Clinical Pharmacology and Oncology, University of Florence, Florence, Italy; 3 Gynecologic Oncology Unit, IRCCS Istituto Tumori “Giovanni Paolo II”, Bari, Italy; Department of Interdisciplinary Medicine (DIM), University of Bari, Bari, Italy; 4 Department of Health Sciences, University of Genoa, Genoa, Italy; 5 Vita-Salute San Raffaele University, Milan, Italy

**Keywords:** PARP inhibitors, Hematological toxicity, Solid tumors, Anemia, Neutropenia, Thrombocytopenia, MCRPC, Ovarian cancer, Breast cancer, Prostate cancer

## Abstract

Poly (ADP-ribose) polymerase (PARP) inhibitors (PARPis) are effective agents in different tumor types. A typical class of adverse events (AEs) associated with these agents, often leading to treatment modifications and discontinuations of treatment, is hematological toxicity. In our systematic review and safety meta-analysis, we investigated the incidence and risk of all grades and ≥ grade (G) 3 hematological AEs, including anemia, neutropenia, thrombocytopenia, and acute myeloid leukemia (AML)/myelodysplastic syndrome (MDS) due to PARPis, used alone or in combination, in patients diagnosed with solid tumors. This systematic review and meta-analysis followed the Preferred Reporting Items for Systematic Reviews and Meta-Analysis (PRISMA) statement. We systematically searched the PubMed, EMBASE, and Cochrane databases, the American Society of Clinical Oncology, and the European Society of Medical Oncology meeting abstracts for randomized clinical trials (RCTs) concerning the use of PARPis in patients with solid tumors. The search deadline was April 30, 2024. We calculated risk ratios (RRs) for all-grade and ≥ G3 AEs of PARPis versus non-PARPis. 31 phase II/III RCTs were included. Anemia was the most common all-grade (49.2%) and ≥ G3 AE (25.0%). The administration of PARPis significantly increased the risk of developing all grades of anemia (RR = 2.15, p < 0.00001), neutropenia (RR = 1.50, p = 0.0002), and thrombocytopenia (RR = 2.59, p < 0.00001) compared to non-PARPis. Similarly, a significant increase in the risk of ≥ G3 anemia (RR = 5.43, p < 0.00001), neutropenia (RR = 1.70, p = 0.002), and thrombocytopenia (RR = 5.42, p < 0.00001) was detected. PARPis did not increase the risk of AML/MDS (p = 0.86). PARPis increase the risk of hematologic toxicity compared to other treatments in solid tumors. Clinicians should be aware of this risk, especially given the expected increase in PARPis use in the next year in different tumor types.

**Supplementary information:**

The online version contains supplementary material available at 10.1007/s10555-025-10283-1.

## Introduction

Poly (ADP-ribose) polymerase (PARP) inhibitors (PARPis) represent some of the most promising advancements in modern oncology. Several PARPis are now approved for the treatment of various tumor types, including ovarian, breast, pancreatic, and prostate cancer [1]. Ongoing research into additional agents and combinations will expand future treatment options for various tumor types.

The primary recognized role of PARPis pertains to tumors carrying germinal or somatic mutations in the BReast CAncer (BRCA)1/2 genes, which play a crucial role in repairing DNA double-strand breaks (DSB) through homologous recombination (HR). In cases where BRCA1/2 is defective, PARP-1 and −2 become particularly valuable in identifying single-strand breaks (SSB) and repairing them through the base excision repair (BER) mechanism. Therefore, in the presence of BRCA and other HR gene mutations, the inhibition of PARP results in the cell's inability to repair DNA, leading to the accumulation of DSBs and, ultimately, cell death [2].

While PARPis undeniably exhibit efficacy, they are associated with certain characteristic adverse events (AEs). Hematological toxicities, such as anemia, thrombocytopenia, and neutropenia, are some of the most frequently reported AEs in randomized clinical trials (RCTs) involving PARPi treatments [3]. These AEs can necessitate treatment interruption, discontinuation, and dose reduction, making it essential for clinicians to comprehensively understand their management in the daily clinical practice of various tumor types.

In our study, we conducted an updated safety meta-analysis of phase II/III RCTs involving PARPis to investigate the incidence and relative risk of hematological AEs, encompassing both all grades and those exceeding grade 3 (≥ G3), in patients with solid tumors treated with PARPis.

## Methods

### Study selection

Our systematic review and meta-analysis followed the Preferred Reporting Items for Systematic Reviews and Meta-Analyses (PRISMA) guidelines [4]. We registered the full systematic review protocol at PROSPERO (CRD42024564426). A literature search with no data restriction using MEDLINE/PubMed, the Cochrane Library, and the ASCO/ESMO Meeting abstract was carried out on April 30, 2024. The following search terms were used: “PARP” AND “olaparib OR veliparib OR rucaparib OR niraparib OR talazoparib OR pamiparib” AND “ovarian cancer OR prostate cancer OR breast cancer OR pancreatic cancer OR gastric cancer” AND “randomized controlled trial OR clinical trial” (Supplementary Table 1). A crosscheck reference from review articles was performed for all possible pertinent RCT retrieval. The search criteria were limited to phase II and III RCTs.

### Selection criteria

Three independent reviewers (BAM, MC, MFPM) screened the studies for eligibility based on the following selection criteria. The inclusion criteria were based on the PICO (Population- Intervention-Comparator-Outcomes) framework: (P) participants with solid tumors, (I) treated with PARPis (olaparib, niraparib, rucaparib, talazoparib, or veliparib), (C) compared with placebo (PBO) or treatment different than PARPis, (O) reporting rates of hematological toxicities, including anemia, thrombocytopenia, and neutropenia (Supplementary Table 2). The exclusion criteria included: phase I or non-RCT studies; studies with insufficient data; studies involving animal subjects; sample size per arm < 10 participants. No language restriction was applied.

### Data extraction and risk of bias

The following data were independently collected by three authors (BAM, MC, MFPM) for each study: trial name, first author name, publication year, RTC trial phase, experimental and control drugs, median treatment duration, safety population in the experimental and control groups, number and grade of anemia, neutropenia, and thrombocytopenia, acute myeloid leukemia (AML)/myelodysplastic syndrome (MDS). When accessible, hematological toxicities were documented based on the Common Terminology Criteria for Adverse Events (CTCAE) provided by the National Cancer Institute (NCI)[5]. According to the CTCAE scale from 1 to 5,"all-grade"encompasses grades 1 to 5, while"high-grade"encompasses grades ≥ 3. These grades represent the increasing severity of the adverse event in sequential order. It's important to mention that grade 5 is specifically designated as an event leading to a fatality associated with the adverse event, and in some instances, such cases may not be reported.

The Jadad scale, ranging from 0 to 5, was used to evaluate the risk of bias in the included studies [6].

Three authors (BAM, MC, and GR) independently performed the risk of bias and quality assessments for the included studies. The risk of bias for each study was categorized as either low, with some concerns, or high.

## Data analysis

The risk ratio (RR) of PARPis (experimental group) vs. non-PARPis (control group) was calculated for dichotomous data alongside 95% confidence intervals (CIs). The Mantel–Haenszel method was used. Heterogeneity among the included studies was quantified with the I^2^ statistic, with a cut-off of 50% for high heterogeneity^2^. Random- or fixed-effects models were adopted, depending on the heterogeneity among the studies. Subgroup analyses were conducted to investigate the possible sources of heterogeneity, considering tumor types (ovarian, breast, prostate, gastric, and pancreatic cancer), drug type (olaparib, rucaparib, niraparib, talazoparib, and veliparib), treatment regimen (PARPis monotherapy vs. combination), treatment duration (≤ 12 months vs. > 12 months), control group (PBO, chemotherapy [CHT], androgen receptor-targeted agents [ARTA]). For evaluating AML/MDS, which were extremely rare AEs, Peto Odds Ratio (POR) was used [7]. All the analyses were considered statistically significant when p < 0.05. Funnel plots were used to analyze publication bias. Review Manager Version 5.4 software was used.

## Results

### Study selection

**Fig. 1:**
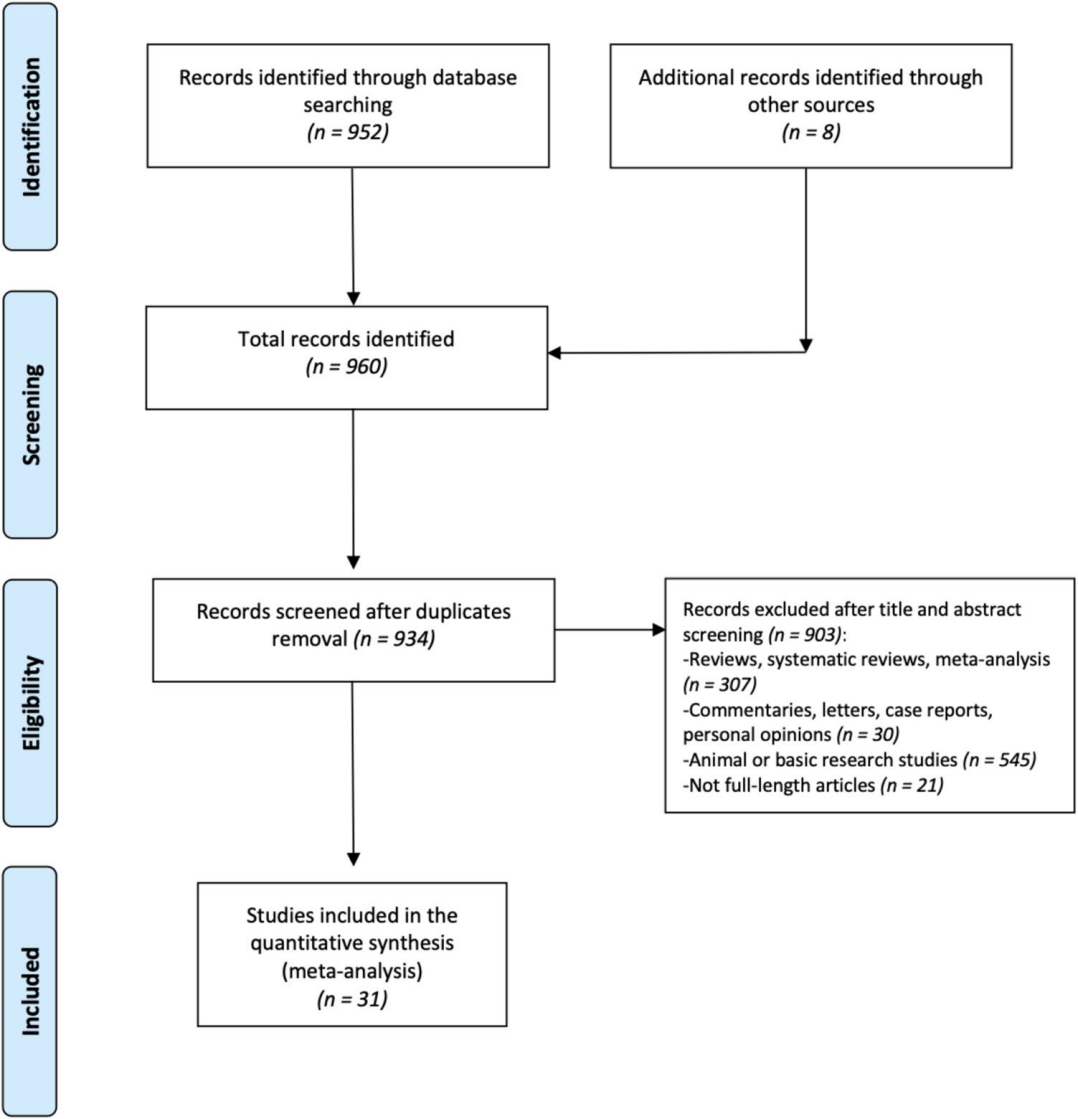
Preferred Reporting Items of Systematic reviews and Meta-Analysis (PRISMA) flow chart of the selection process

The initial search in the literature databases retrieved a total of 952 studies from MEDLINE/PubMed, Cochrane Library, and ASCO/ESMO Meeting abstract, as depicted in Fig. 1 of the PRISMA flowchart. After eliminating 26 duplicate entries and screening titles and abstracts against the inclusion/exclusion criteria, 934 studies were chosen for a thorough examination of their full texts. In total, 31 published phase II and phase III RCTs were incorporated into the systematic review and meta-analysis (Table 1).

**Table 1:** Summary of characteristics of the included randomized clinical trials

Characteristic	Number of Studies
Total studies	31
Phase	
Phase II	10
Phase III	21
Tumor type	
Ovarian cancer	12
Breast cancer	9
Prostate cancer	6
Pancreatic cancer	2
Gastric cancer	2
PARP inhibitor used	
Olaparib	12
Niraparib	5
Rucaparib	4
Talazoparib	2
Veliparib	7
Pamiparib	1
Treatment combination	
Monotherapy	17
With chemotherapy	9
With androgen receptor-targeted agents	4
With bevacizumab	1

### Characteristics of included RTC studies

A total of 31 RCTs were included, of which 10 were phase II and 21 phase III. Study characteristics are summarized in Table 1. In total, 11,708 patients received treatment, with 7,252 in the experimental arm and 4,456 in the control arm. The median age at diagnosis for the included patients ranged from 66 to 70 years. The overall quality of the studies was generally considered good, with Jadad Scores ranging from 3 to 5, and a median score of 4 (Table 2).

**Table 2:** Characteristics of the included studies ARTA: androgen receptor targeted agents; BID: twice daily; CHT: chemotherapy; mCRPC: metastatic castration-resistant prostate cancer; NA: not available; OD: once daily; PBO: placebo

Tumor type	Study	Phase	Line of therapy	Treatment arms	Patients per arm	Treatment duration, months	Jadad score
Ovarian	Study 19	2	2	Olaparib 400 mg BID	136	6.8	5
				PBO	129	4.7	
	SOLO2/ENGOT-Ov21	3	2	Olaparib 300 mg BID	195	19.4	5
				PBO	99	5.6	
	SOLO1/GOG 3004	3	1	Olaparib 300 mg BID	260	24.6	5
				PBO	131	13.9	
	PAOLA-1/ENGOT-ov25	3	1	Olaparib 300 mg BID + bevacizumab	537	17.3	5
				PBO + Bevacizumab	269	15.6	
	SOLO3	3	2	Olaparib 300 mg BID	178	11.3	3
				CHT	88	6	
	CLIO/BGOG-ov10	2	2	Olaparib 300 mg BID	107	NA	3
				CHT	53	NA	
	ENGOT-OV16/NOVA	3	2	Niraparib 300 mg OD	367	NA	5
				PBO	179	NA	
	PRIMA/ENGOT-OV26/GOG-3012	3	1	Niraparib 300 mg—> 200 mg OD	487	NA	5
				PBO	246	NA	
	NORA	3	2	Niraparib 300 mg—> 200 mg OD	177	NA	5
				PBO	88	NA	
	ATHENA-MONO/GOG-3020/ENGOT-ov45	3	1	Rucaparib 600 mg BID	427	14.7	5
				PBO	111	9.9	
	ARIEL3	3	2	Rucaparib 600 mg BID	375	8.3	5
				PBO	189	5.5	
	ARIEL-4	3	2	Rucaparib 600 mg BID	233	7.3	3
				PBO	116	3.6	
Breast	OLYMPIAD	3	≥ 2	Olaparib 300 mg BID	205	8.2	3
				CHT	97	3.4	
	EMBRACA	3	1	Talazoparib 1 mg OD	287	6.9	3
				CHT	144	3.9	
	BROCADE	2	≥ 1	Veliparib 120 mg BID + CHT	97	NA	5
				PBO + CHT	99	NA	
				Veliparib 40 mg BID + CHT	94	NA	
	BROCADE-3	3	≥ 1	Veliparib 120 mg BID + CHT	337	NA	5
				PBO + CHT	172	NA	
	SWOG S1416	2	2	Veliparib 300 mg BID + CHT	320	NA	5
				PBO + CHT	158	NA	
	NCT01351909	2	≥ 2	Veliparib 60 mg ODD + CHT	21	NA	5
				PBO + CHT	18	NA	
	BrighTNess	3	Neo-adjuvant	Veliparib 50 mg BID + CHT	316	NA	5
				PBO + CHT (carboplatin plus paclitaxel)	160		
				PBO + CHT (paclitaxel)	158	NA	
	NCT01042379	2	Neo-adjuvant	Veliparib 50 mg BID + CHT	72	NA	3
				CHT	44	NA	
	BRAVO	3	≥ 2	Niraparib 300 mg OD	141	NA	3
				CHT	74	NA	
Pancreas	PMID:31976786	2	1	VEL 80 mg BID + CHT	27	NA	3
				CHT	23	NA	
	POLO	3	1	Olaparib 300 mg BID	92	6.0	5
				PBO	62	3.7	
Gastric	NCT01063517	2	2	Olaparib 100 mg + CHT	62	11.7	5
				PBO + CHT	62	4.1	
	PARALLAL-303	2	1	Pamiparib 60 mg	71	2.4	5
				PBO	65	1.9	
mCRPC	PROFound	3	2	Olaparib 300 mg BID	162	7.4	3
				ARTA	83	3.9	
				Olaparib 300 mg BID	94	7.4	
				ARTA	48	3.9	
	Triton3	3	2	Rucaparib 600 mg BID	270	8.3	3
				ARTA	135	5.1	
				CHT	75		
	Study 08	2	2	Olaparib 300 mg + Abiraterone	71	10.1	5
				Abiraterone + PBO	71	8.3	
	MAGNITUDE	3	1	Niraparib 200 mg OD + Abiraterone	212	NA	5
				Abiraterone + PBO	211	NA	
	PROpel	3	1	Olaparib 300 mg BID + Abiraterone	399	17.5	5
				Abiraterone + PBO	397	15.7	
	TALAPRO-2	3	1	Talazoparib 0.5 mg OD + enzalutamide	402	19.8	5
				PBO + Enzalutamide	402	16.1	

### All grades hematological toxicity

**Fig. 2:**
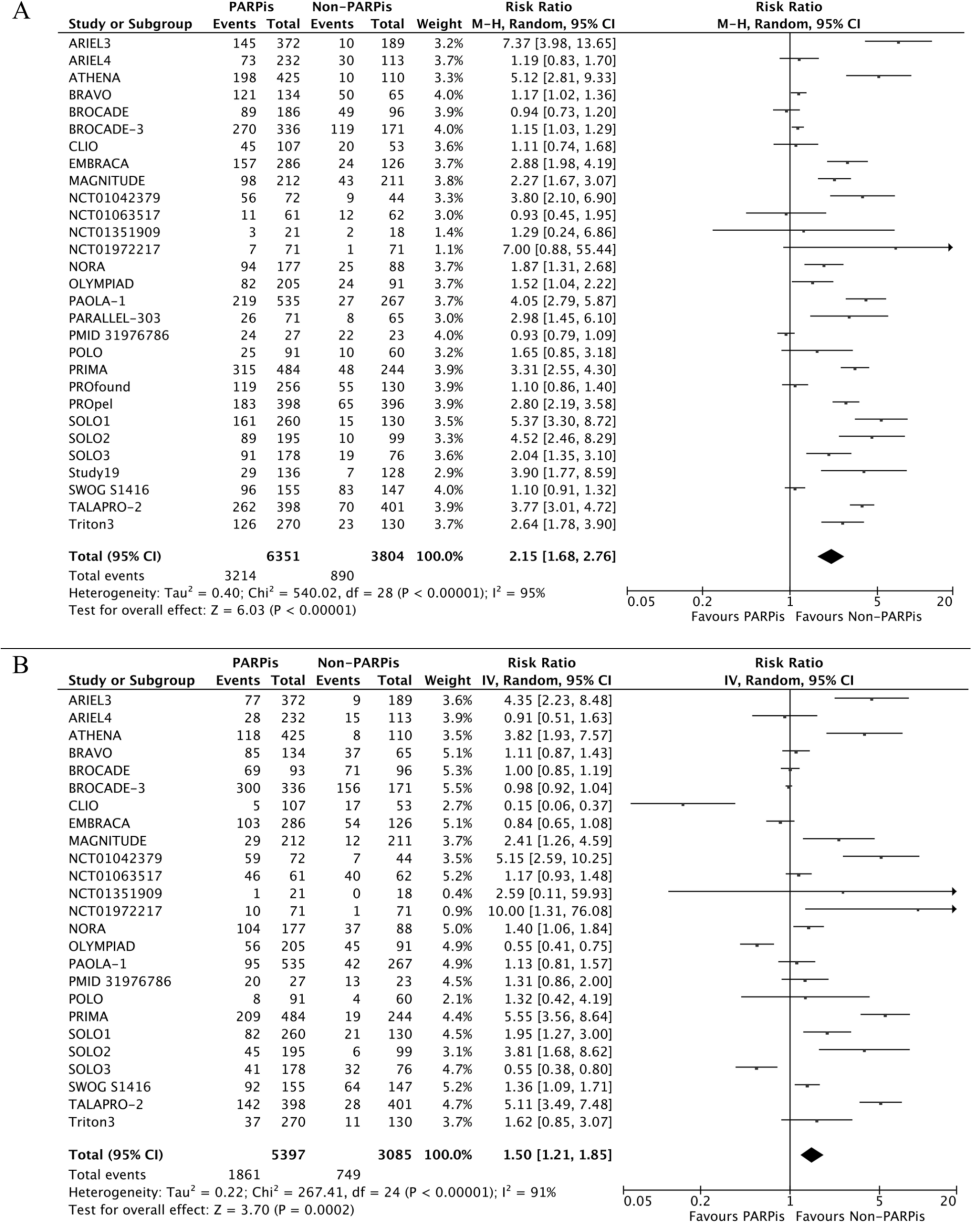

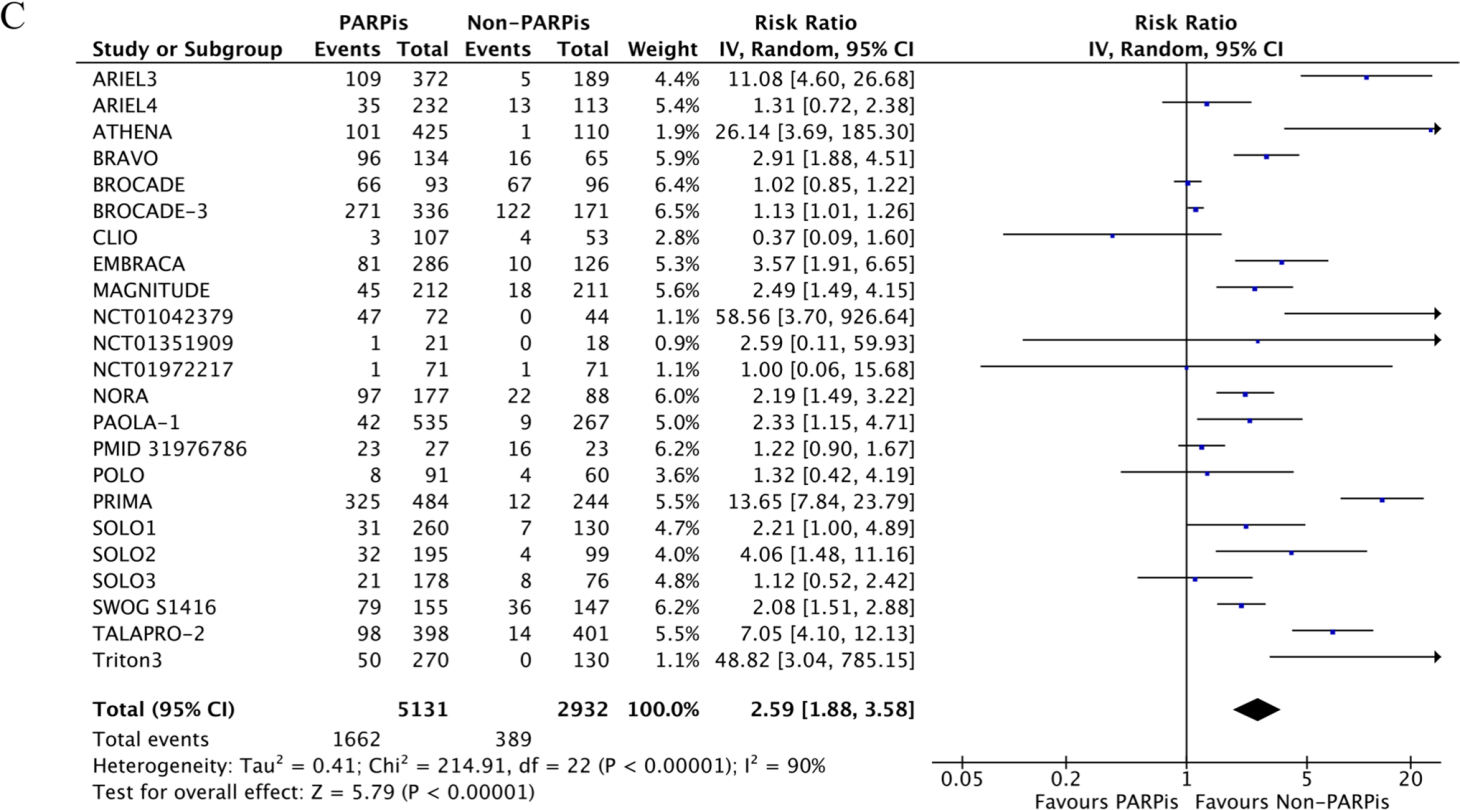
All-grades anemia (**A**), neutropenia (**B**), and thrombocytopenia (**C**) of PARPis compared with non-PARPis. CI: confidence interval

Anemia was the most common AE with PARPis, with a pooled incidence of 49.2%, followed by neutropenia (32.3%) and thrombocytopenia (30.1%). The administration of PARPis significantly increased the risk of anemia (RR 2.15, 95% 1.68–2.76; p < 0.00001), neutropenia (RR 1.50, 95% CI 1.21–1.85; p = 0.0002), and thrombocytopenia (RR 2.59, 95% CI 1.88–3.58; p < 0.00001). A significantly high heterogeneity was evident in all the analyses (Fig. 2A-C).

### Severe hematological toxicity

**Fig. 3:**
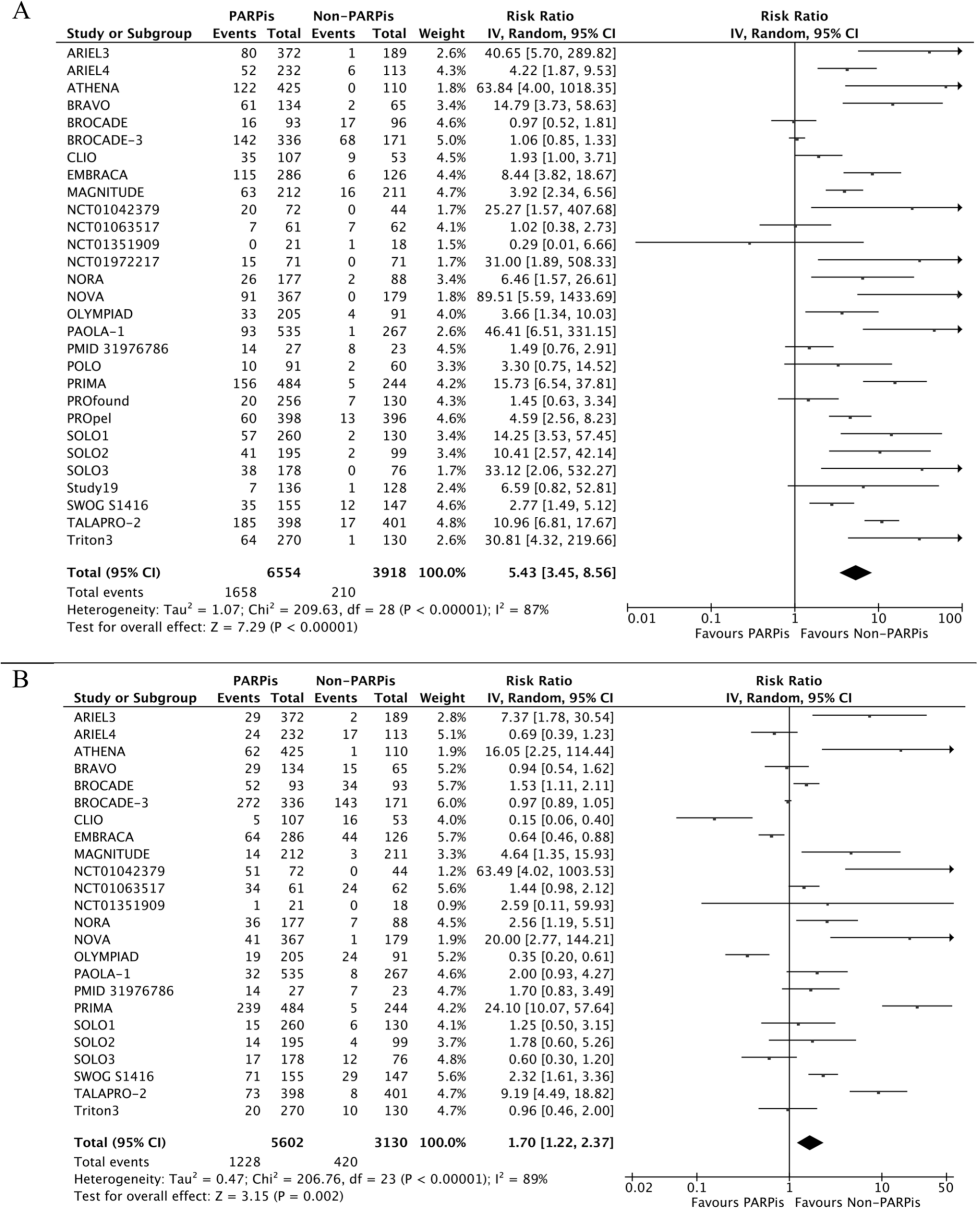

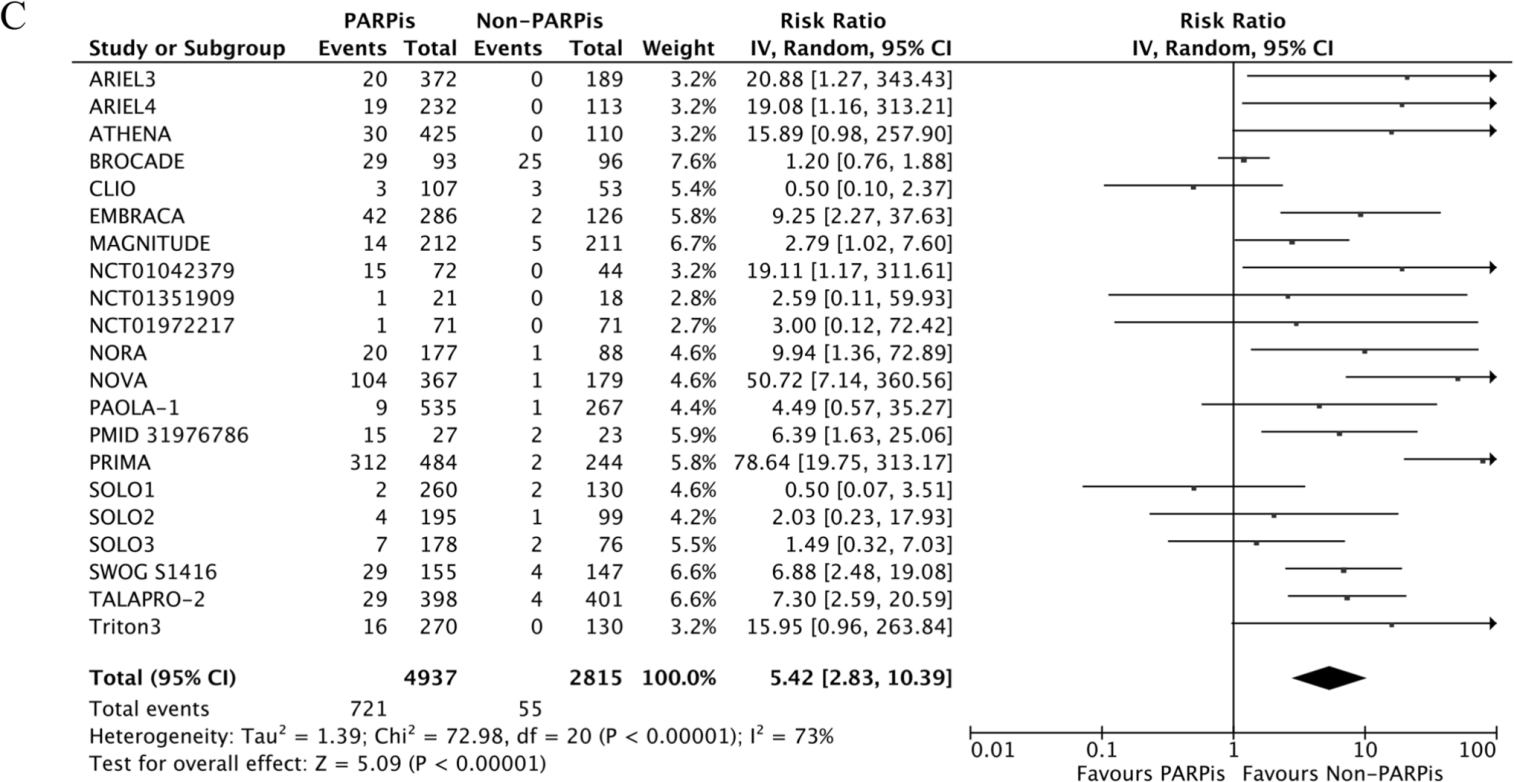
≥G3 anemia (**A**), neutropenia (**B**), and thrombocytopenia (**C**) of PARPis compared with non-PARPis. CI: confidence interval.

Among ≥ G3 AEs, anemia was also the most frequent. We observed a pooled incidence of 25% for ≥ G3 anemia, 18.7% for ≥ G3 neutropenia, and 11% for ≥ G3 thrombocytopenia. Patients treated with PARPis were at significantly higher risk of developing anemia (RR 5.43, 95% CI 3.45–8.56; p < 0.00001), neutropenia (RR 1.70, 95% CI 1.22–2.37; p = 0.002), and thrombocytopenia (RR 5.42, 95% CI 2.83–10.39; p < 0.00001). High heterogeneity was retrieved among the studies for all the outcomes (Fig. 3A-C).

### Acute myeloid leukemia/myelodysplastic syndrome

**Fig. 4:**
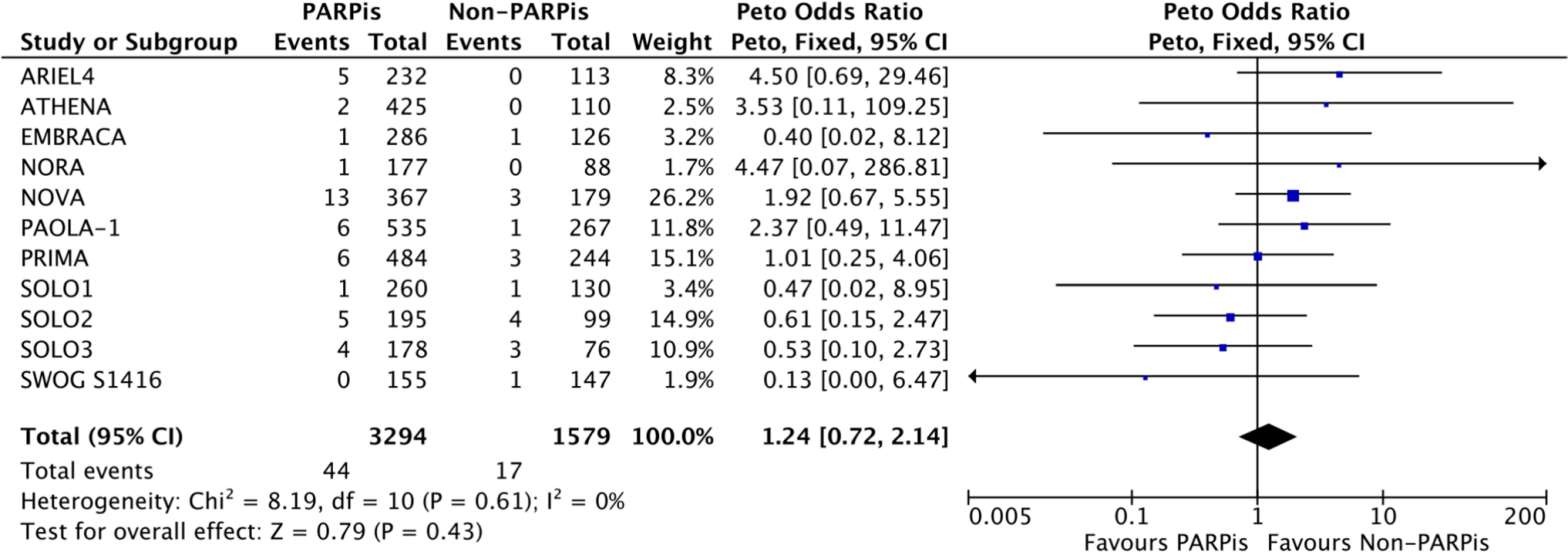
AML/MDS of PARPis compared with non-PARPis. CI: confidence interval

The administration of PARPis did not significantly increase the risk of AML/MDS compared to non-PARPis (POR 1.24, 95% CI 0.72–2.14; p = 0.43). There was no heterogeneity among the included studies (Fig. 4).

### Subgroup and sensitivity analyses

To explore potential sources of heterogeneity, we conducted subgroup analyses based on: the type of drug, cancer type, monotherapy vs. combinational treatment, the comparator group, treatment-naive vs. pre-treated patients, and treatment duration (> 12 or ≤ 12 months). The relative risks (RRs) for each subgroup, along with *p*-values and *I*^*2*^ for overall subgroup differences, are presented in Table 3 for both all grades and high-grade hematological toxicity.

**Table 3:** Subgroup analyses for hematological toxicity of PARPis in solid tumors. Statistically significant differences are bolded

Subgroup	Anemia, *RR (95% CI)*	Neutropenia, *RR (95% CI)*	Thrombocytopenia, *RR (95%CI)*	≥ G3 Anemia, *RR (95%CI)*	≥ G3 Neutropenia, *RR (95% CI)*	≥ G3 Thrombocytopenia, *RR (95% CI)*
*Type of PARPi*						
Olaparib	2.45 (1.72–3.47)	1.03 (0.65–1.63)	1.68 (1.02–2.77)	4.79 (2.48–9.26)	0.80 (0.41–1.54)	1.21 (0.55–2.64)
Niraparib	2.01 (1.05–3.84)	2.10 (0.98–4.46)	3.79 (1.64–8.74)	10.51 (3.77–29.30)	5.19 (1.07–25.21)	17.60 (2.03–152.65)
Rucaparib	3.21 (1.39–7.41)	2.20 (1.03–4.70)	9.55 (1.34–68.22)	19.83 (3.38–116.36)	2.39 (0.13–43.61)	17.83 (4.40–72.20)
Talazoparib	3.44 (2.68–4.42)	2.06 (0.31–13.61)	5.11 (2.62–9.95)	10.23 (6.80–15.40)	2.36 (0.62–9.00)	7.94 (3.45–18.27)
Veliparib	1.21 (0.96–1.52)	1.38 (0.98–1.94)	1.37 (0.98–1.93)	1.47 (0.87–2.49)	1.62 (0.90–2.93)	4.14 (1.17–14.61)
*Subgroup differences*	*P* < *0.00001* *I*^*2*^ = *89.8%*	*P* = *0.33* *I*^*2*^ = *7.5%*	*P* = *0.002* *I*^*2*^ = *77.0%*	*P* < *0.00001* *I*^*2*^ = *89.2%*	*P* = *0.17* *I*^*2*^ = *37.4%*	*P* = *0.001* *I*^*2*^ = *77.5%*
*Type of cancer*						
Ovarian cancer	2.96 (2.03–4.33)	1.57 (0.91–2.72)	3.03 (1.49–6.14)	12.46 (5.43–28.59)	2.37 (0.90–6.23)	5.58 (1.49–20.88)
Breast cancer	1.46 (1.16–1.85)	1.07 (0.86–1.33)	2.01 (1.24–3.25)	3.05 (1.28–7.25)	0.99 (0.68–1.45)	4.60 (1.16–18.26)
Pancreatic cancer	1.20 (0.44–3.33)	1.31 (0.88–1.95)	1.23 (0.91–1.66)	1.71 (0.92–3.20)	1.70 (0.83–3.49)	6.39 (1.63–25.06)
Gastric cancer	0.93 (0.45–1.95)	NA	NA	1.02 (0.38–2.73)	NA	NA
Prostate cancer	2.44 (1.54–3.84)	3.15 (1.58–6.27)	4.66 (1.62–13.38)	5.73 (2.72–12.04)	1.63 (1.13–2.36)	4.71 (2.38–9.30)
*Subgroup differences*	*P* = *0.005* *I*^*2*^ = *73.3%*	*P* = *0.02* *I*^*2*^ = *68.8%*	*P* = *0.01* *I*^*2*^ = *72.2%*	*P* = *0.0002* *I*^*2*^ = *81.5%*	*P* = *0.14* *I*^*2*^ = *44.7%*	*P* = *0.92* *I*^*2*^ = *0%*
*PARPis mono vs combo*						
Monotherapy	2.34 (1.65–3.32)	1.37 (0.90–2.08)	3.36 (1.75–6.46)	8.28 (4.55–15.06)	1.57 (0.77–3.20)	7.02 (2.04–24.14)
PARPi + bevacizumab	4.05 (2.79–5.87)	1.13 (0.81–1.57)	2.33 (1.15–4.71)	46.41 (6.51–331.15)	2.00 (0.93–4.27)	4.49 (0.57–35.27)
PARPi + CHT	1.18 (0.96–1.46)	1.32 (1.00–1.74)	1.37 (0.98–1.93)	1.40 (0.89–2.20)	1.55 (0.95–2.54)	4.14 (1.17–14.61)
PARPi + ARTA	2.97 (2.25–3.92)	4.04 (2.11–7.72)	3.62 (1.38–9.49)	6.38 (3.29–12.39)	7.74 (4.16–14.37)	4.36 (2.16–8.80)
*Subgroup differences*	*P* < *0.00001* *I*^*2*^ = *93.5%*	*P* = *0.007* *I*^*2*^ = *75.1%*	*P* = *0.04* *I*^*2*^ = *64.6%*	*P* < *0.00001* *I*^*2*^ = *91.1%*	*P* = *0.0004* *I*^*2*^ = *83.6%*	*P* = *0.92* *I*^*2*^ = *0%*
*Comparator group*						
PBO	3.41 (2.76–4.21)	2.79 (1.79–4.34)	4.07 (2.32–7.15)	10.33 (6.13–17.39)	5.03 (2.40–10.53)	7.47 (2.52–22.15)
CHT	1.44 (1.17–1.75)	0.97 (0.81–1.16)	1.62 (1.14–2.31)	3.00 (1.66–5.42)	0.88 (0.66–1.16)	3.71 (1.52–9.07)
ARTA	1.49 (0.75–2.93)	16.88 (1.05–271.13)	22.73 (1.42–363.39)	5.42 (0.12–236.25)	9.23 (0.57–150.5)	7.43 (0.45–122.12)
*Subgroup differences*	*P* < *0.00001* *I*^*2*^ = *94.3%*	*P* < *0.0001* *I*^*2*^ = *91.1%*	*P* = *0.006* *I*^*2*^ = *80.3%*	*P* = *0.009* *I*^*2*^ = *78.8%*	*P* < *0.00001* *I*^*2*^ = *90.5%*	*P* = *0.60* *I*^*2*^ = *0%*
*Treatment line*						
Naive	2.69 (1.73–4.18)	2.30 (1.35–3.93)	4.12 (1.87–9.08)	7.68 (4.15–14.19)	4.41 (1.35–14.44)	6.52 (2.72–15.62)
Pre-treated	1.71 (1.35–2.17)	1.21 (0.95–1.53)	1.90 (1.27–2.84)	4.80 (2.34–9.86)	1.08 (0.79–1.48)	4.27 (1.49–12.24)
*Subgroup differences*	*P* = *0.08* *I*^*2*^ = *68.0%*	*P* = *0.03* *I*^*2*^ = *78.8%*	*P* = *0.09* *I*^*2*^ = *65.9%*	*P* = *0.33* *I*^*2*^ = *0%*	*P* = *0.02* *I*^*2*^ = *80.2%*	*P* = *0.54* *I*^*2*^ = *0%*
*Treatment duration*						
> 12 months	3.87 (3.14–4.76)	2.70 (1.38–5.28)	4.25 (2.13–8.47)	11.16 (5.56–22.38)	3.27 (1.26–8.45)	3.51 (1.13–10.87)
≤ 12 months	2.24 (1.56–3.23)	1.18 (0.76–1.83)	2.75 (1.15–6.56)	5.65 (2.65–12.07)	0.85 (0.53–1.36)	7.15 (2.30–22.16)
*Subgroup differences*	*P* = *0.01* *I*^*2*^ = *84.6%*	*P* = *0.04* *I*^*2*^ = *75.8%*	*P* = *0.44* *I*^*2*^ = *0%*	*P* = *0.20* *I*^*2*^ = *40.4%*	*P* = *0.01* *I*^*2*^ = *84.0%*	*P* = *0.38* *I*^*2*^ = *0%*

ARTA: androgen receptor-targeted agents; CHT: chemotherapy; CI: confidence interval; ≥ G3: equal to over grade 3; NA: not available; PARPi: Poly (ADP-ribose) polymerase inhibitor; PBO: placebo; RR: relative risk.

In the all grades hematological toxicity analysis, we observed statistically significant variations in terms of anemia (p < 0.00001) and thrombocytopenia (p = 0.001) concerning the type of PARPi, even if the heterogeneity remained generally high. Notably, niraparib exhibited the lowest statistically significant RRs for anemia (2.01), and olaparib for thrombocytopenia (1.68), whereas talazoparib determined the highest risk of anemia (3.44), and rucaparib of neutropenia (2.20) and thrombocytopenia (9.55). Moreover, the specific type of combination drug used in the RCTs showed significant differences for all grades AEs, with the highest RRs for PARPis + bevacizumab, even though with confirmed high heterogeneity.

Furthermore, for different cancer types, we noted statistically significant differences in anemia (p = 0.005), neutropenia (p = 0.02), and thrombocytopenia (p = 0.01), with prostate cancer having the highest risk of neutropenia (3.15) and thrombocytopenia (4.66). Also, in this analysis, the heterogeneity among the studies remained high. The treatment line statistically influenced only all grades of neutropenia (p = 0.03).

Significant differences were observed in the risk ratios (RRs) of all-grade anemia (p < 0.00001), neutropenia (p = 0.0001), and thrombocytopenia (p = 0.004) across comparator groups (PBO, CHT, ARTA). Additionally, the duration of treatment showed significant differences for all-grade anemia (p < 0.001) and neutropenia (p = 0.04), with patients receiving PARPis for ≤ 12 months having the lowest risk for anemia (RR 2.21) and neutropenia (RR 1.15).

In the high-grade toxicities analysis, the choice of PARPi significantly differed for anemia (p < 0.00001) and thrombocytopenia (p = 0.001), with olaparib exhibiting the lowest statistically significant RRs for high-grade anemia (4.79). We also found significant differences in subgroup cancer type (p = 0.0002), monotherapy vs. combination therapy (p < 0.00001), and comparator group (p = 0.009) concerning the risk of anemia, while monotherapy vs. combination therapy (p = 0.0004), treatment duration (p = 0.01), naïve versus pre-treated patients (p = 0.02), and the comparator arm (p < 0.00001) showed significant differences for neutropenia (Table 3).

The sensitivity analysis, which removed one study at a time, did not reveal a different impact of the included studies on the overall effects for all AEs (Supplementary Fig. 1).

**Fig. 5:**
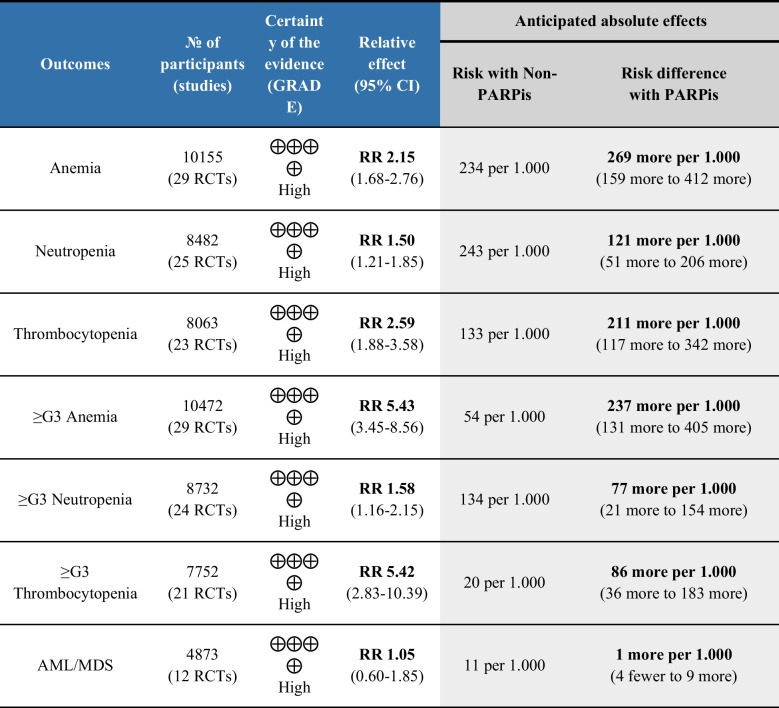
Summary of Findings of the systematic review and meta-analysis. AML/MDS: acute myeloid leukemia/myelodysplastic syndrome; CI: confidence interval; RCT: randomized controlled trial; RR: relative risk; ≥ G3: equal to over grade 3

The certainty of evidence was assessed using the GRADE approach, considering risk of bias, inconsistency, indirectness, imprecision, and publication bias across outcomes. Given the high certainty of the selected evidence, we are confident that the true effect lies close to our estimate of the effect (Fig. 5).

## Discussion

PARP inhibitors have become significant players in the Golden Age of personalized oncology. Over the past decade, multiple PARPis have received approval for clinical use across various tumor subtypes, and ongoing research is exploring new agents and combinations with other drugs. Unlike traditional chemotherapy, PARPis have a distinct safety profile. However, they are not without side effects. Hematologic adverse events have been consistently recognized as the most prevalent adverse effects, often leading to treatment discontinuation, interruption, or dose reduction in patients receiving PARP inhibitors [8].

Our meta-analysis, which includes the largest number of published studies involving these agents to date, compiles data from 31 RCTs and over 10,000 patients with various solid tumors treated with PARPis. It confirms and updates the existing literature. Specifically, the use of PARPis is associated with a significant increase in the risk of developing all-grade (1–5) and severe (≥ 3) anemia, neutropenia, and thrombocytopenia, with an increase ranging from 77 to 269 more cases for every 1,000 treated patients. In contrast, the risk of AML/MDS appears not to be elevated.

Indeed, even if some studies presented some risks of bias, the removal of a single study at a time during the sensitivity analysis did not impact the global effects of the estimates for each AE (Table 1, Supplementary Fig. 1A-F).

PARP-1 trapping has been investigated as a potential cause of bone marrow toxicity. In ex vivo colony assays using hematopoietic progenitors derived from human bone marrow, it was evident that there was a variation between the cytotoxic effects and bone marrow toxicity [9]. While all PARPis act similarly, they exhibit slight differences in their activity profiles. For instance, olaparib and rucaparib had a > 100-fold difference in IC50 values between PARP inhibition and bone marrow toxicity. In contrast, talazoparib had only a two-fold difference, suggesting that the therapeutic effect of the latter is closely aligned with its toxic activity [10]. In our subgroup analysis, the highest relative risk (RR) for anemia was associated with talazoparib. However, further studies are needed to explore this observation. Nonetheless, in other reports, this effect appears relevant only when PARPis are combined with other cytotoxic agents, such as carboplatin [11].

In our analysis, PARPis exhibited their toxic effects both when used as single agents and in combination with other drugs. In addition to PARP-1, a critical role in developing hematologic adverse events, particularly anemia, is attributed to PARP-2, as its inhibition has been linked to chronic anemia in animal models [12]. In the TALAPRO-2 trial, a median hemoglobin decrease of 2 g/dL was observed in the talazoparib + enzalutamide group, leading to 13.1% of patients receiving red blood transfusions and 8.3% receiving erythropoietin-stimulating agents [13, 14]. Indeed, in PARP-2 deficient mouse models, PARP-2 inhibition was shown to impair the differentiation of erythroid progenitors and shorten the lifespan of erythrocytes, suggesting that exogenous supplementation may not be the most effective means of managing anemia [12, 15, 16].

Regarding thrombocytopenia, PARPis reversibly hinder megakaryocyte maturation and proliferation [17]. In ovarian cancer studies, the decrease in platelet count begins during the first cycle and plateaus after the second or third cycle of PARPis [18–20].

Hematological toxicity typically occurs shortly after the initiation of PARP inhibitors (PARPis). In addition to the previously discussed ovarian cancer trials, the PROfound study showed that in metastatic castration-resistant prostate cancer (mCRPC), anemia peaked two months after starting olaparib and lasted for an additional two months. Severe hematologic adverse events also appeared within six months of starting talazoparib in the TALAPRO-2 study. Similarly, anemia and other adverse events surfaced early across various tumor contexts, peaking several months into PARPis treatment. Guidelines recommend obtaining baseline blood counts before and during treatment with all approved PARPis. A differential diagnosis for anemia should include identifying other potential causes, such as measuring iron, folate, and vitamin B12 levels. If hemoglobin drops below 8 g/dL, PARPis should be paused until levels rise above 9 g/dL, and blood transfusions are advised if hemoglobin falls below 7 g/dL. PARPis may be resumed once anemia resolves, with dosage adjustments based on the presence of symptoms. In cases of greater than grade 3 (> G3) neutropenia, PARPis should be interrupted and can be resumed only if toxicity resolves at a reduced dosage. Platelet transfusions are recommended for thrombocytopenia when platelet counts fall below 10 × 10^9/L. A dose reduction or interruption is advised when platelet counts drop below 50–100 × 10^9/L or if bleeding occurs. Concomitant medications should be re-evaluated for additional risk. Studies on OC recommend lowering the starting dosage of niraparib for patients with a baseline weight below 77 kg or a platelet count under 100 × 10^9/L. If severe hematological toxicity persists for more than four weeks, patients should be referred to a hematologist for a bone marrow evaluation to reduce the risk of developing acute myeloid leukemia/myelodysplastic syndrome (AML/MDS) [21].

The subgroup analysis based on treatment duration revealed intriguing findings: Patients who received PARPIs for 12 months or fewer were at a lower risk of all-grade anemia, neutropenia, and ≥ G3 neutropenia.

In analyzing the combination of drugs used with PARPis, it was found that ARPI had the most favorable outcomes, exhibiting the lowest rates of all-grade and high-grade anemia and neutropenia. In contrast, bevacizumab demonstrated the lowest rate of all-grade thrombocytopenia.

It is worth noting that pretreated patients displayed lower RRs for all-grade anemia and thrombocytopenia than treatment-naive patients. While this conclusion should be taken cautiously, it indicates the possibility of safely using PARPis even in pretreated patients.

Our systematic review and meta-analysis represent the most up-to-date evaluation of PARPis hematological toxicity. The previous meta-analysis on this subject covered studies published before September 2020. We included a larger number of studies and patients with longer follow-ups. However, our meta-analysis has several limitations. Firstly, there is heterogeneity among the included studies, with variations in tumor types, treatment settings, types of PARPis, dosages, and combinations. We conducted subgroup analyses to explore potential sources of heterogeneity. Nonetheless, some variables may not have been considered in our analysis. Another limitation is the use of aggregate data rather than individual patient data.

## Conclusions

In summary, our updated systematic review and meta-analysis confirms that PARP inhibitors significantly increase the risk of hematologic AEs across all grades. This risk is particularly prominent in ovarian, breast, and prostate cancers. Still, it remains consistent across various factors, including treatment duration, combination with different drugs, and the distinction between frontline and pre-treated settings. Therefore, healthcare providers should be vigilant about this risk and carefully monitor patients receiving PARPis.

## Supplementary Information

Below is the link to the electronic supplementary material.

Supplementary file1 (DOCX 63887 KB)

## Data Availability

No datasets were generated or analysed during the current study.
