# Supplementary material for: Hematological toxicity of parp inhibitors in solid tumors: a systematic review and safety meta-analysis
Source: Cancer Metastasis Rev. 2025 Aug 14;44(3):65. doi: 10.1007/s10555-025-10283-1 (PMC12350551; doi:10.1007/s10555-025-10283-1)
Supplement: Supplementary file 1 — Supplementary file1 (DOCX 63887 KB) [file 10555_2025_10283_MOESM1_ESM.docx]

|  | Query |
| --- | --- |
| PubMed as an example | "randomized clinical trial"[Publication Type] OR "controlled clinical trial"[Publication Type] OR "randomized controlled trial"[Title/Abstract] OR "RCT"[Title/Abstract] AND "PARP inhibitor" [Title/Abstract] OR "Poly (ADP-ribose) Polymerase Inhibitors" [Title/Abstract] OR “PARPi” [Title/Abstract] "olaparib" [Title/Abstract] OR "niraparib" [Title/Abstract] OR "rucaparib" [Title/Abstract] OR "talazoparib"[Title/Abstract] OR "veliparib" [Title/Abstract] OR "Fluzoparib" [Title/Abstract] AND “ovarian cancer” [Title/Abstract] OR “ovarian carcinoma” [Title/Abstract] OR “prostate cancer” [Title/Abstract] OR “prostate carcinoma” [Title/Abstract] OR “gastric cancer” [Title/Abstract] OR “gastric carcinoma” [Title/Abstract] OR “breast cancer” [Title/Abstract] OR “breast carcinoma” [Title/Abstract] OR “pancreatic cancer” [Title/Abstract] OR “pancreatic carcinoma” [Title/Abstract] |

**Supplementary Table 1.** Search strategy for the databases based.

| **Population** | Patients with solid tumors |
| --- | --- |
| **Intervention** | PARPis |
| **Comparator** | Non-PARPis |
| **Outcome(s)** | RR with 95% CI of all-grades anemia, neutropenia, thrombocytopenia, and >G3 anemia, neutropenia, thrombocytopenia, (P)OR with 95% CI of AML/MDS |
| **Studies** | RCTs |

**Supplementary Table 2.** PICOS structure for study inclusion in the meta-analysis.

AML/MDS: acute myeloid leukemia/myelodysplastic syndrome; CI: confidence interval; mCRPC: metastatic castration-resistant prostate cancer; PARPis: Poly(ADP-ribose) Polymerase Inhibitors; (P)OR: Peto odds ratio; RCT: randomized clinical trials; RR: risk ratio

**Supplementary Figure 1.**

**Sensitivity analysis for: all-grades anemia (A), neutropenia (B), thrombocytopenia (C); severe anemia (D), neutropenia (E), thrombocytopenia (F).**

A

| 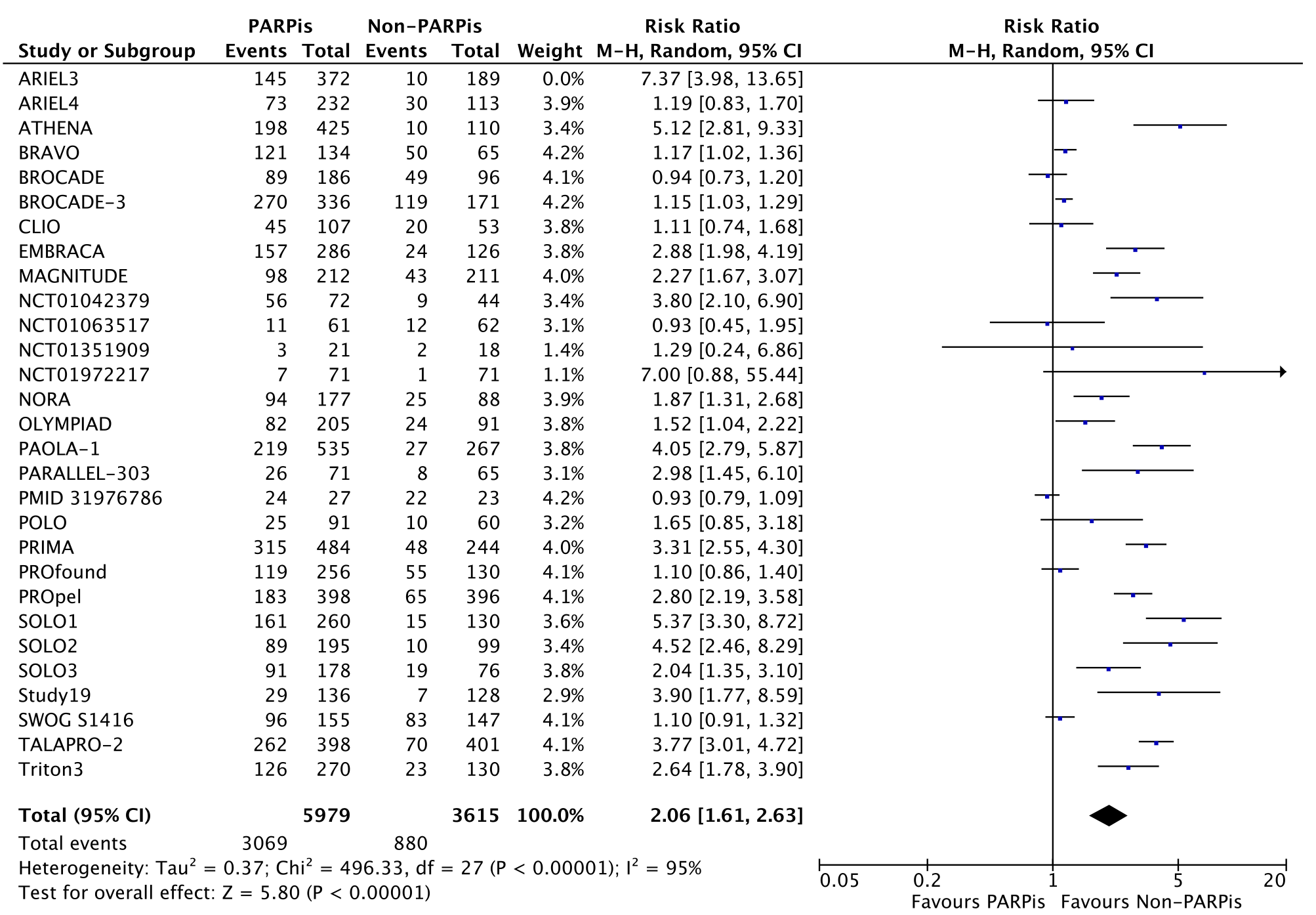 | 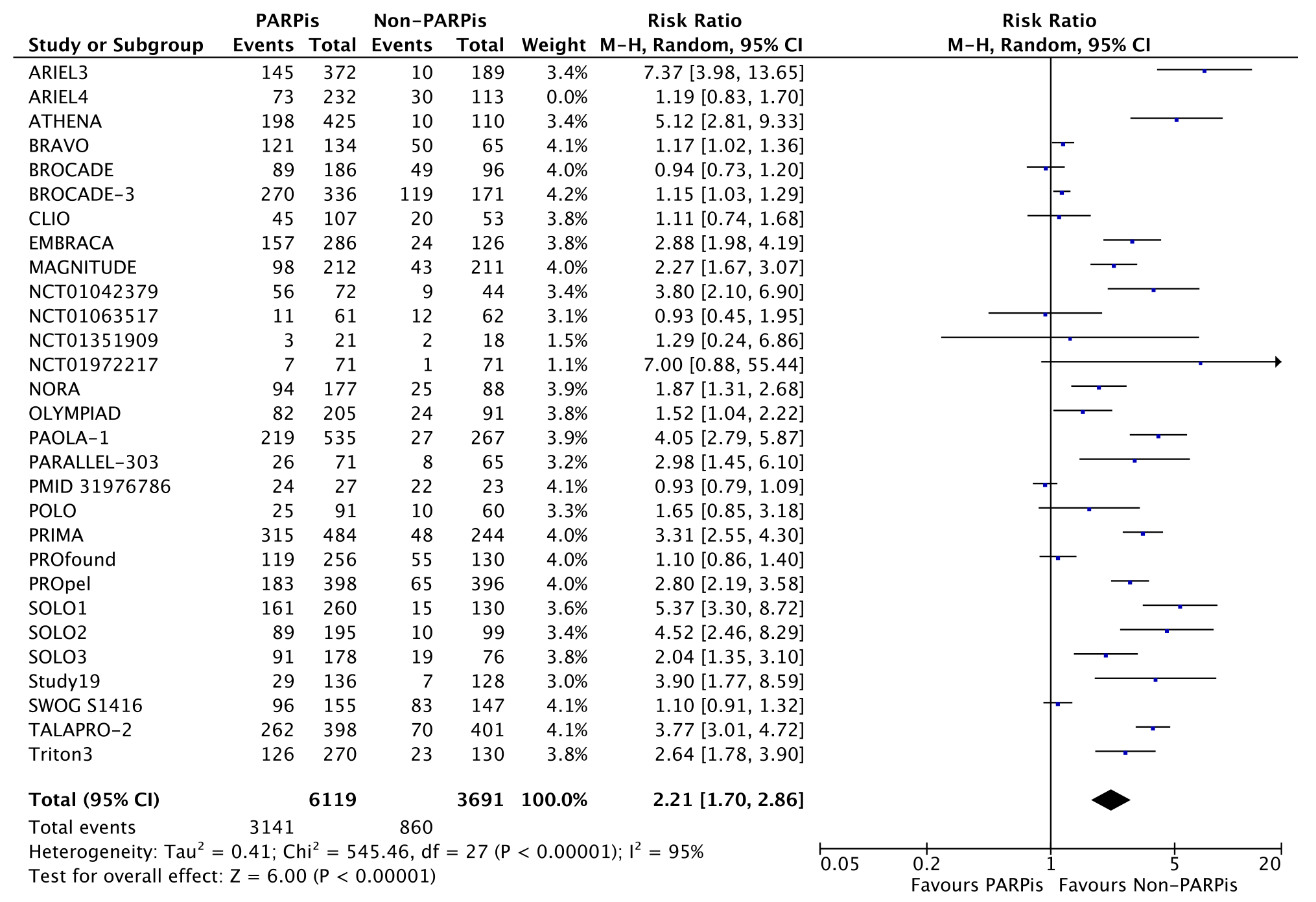 |
| --- | --- |
| 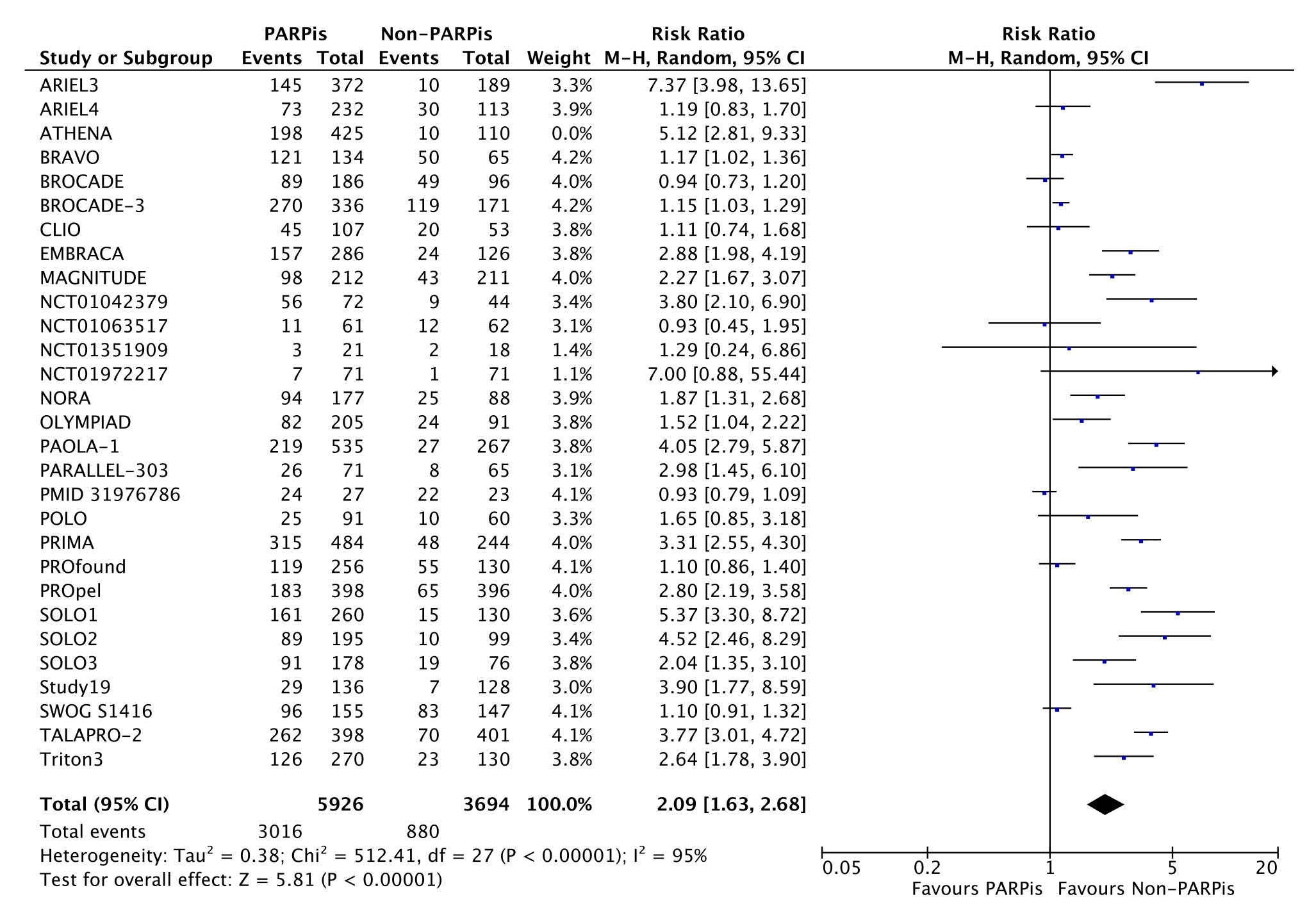 | 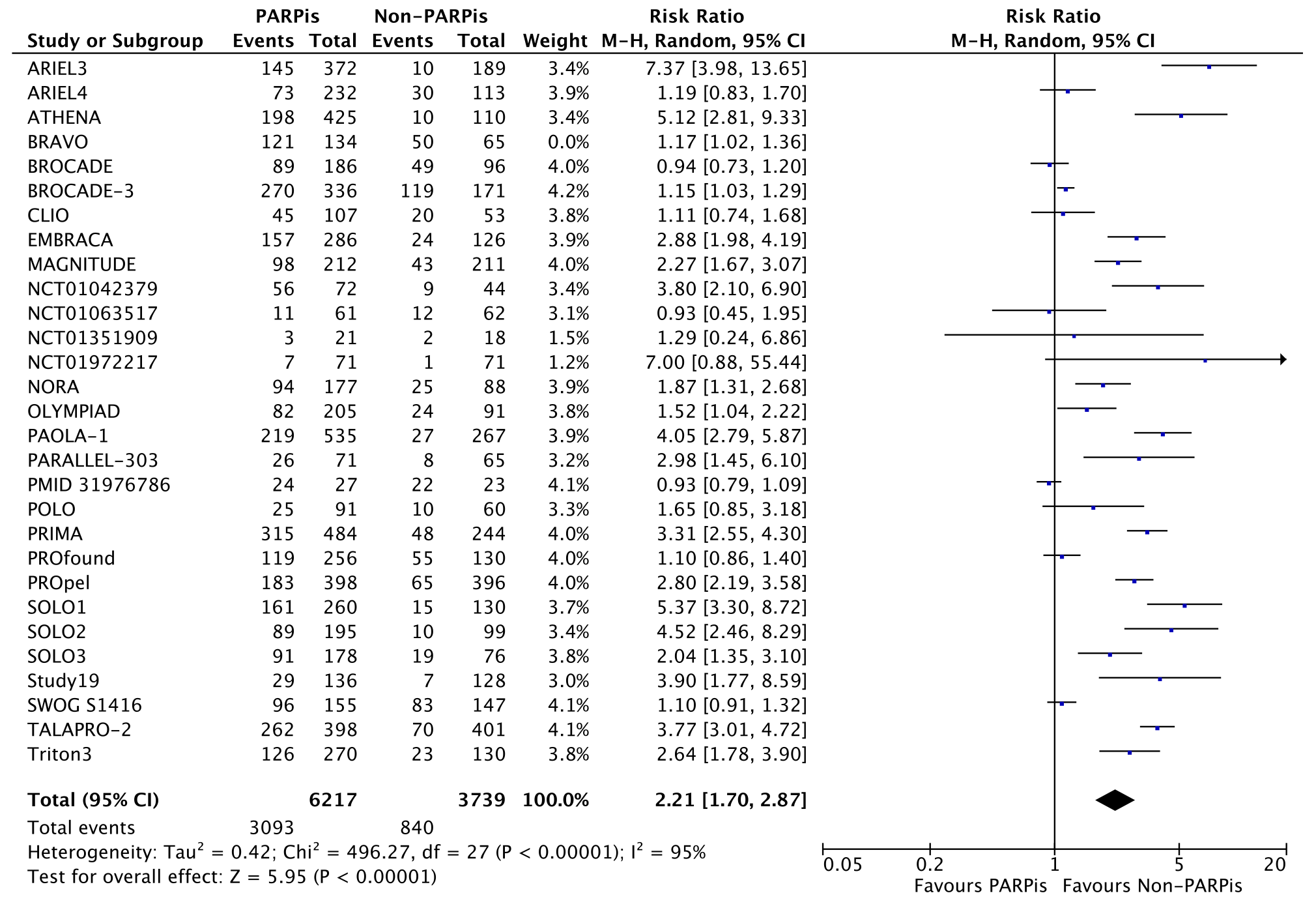 |
| 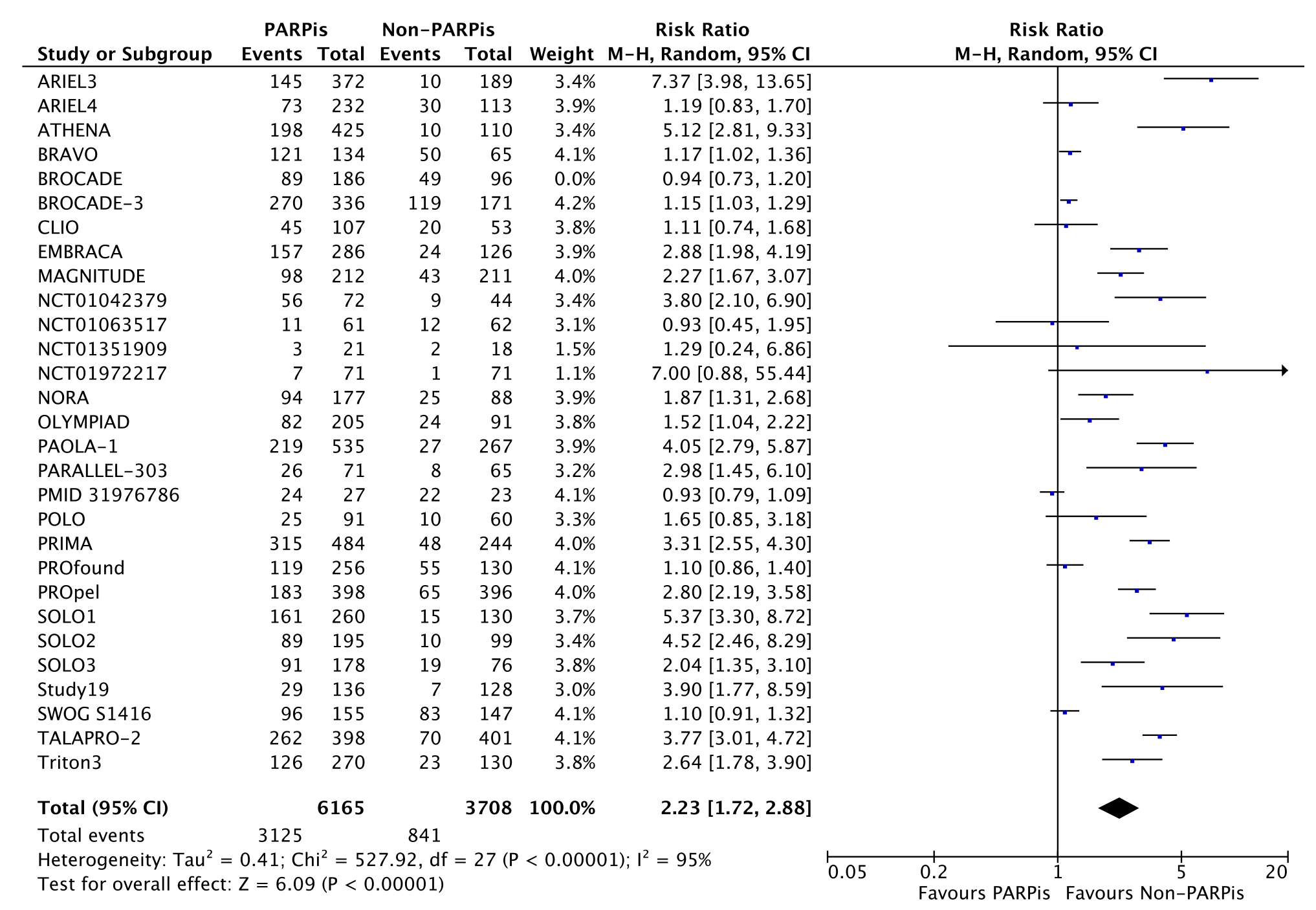 | 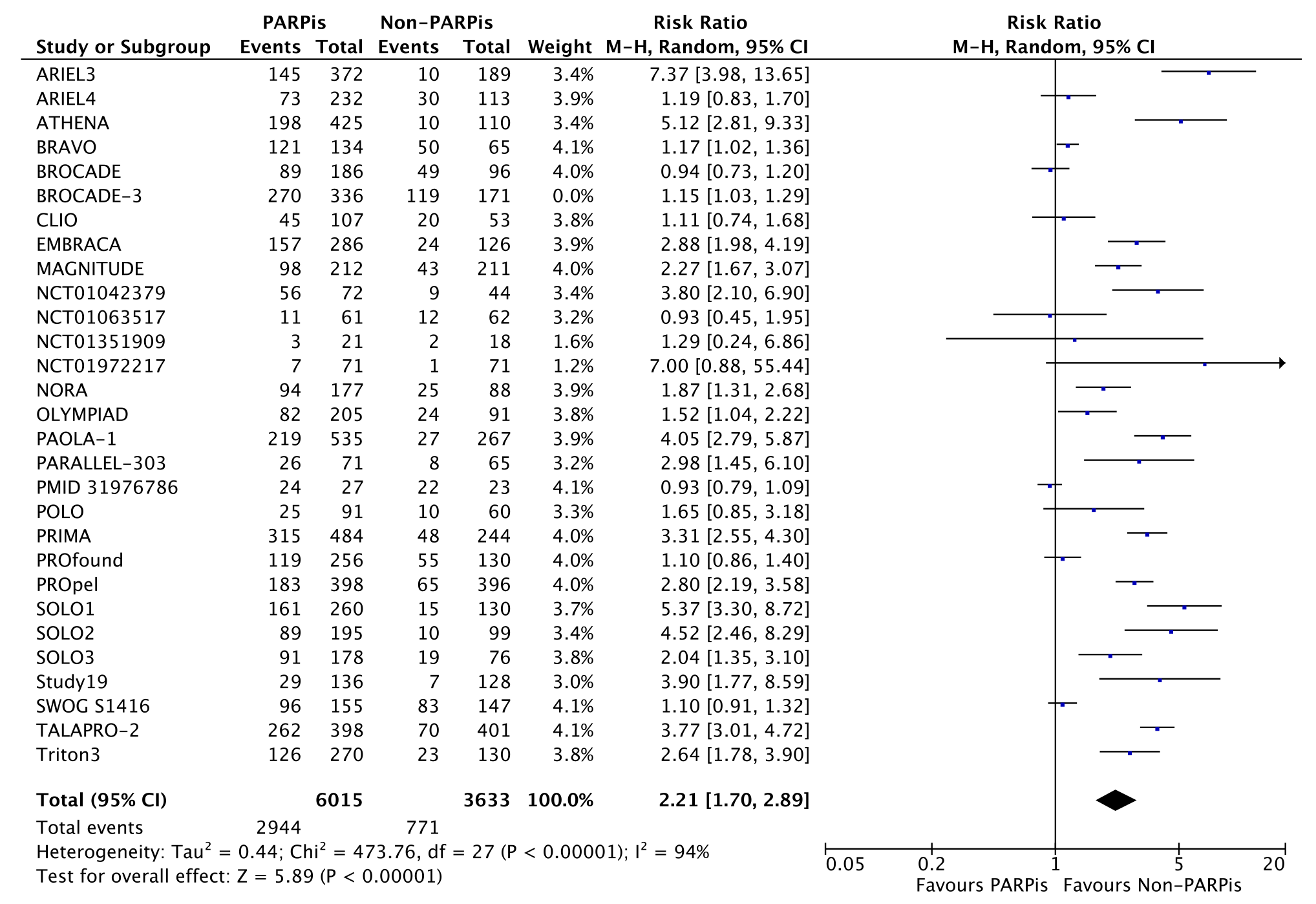 |
| 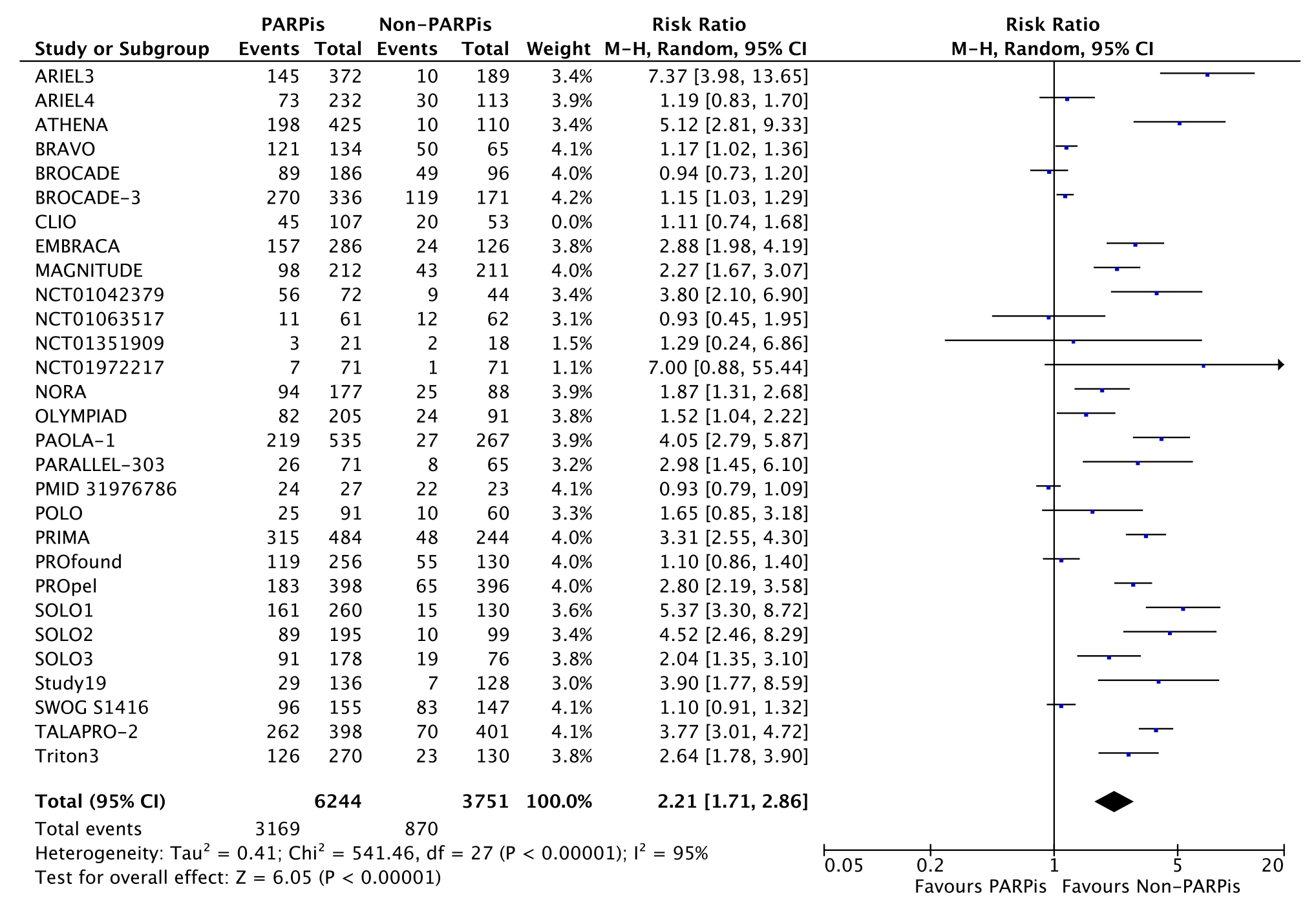 | 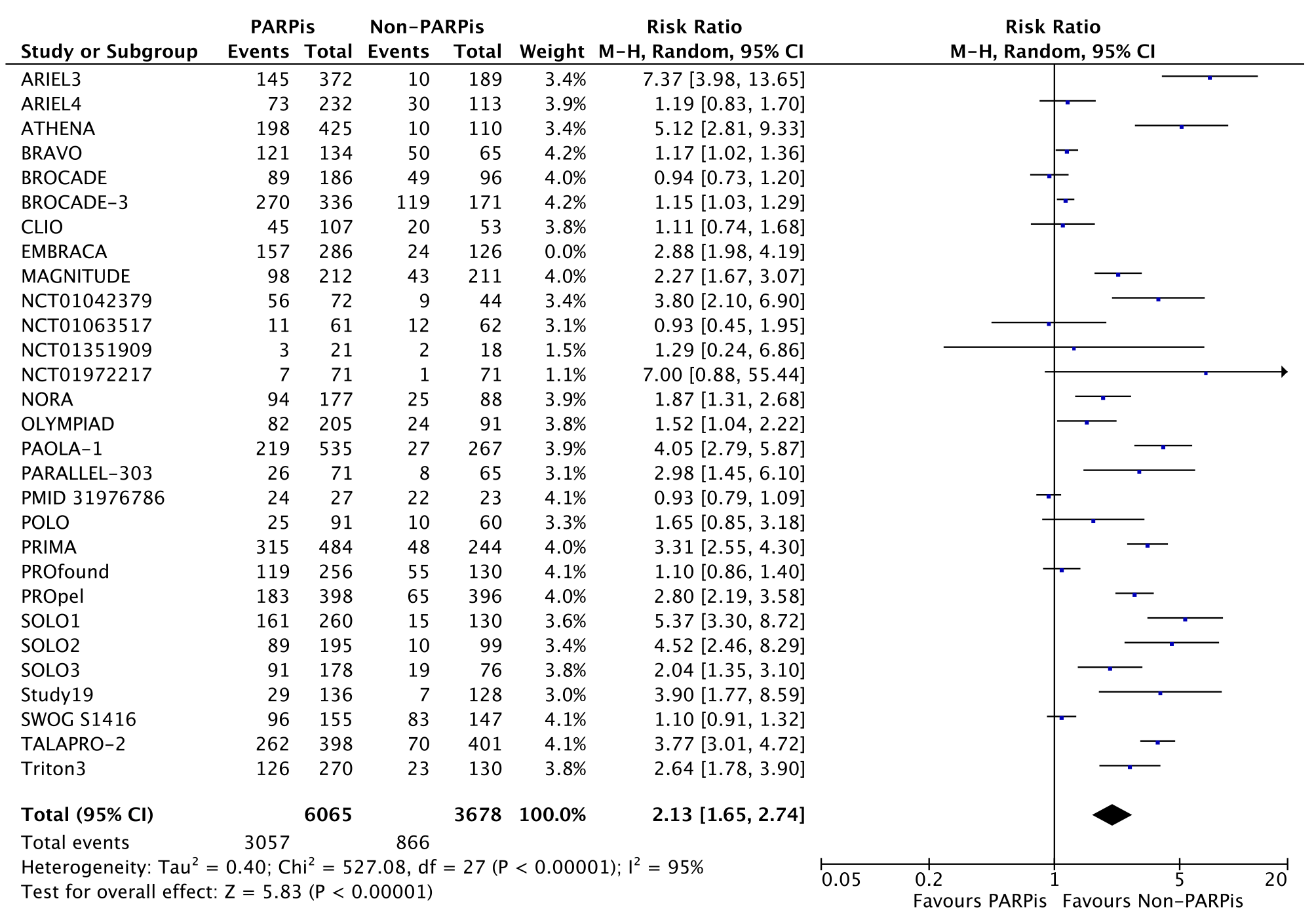 |
| 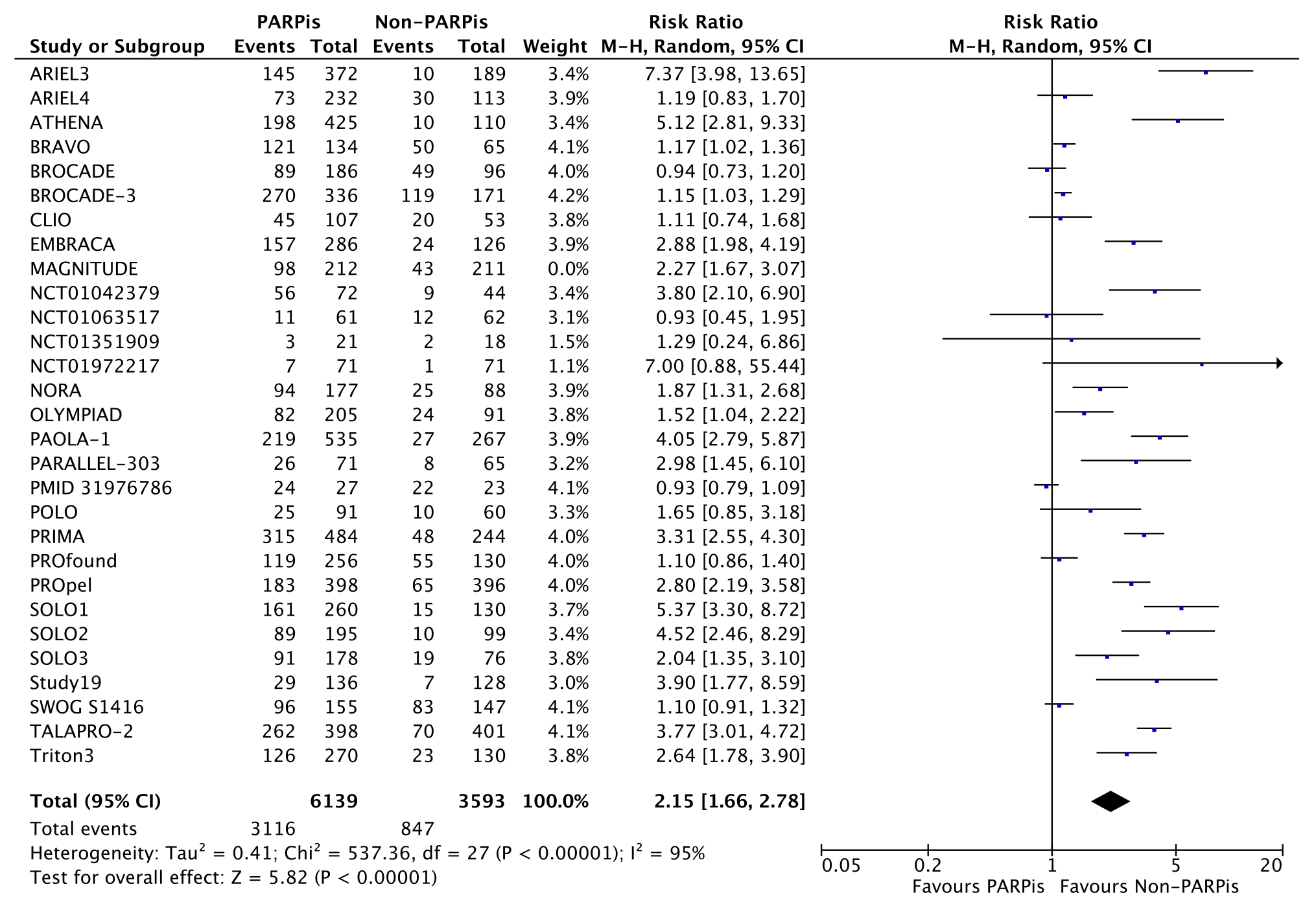 | 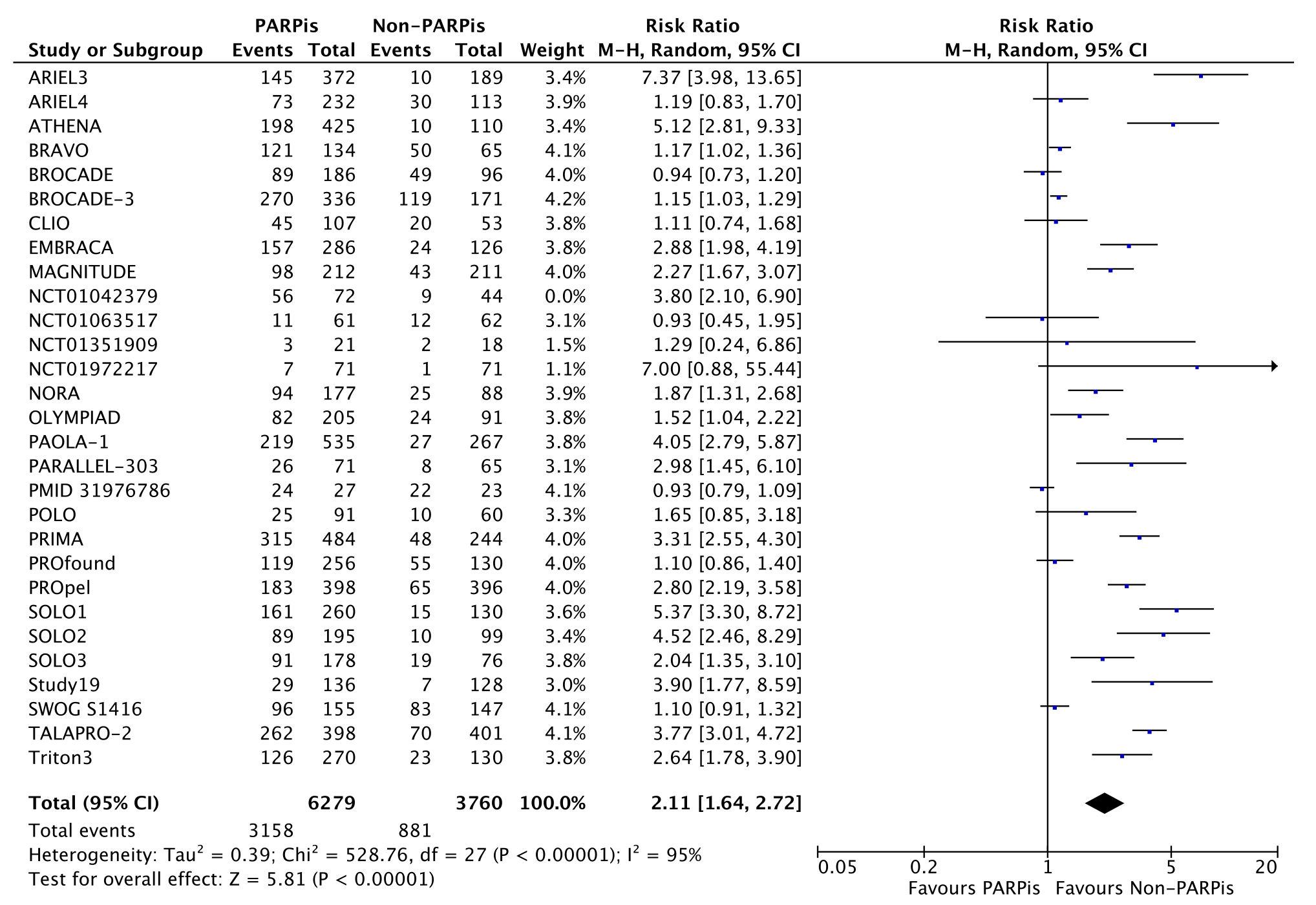 |
| 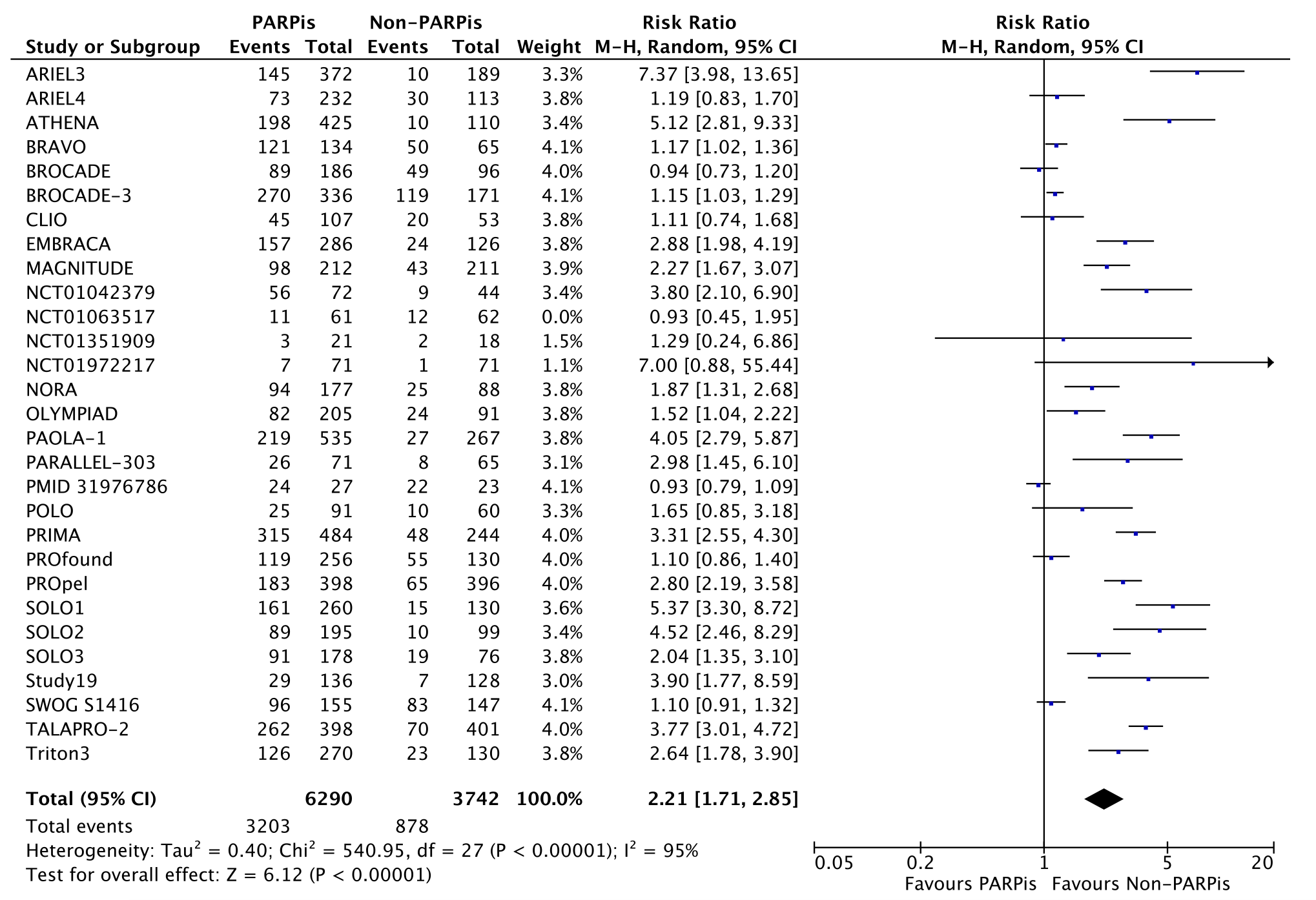 | 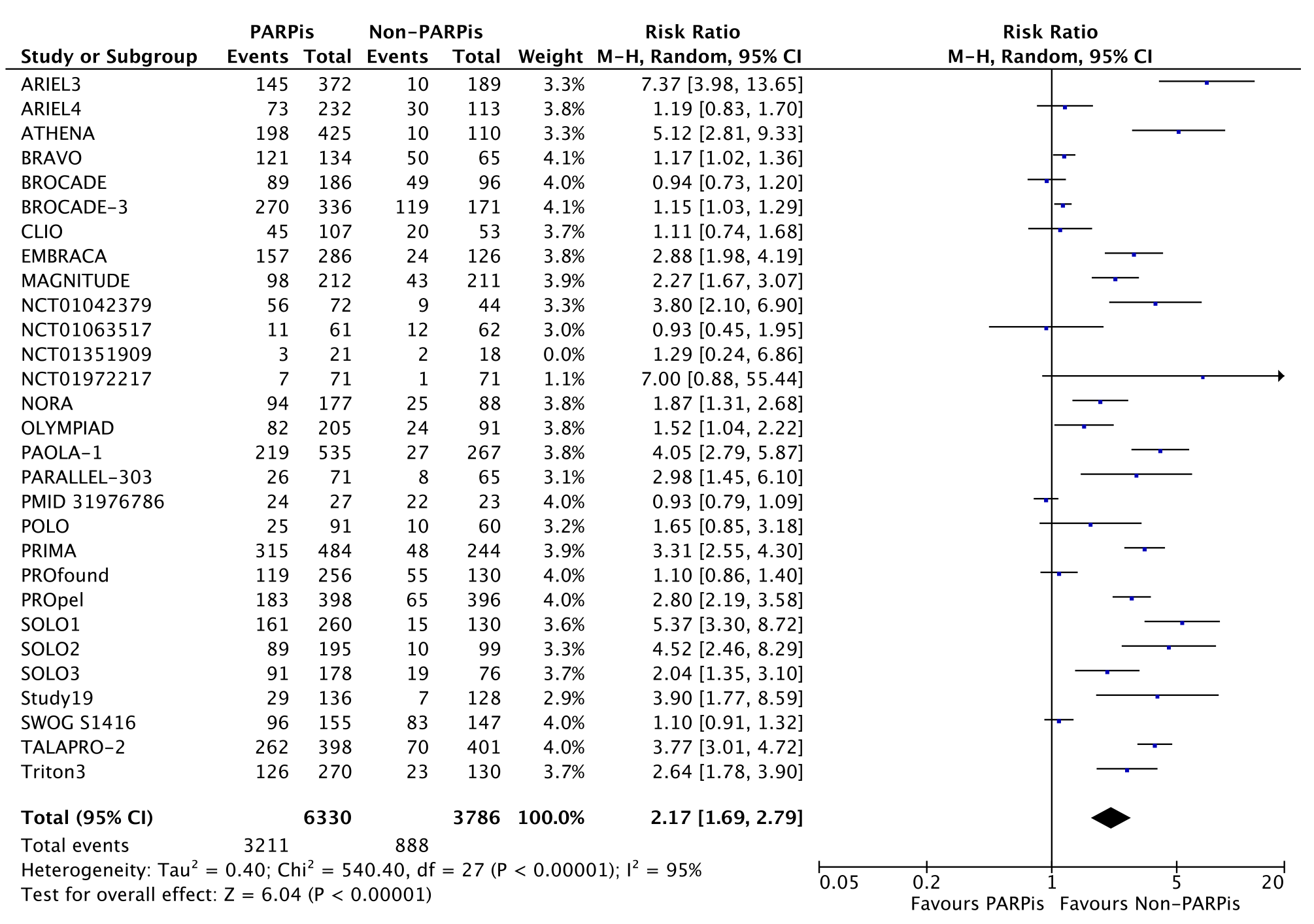 |
| 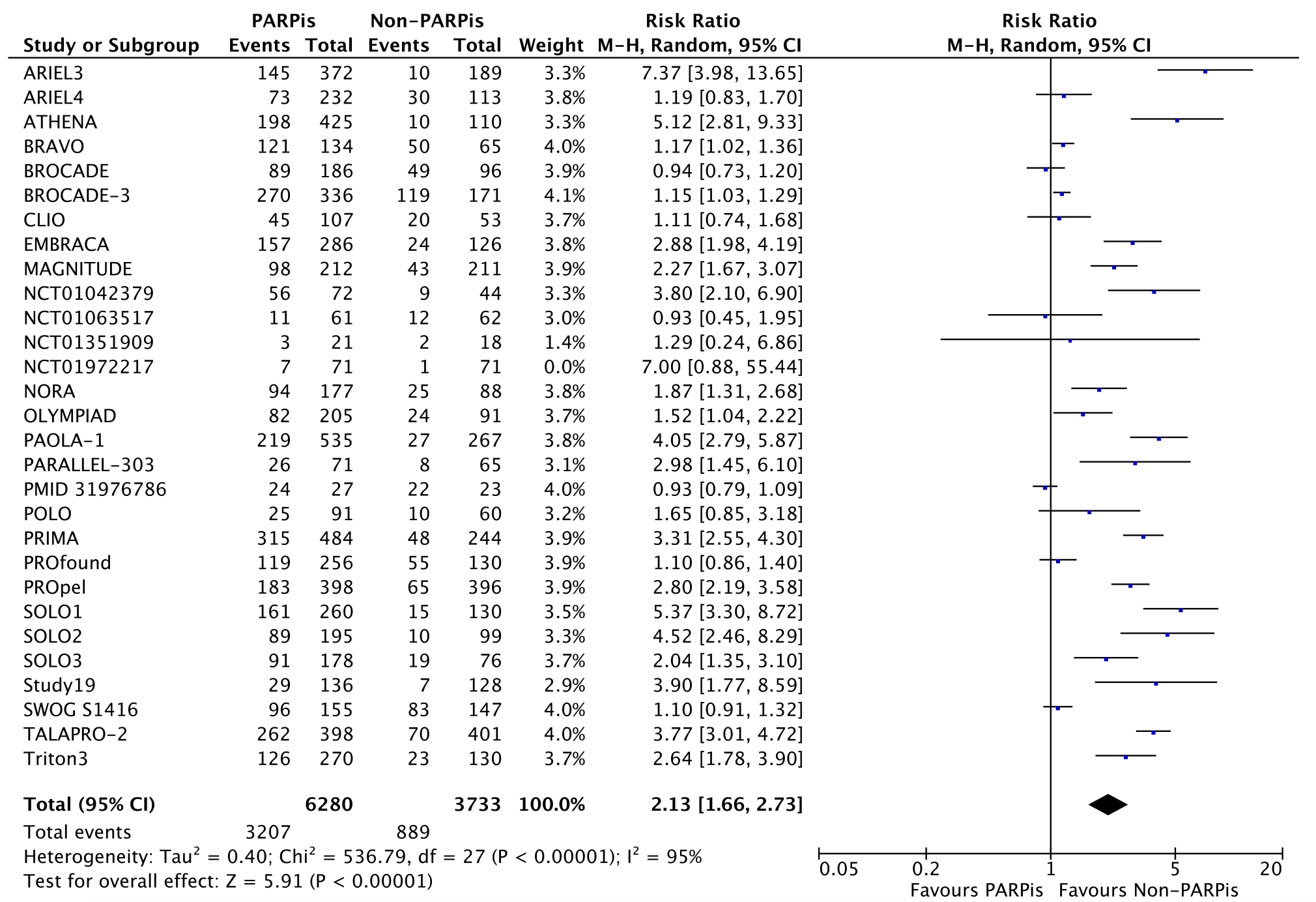 | 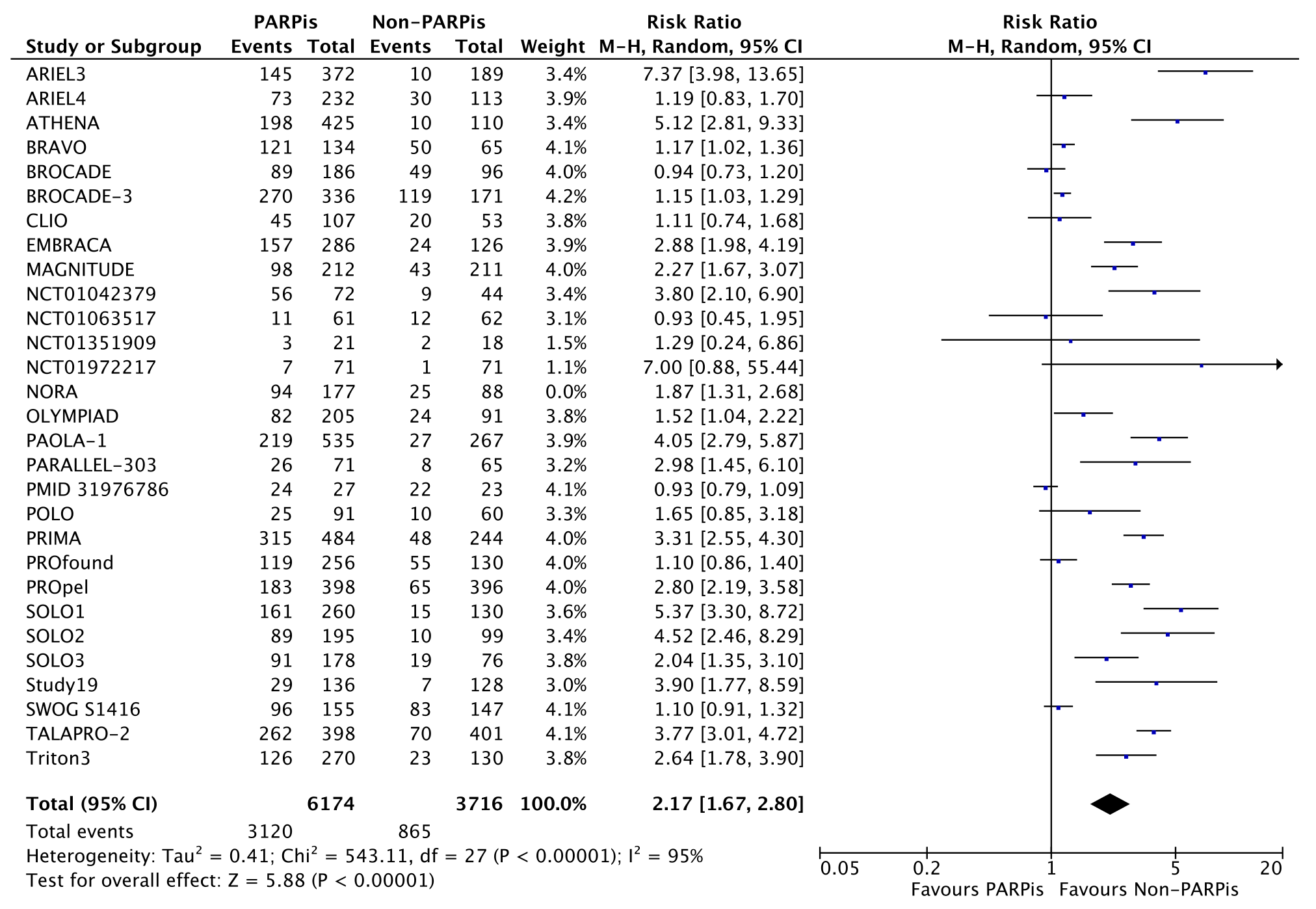 |
| 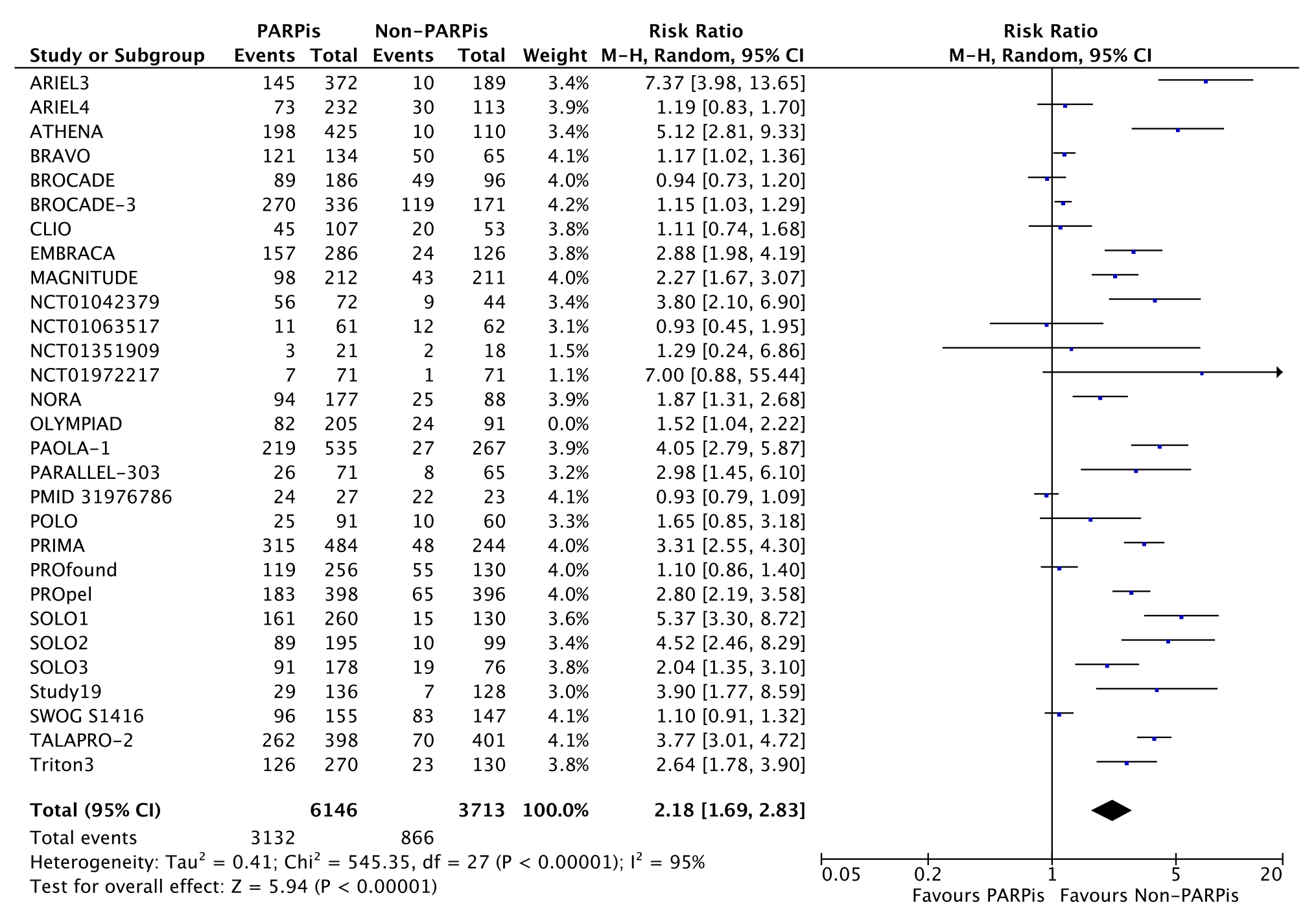 | 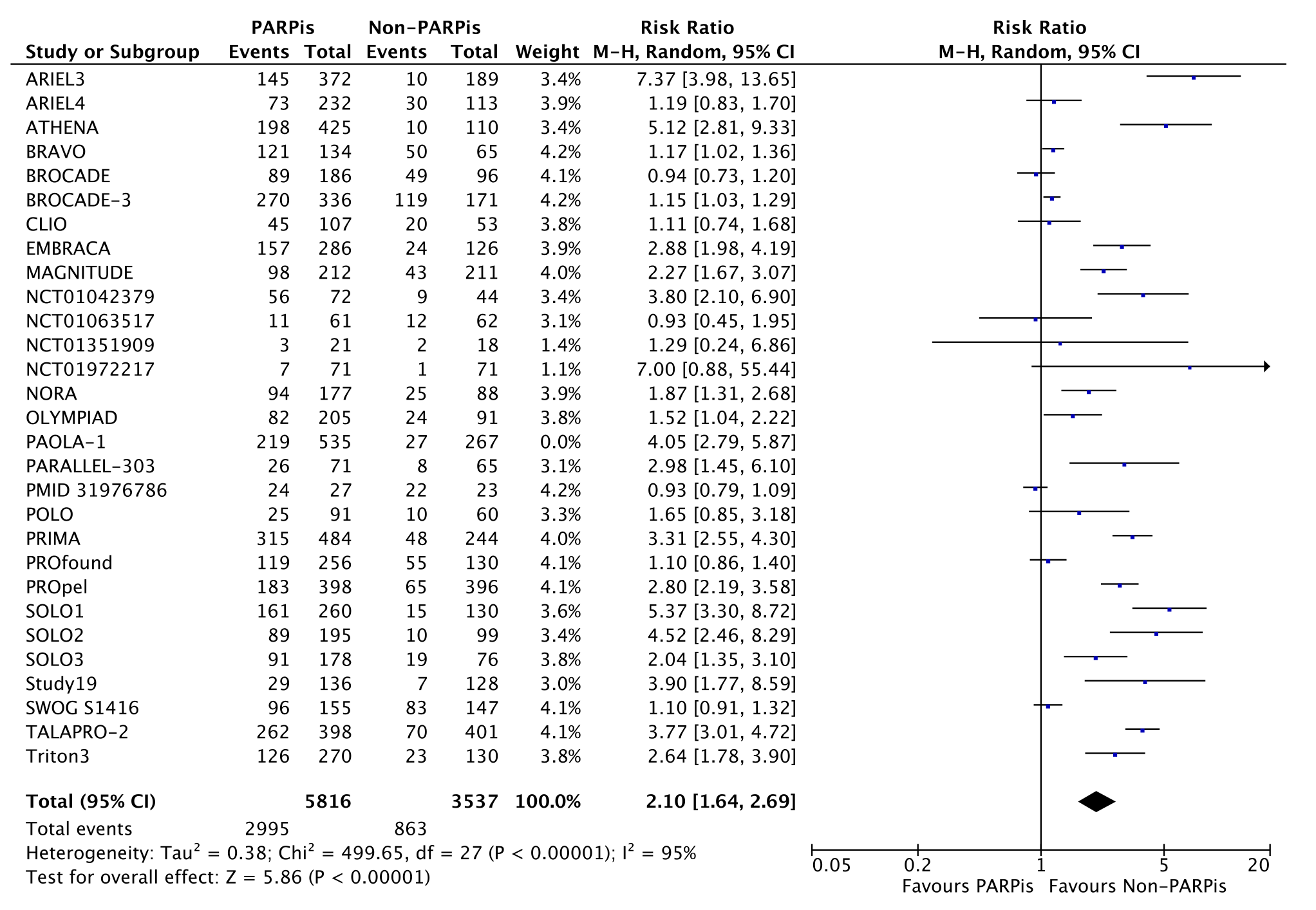 |
| 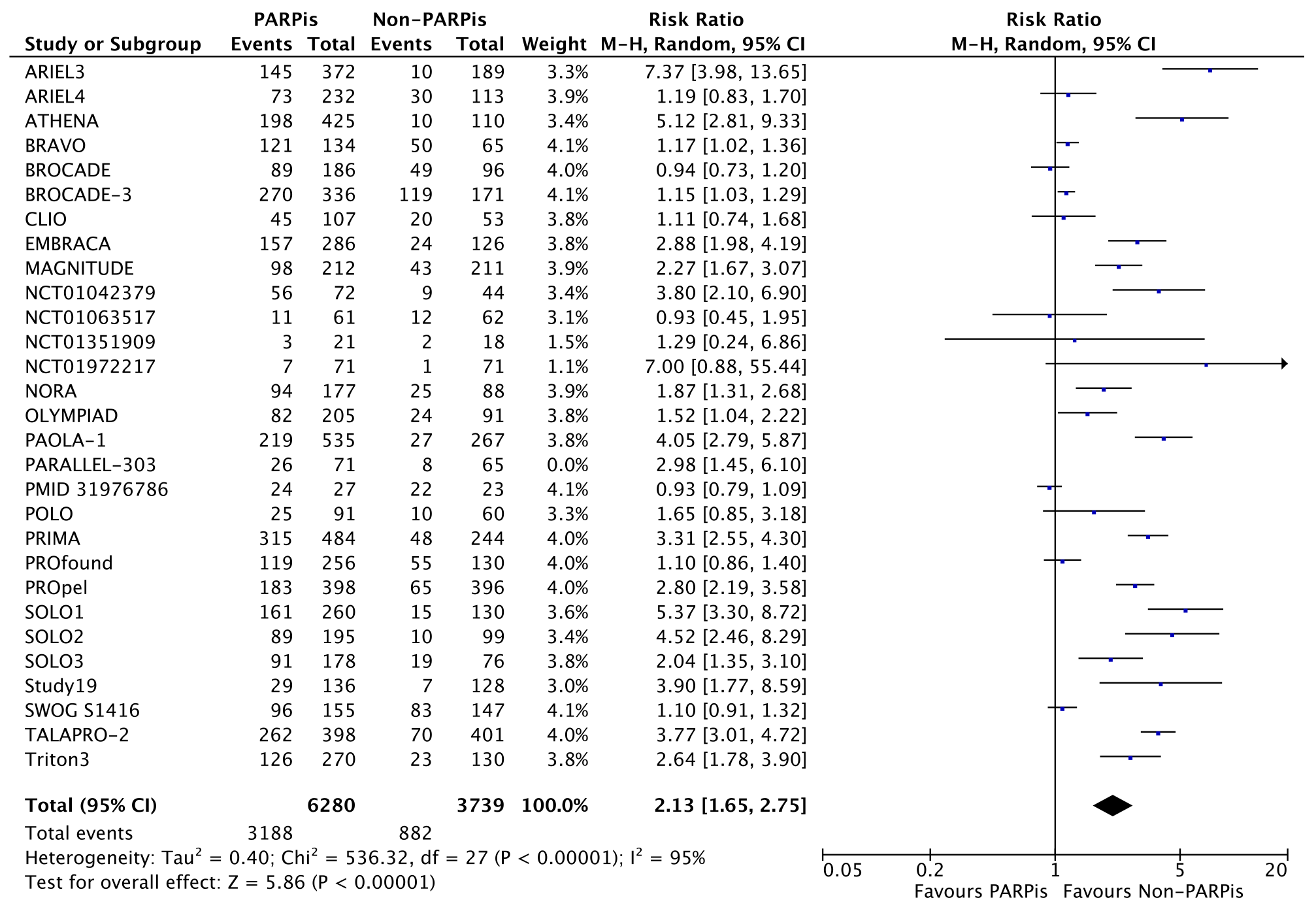 | 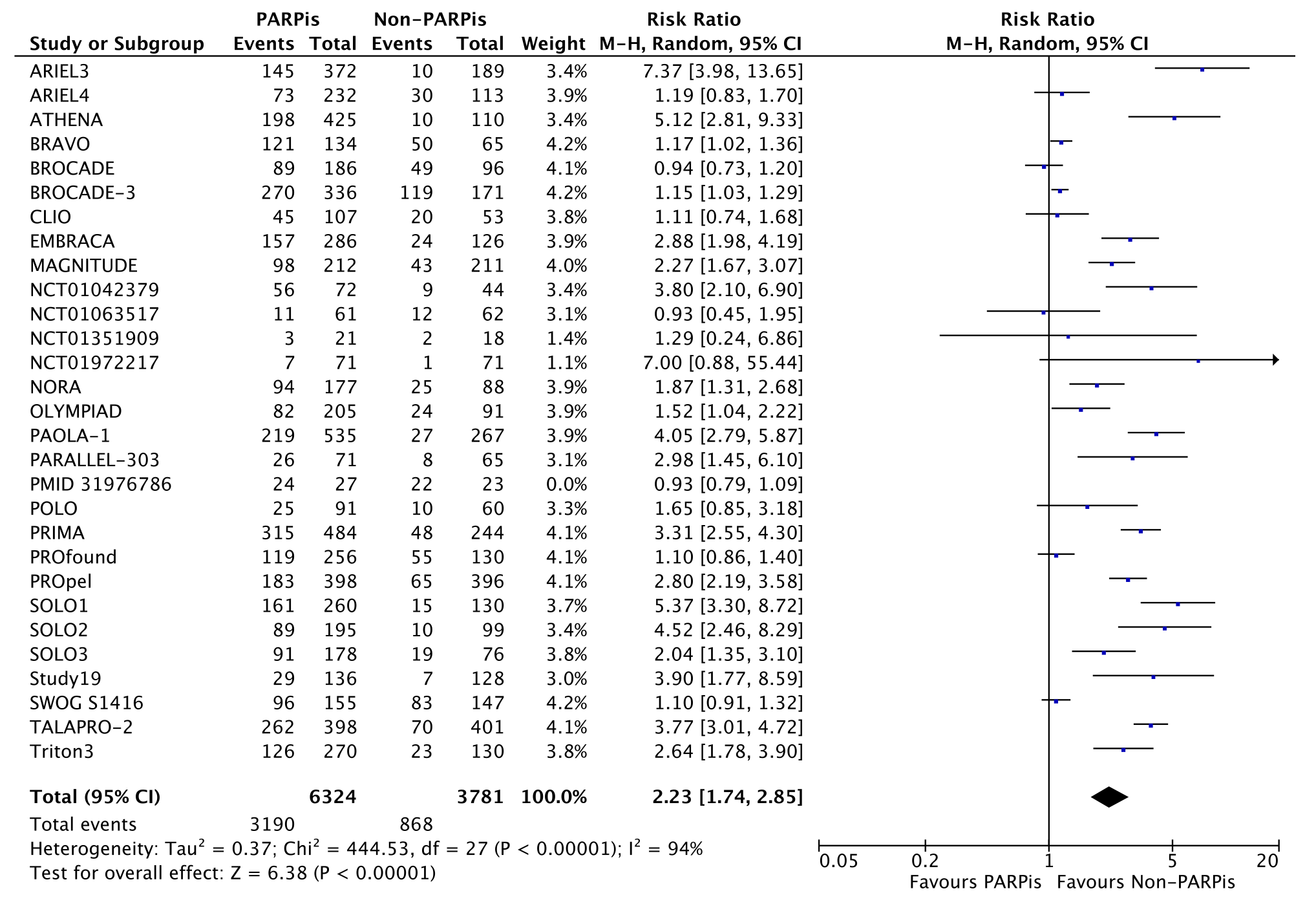 |
| 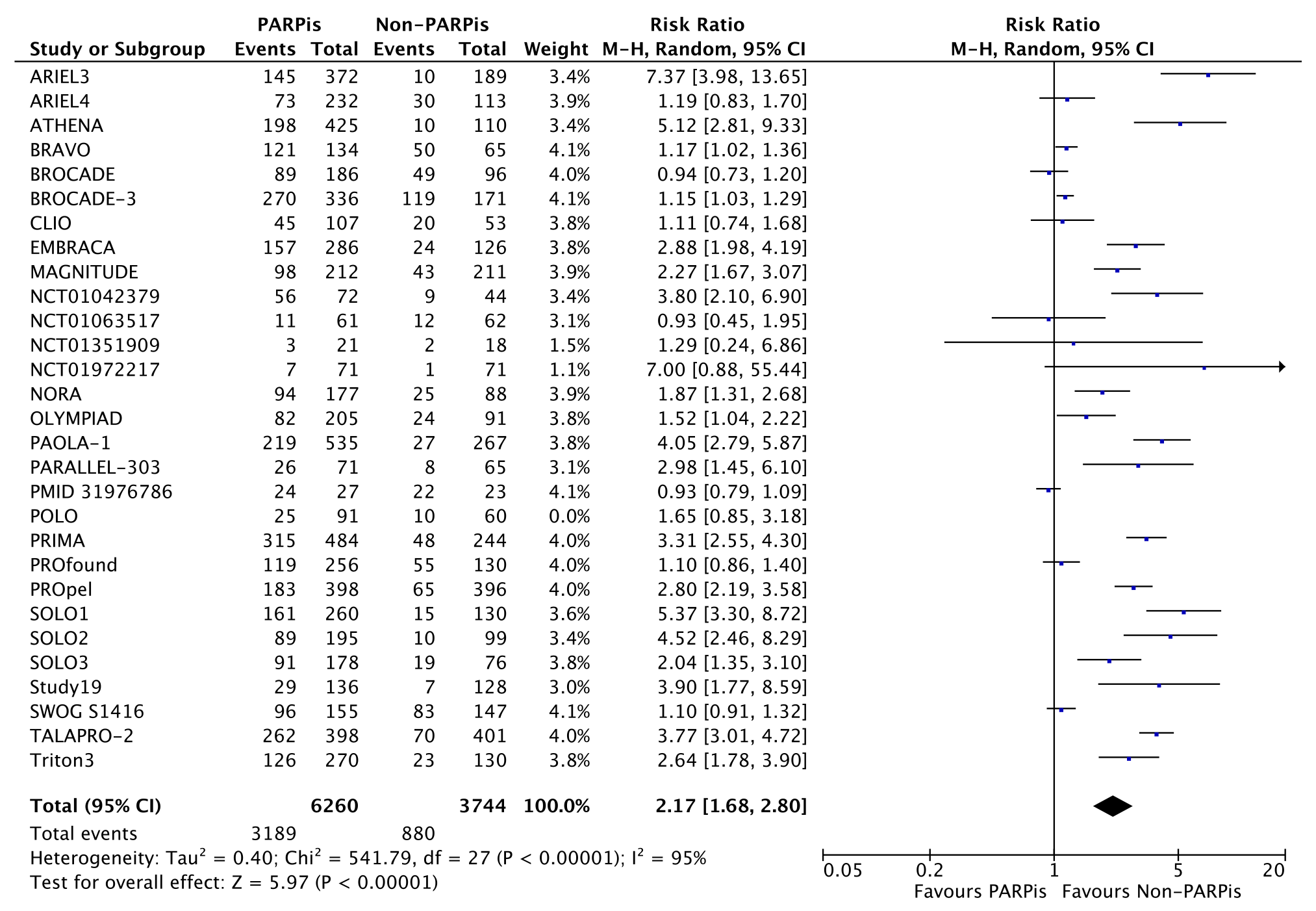 | 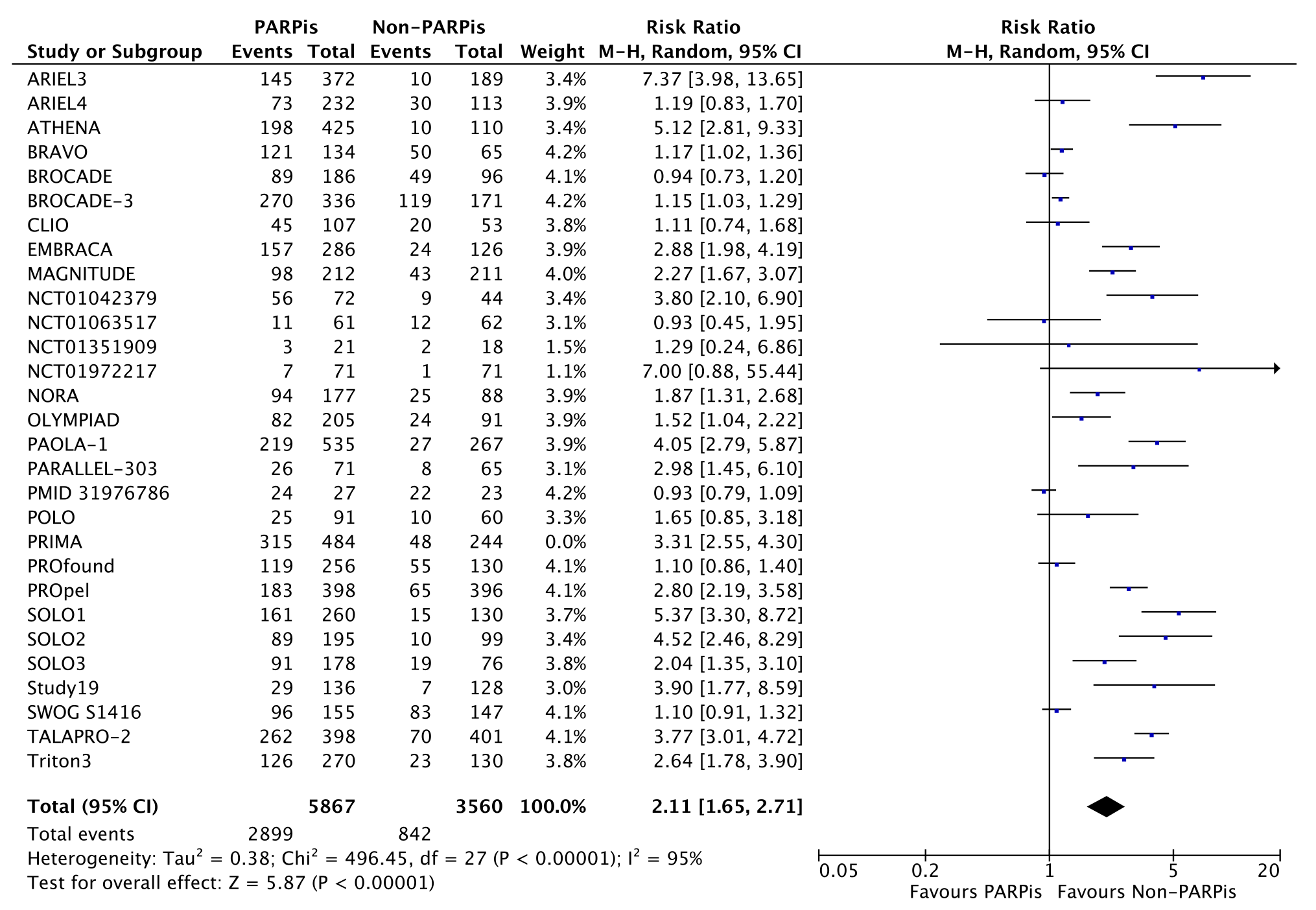 |
| 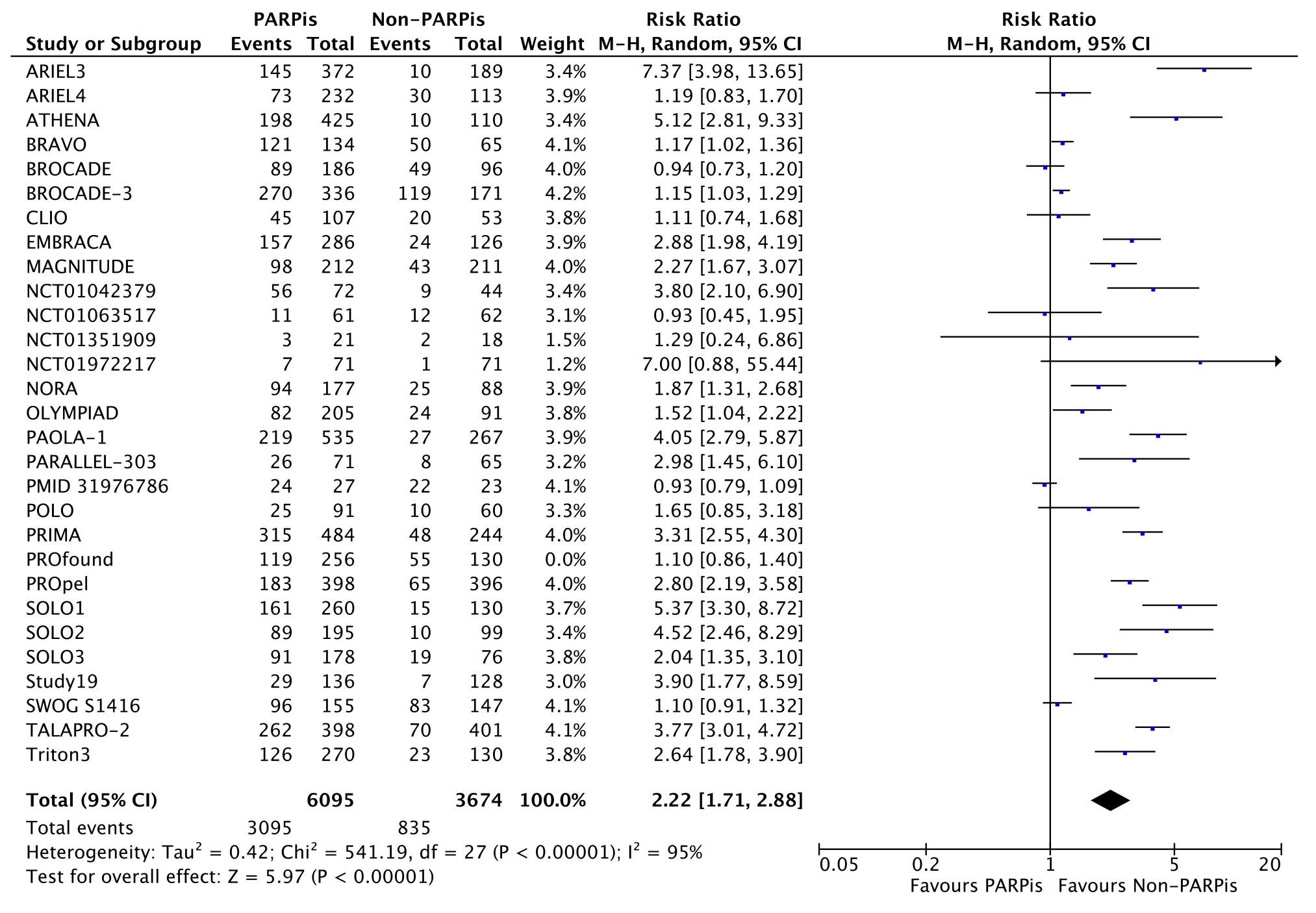 | 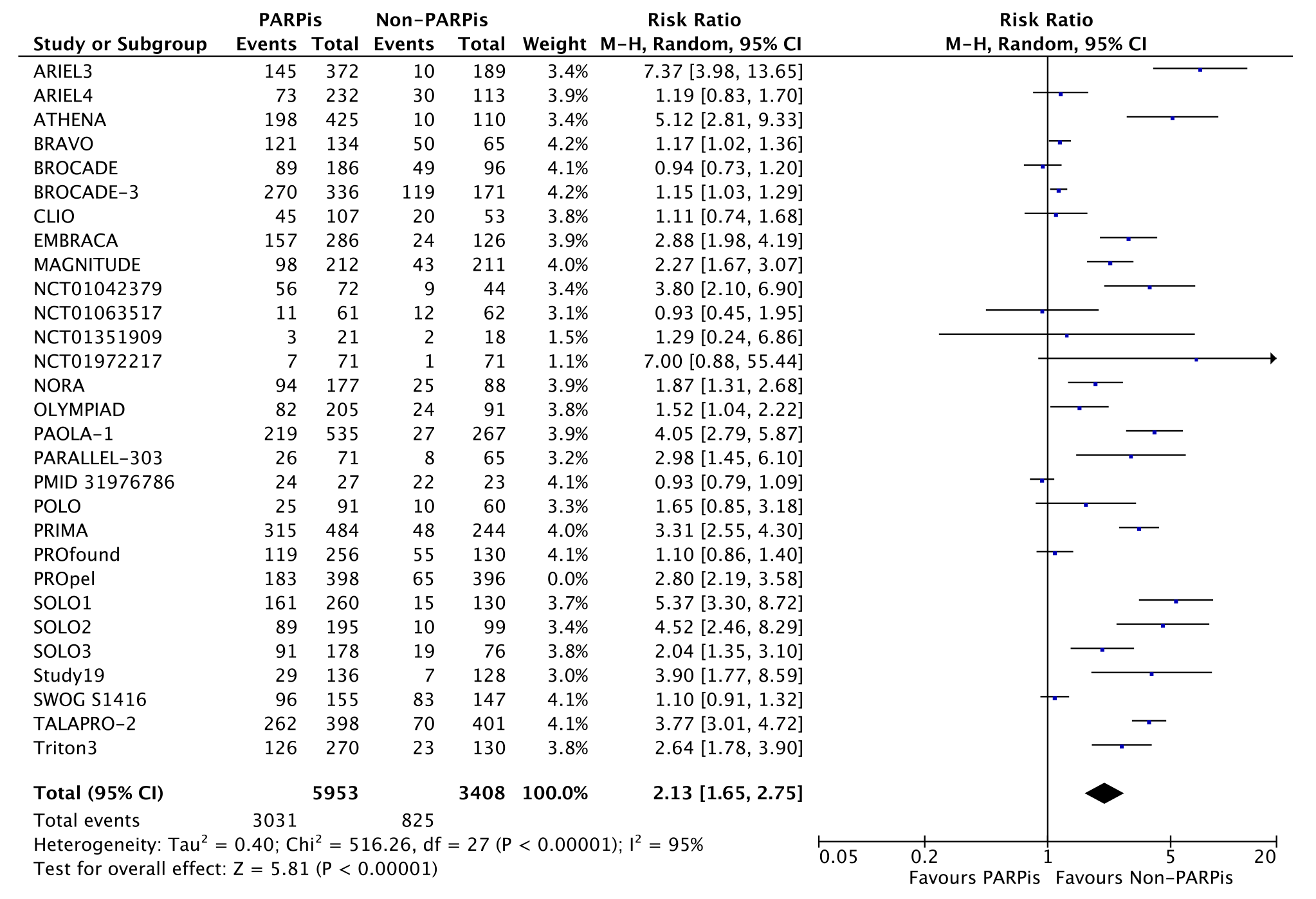 |
| 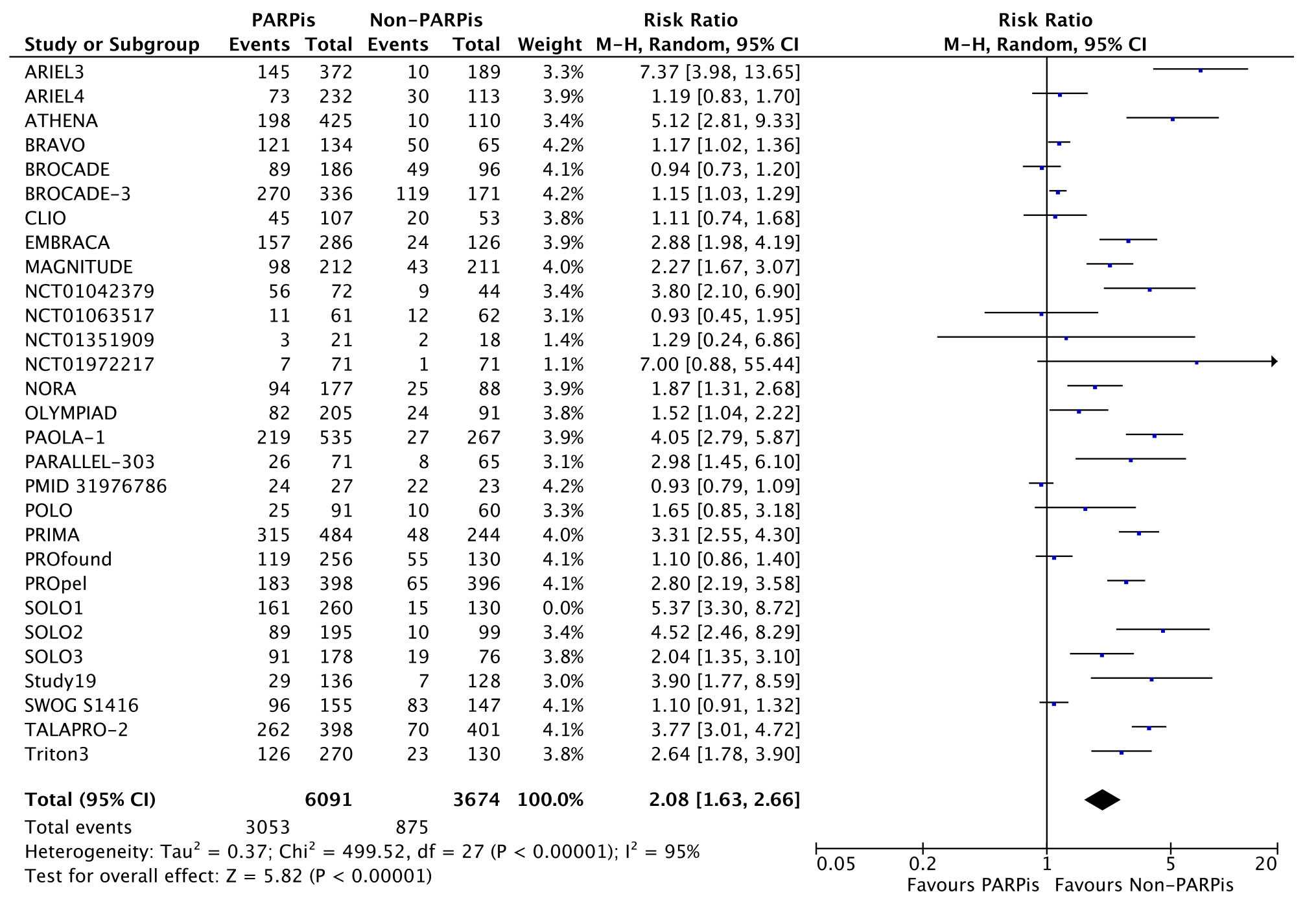 | 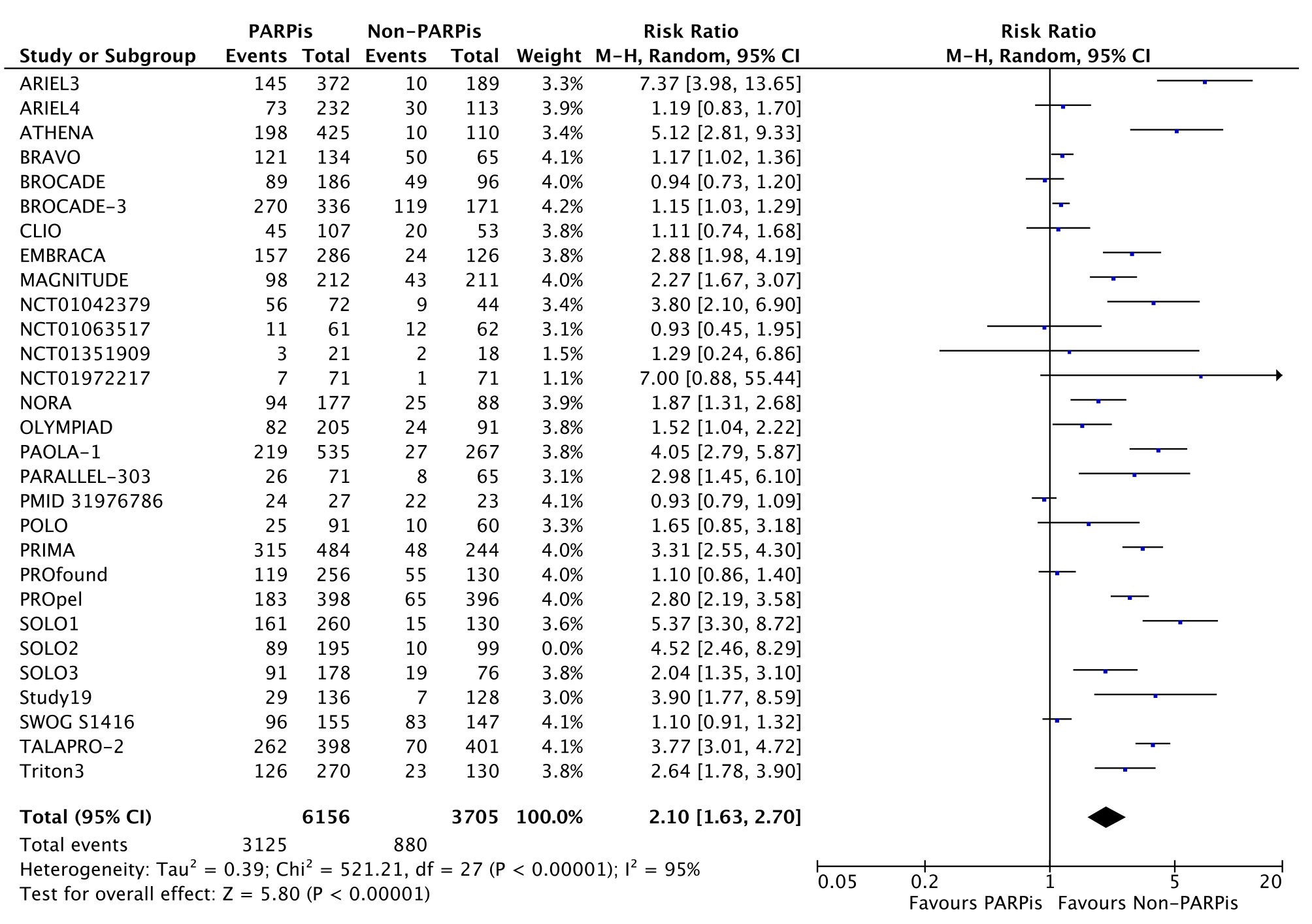 |
| 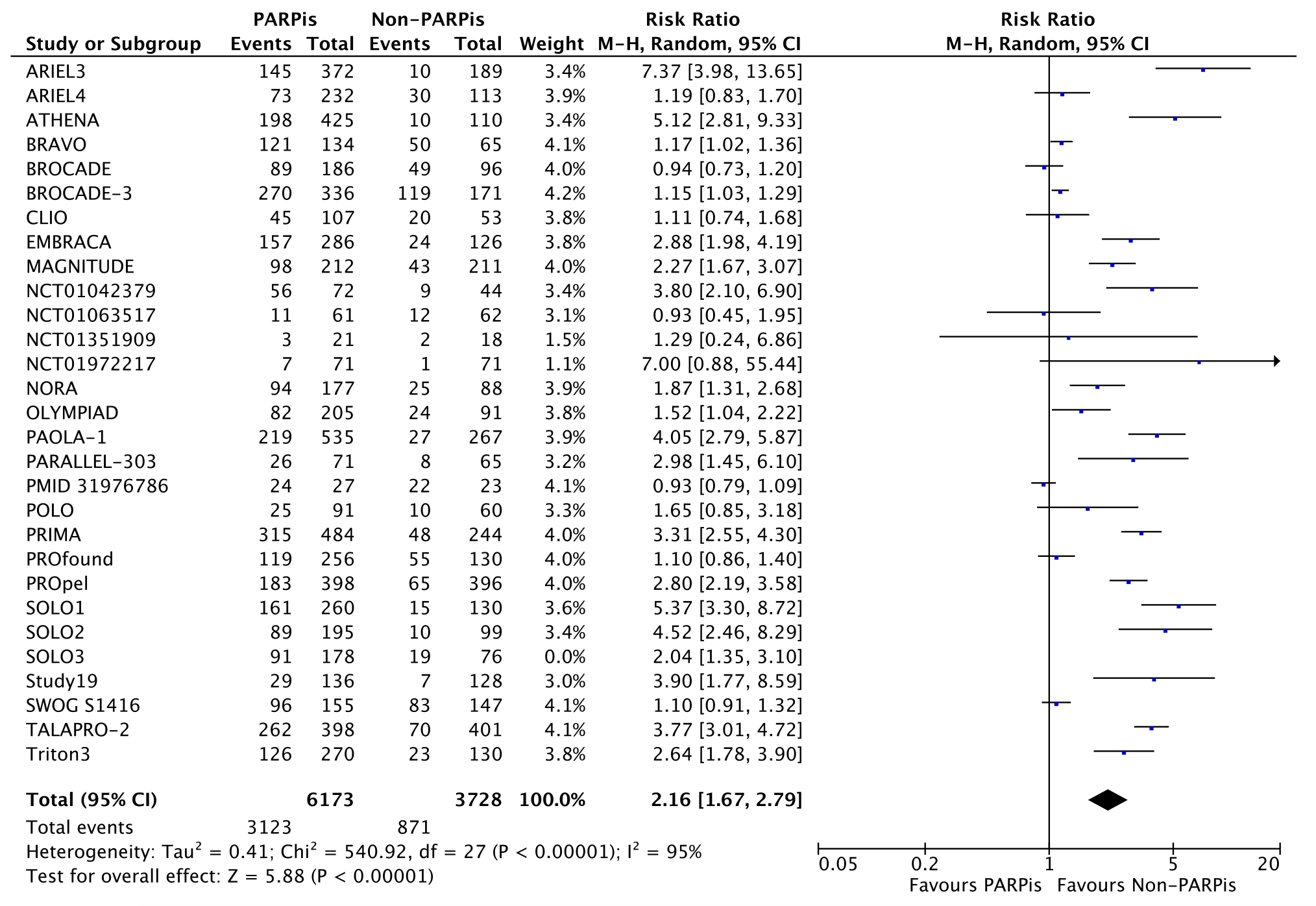 | 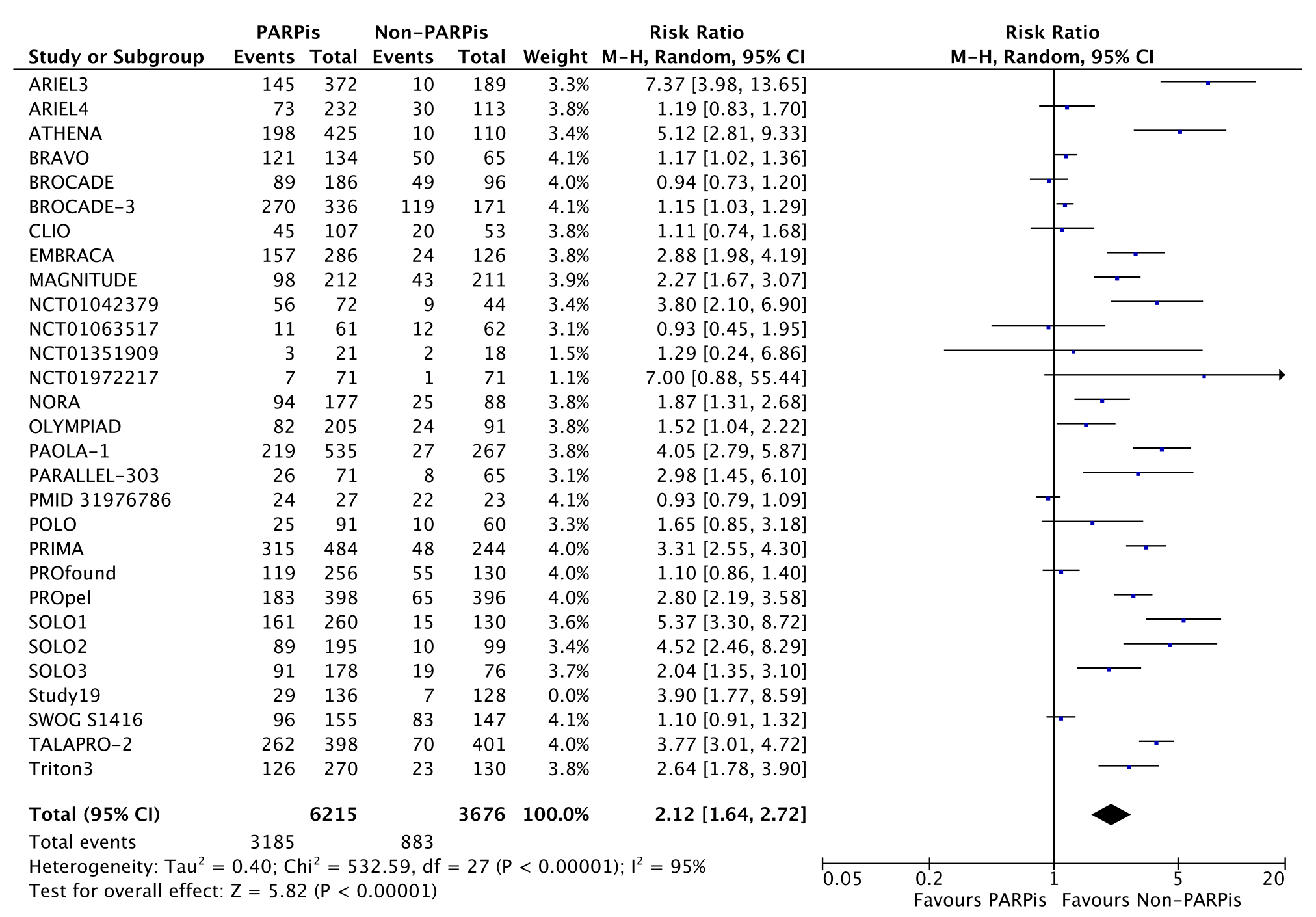 |
| 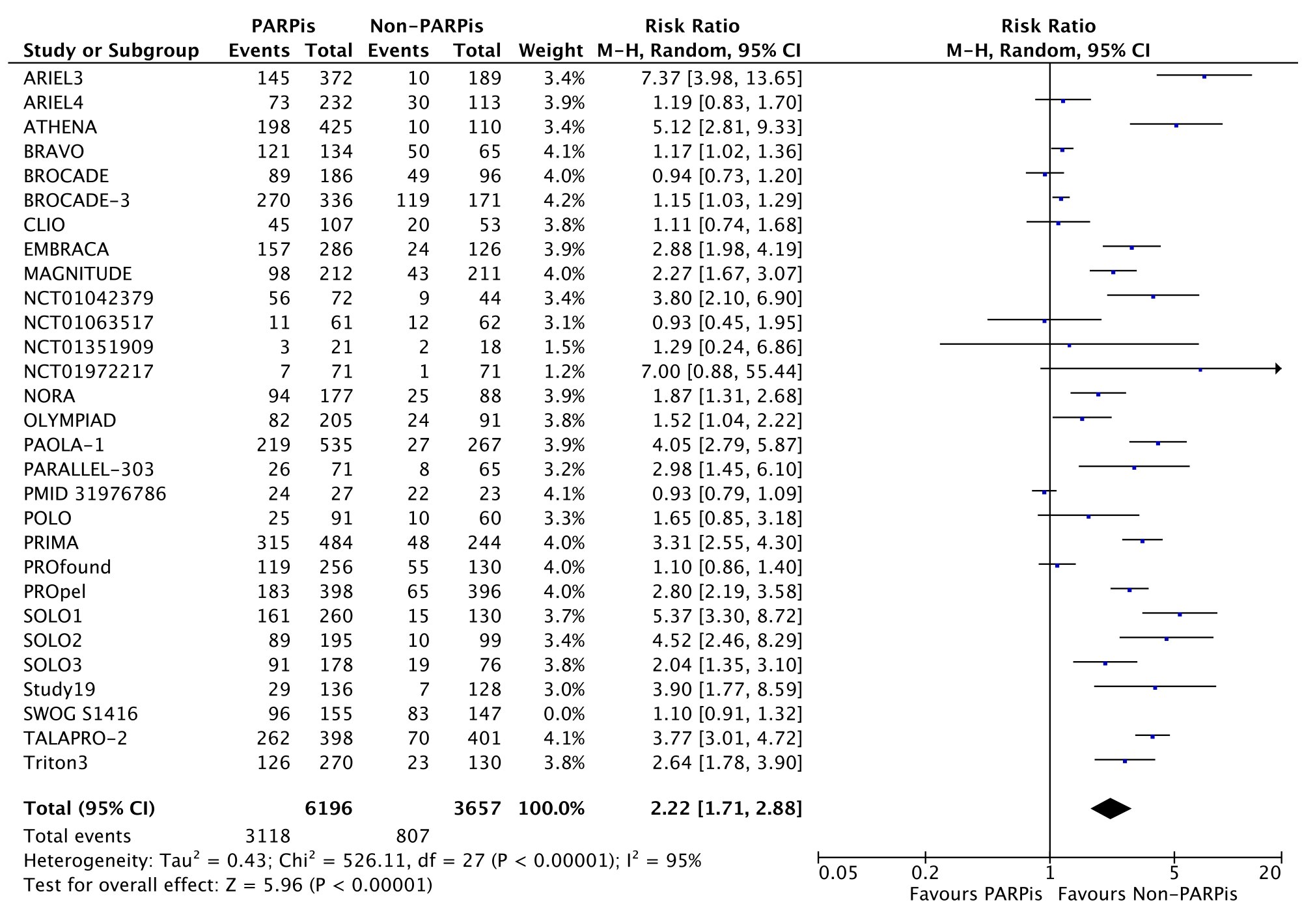 | 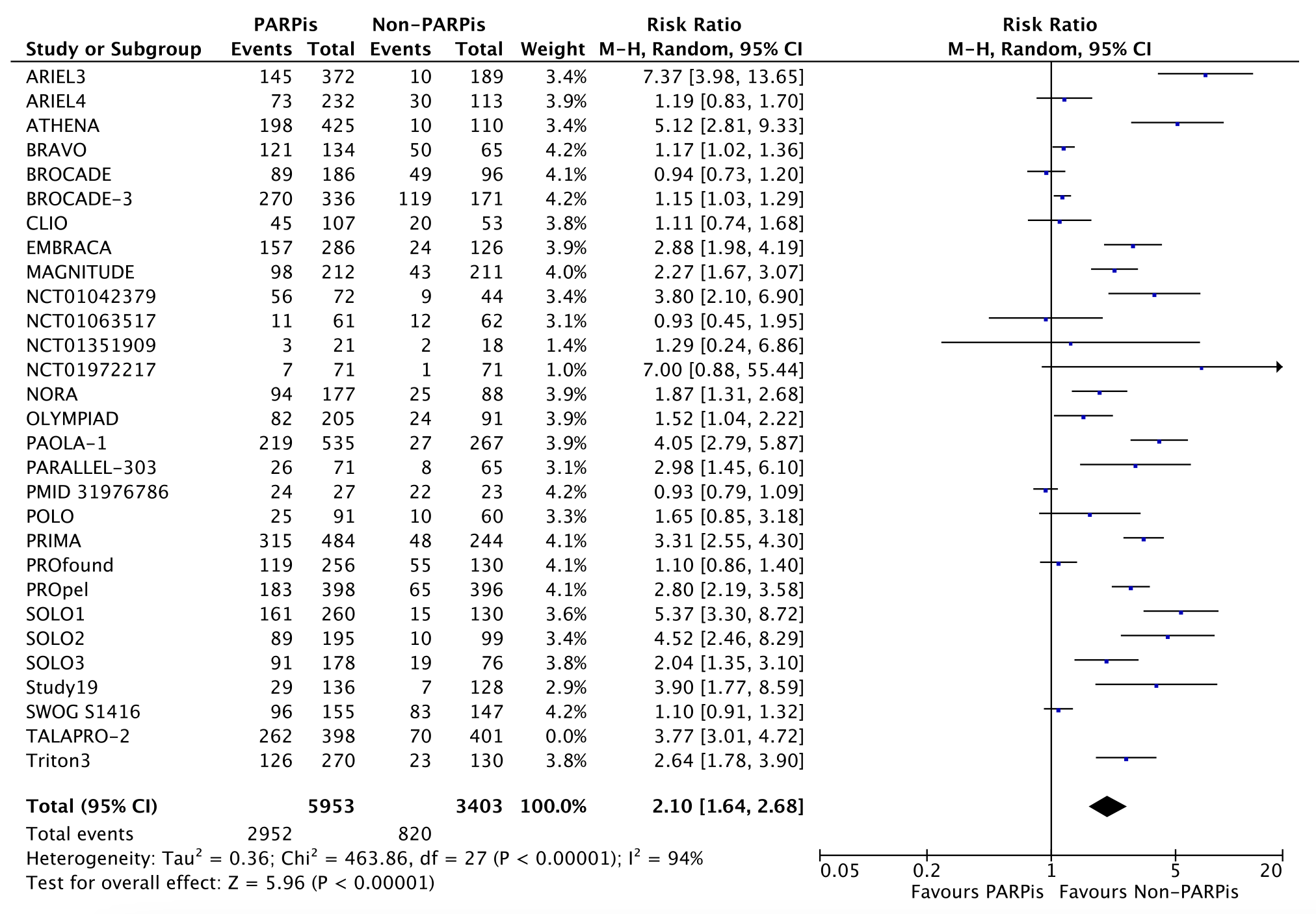 |

B

| 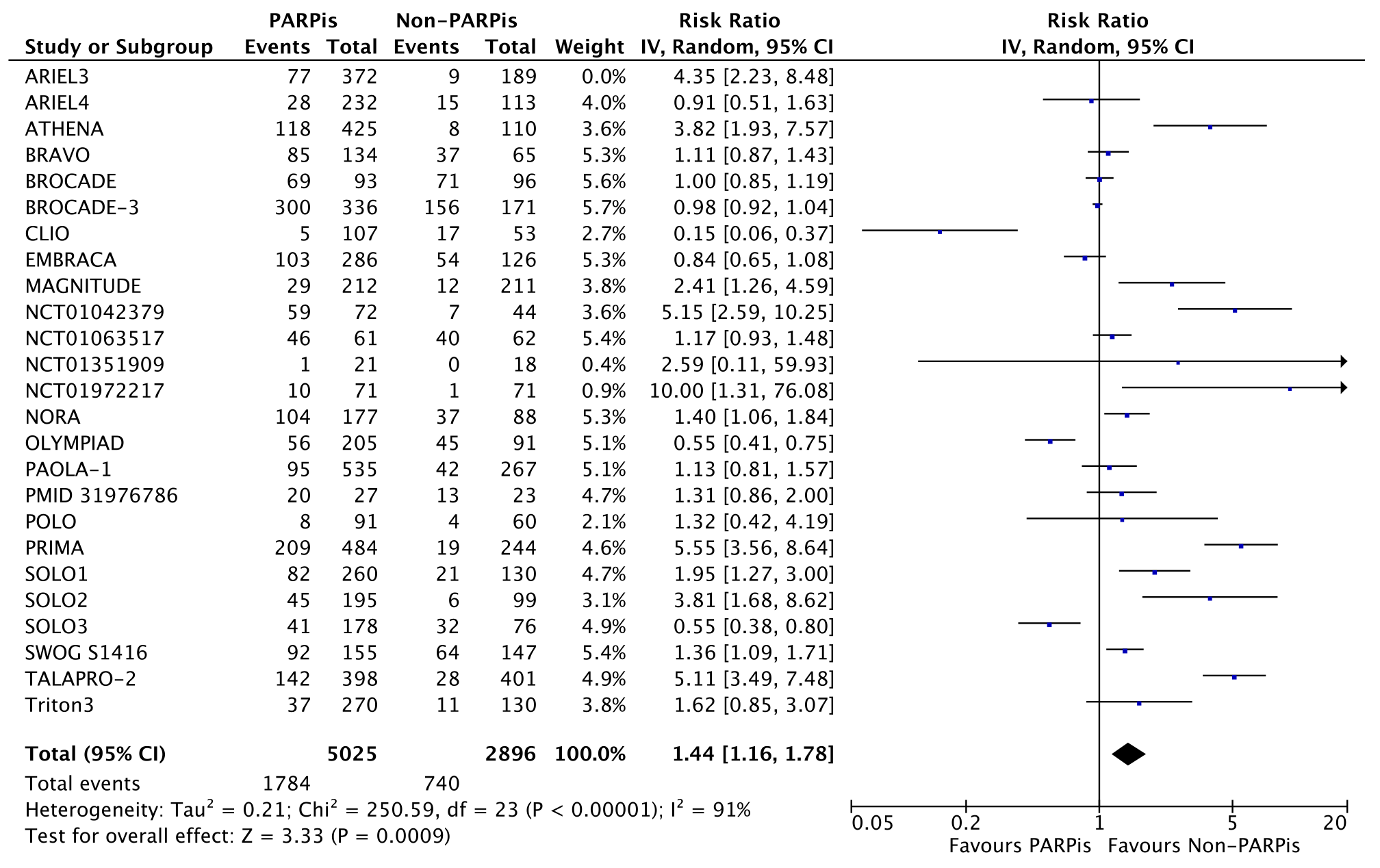 | 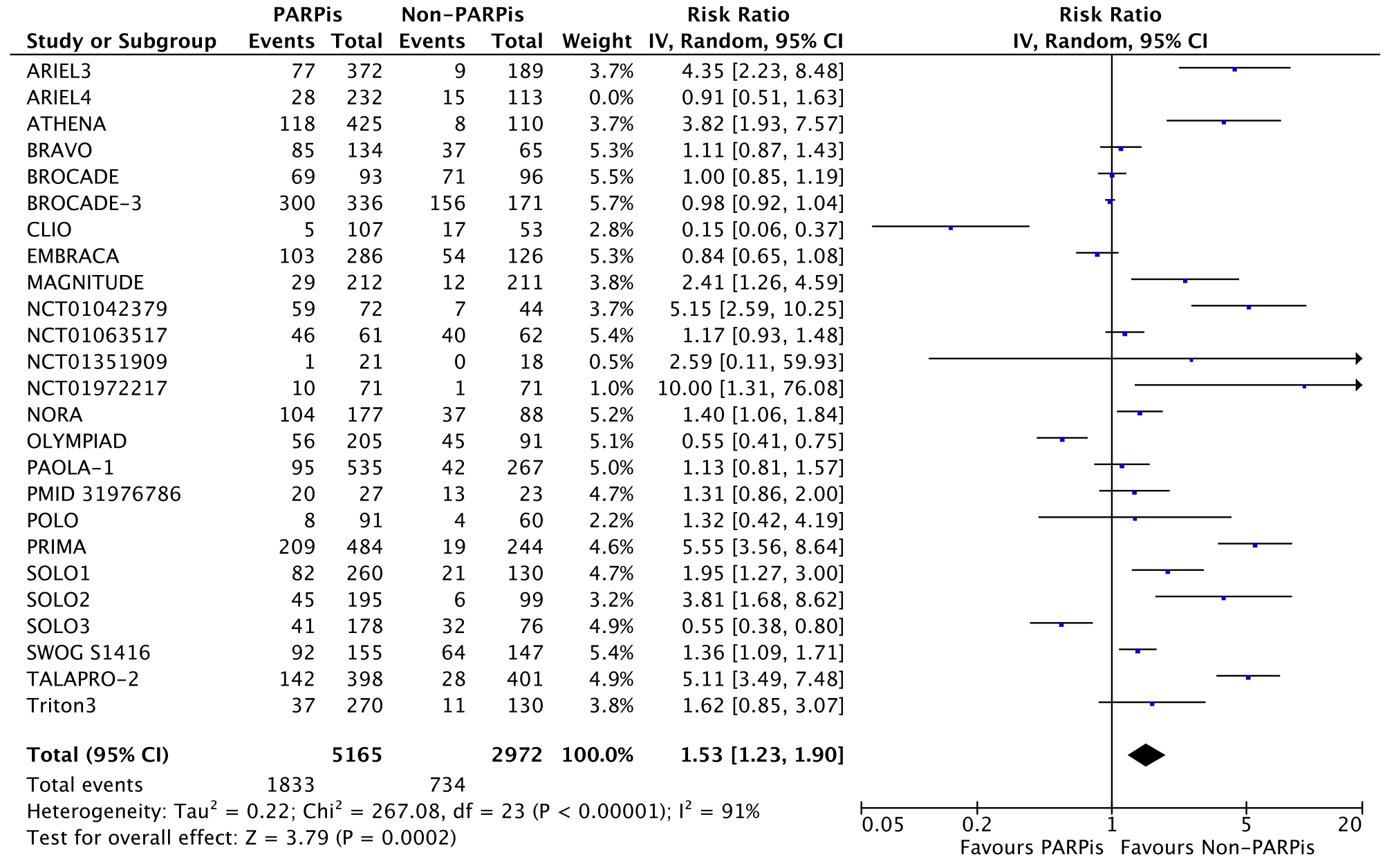 |
| --- | --- |
| 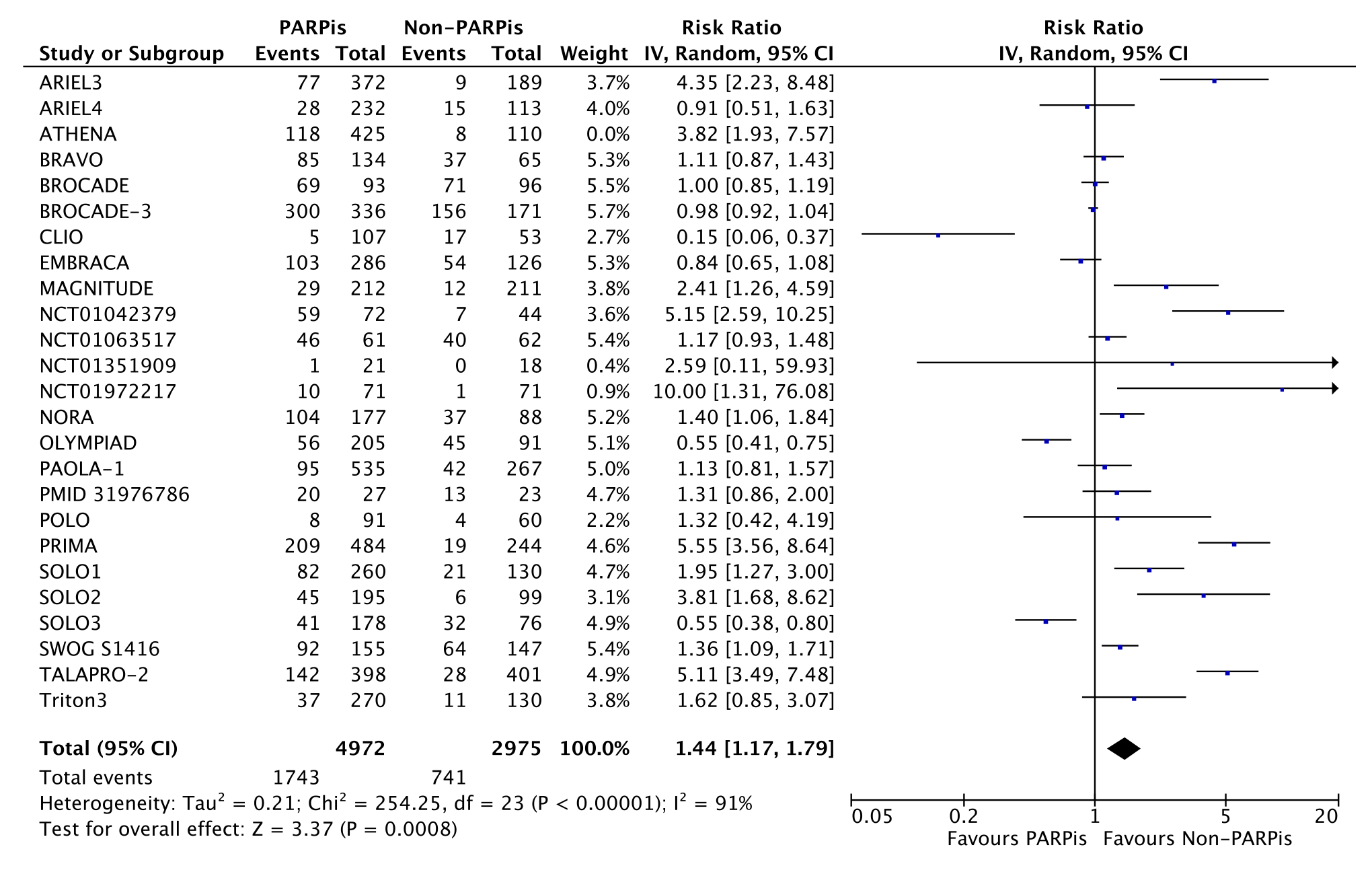 | 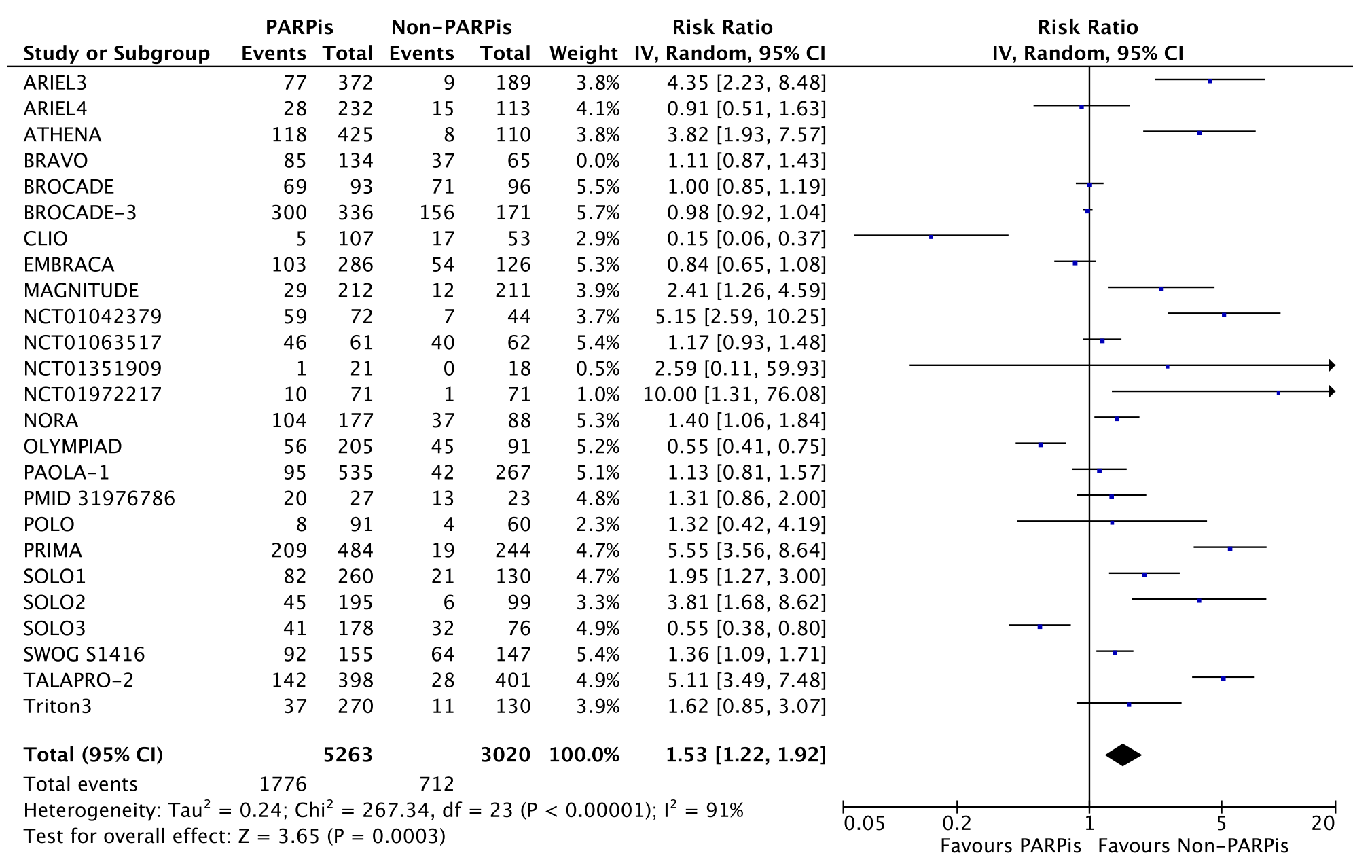 |
| 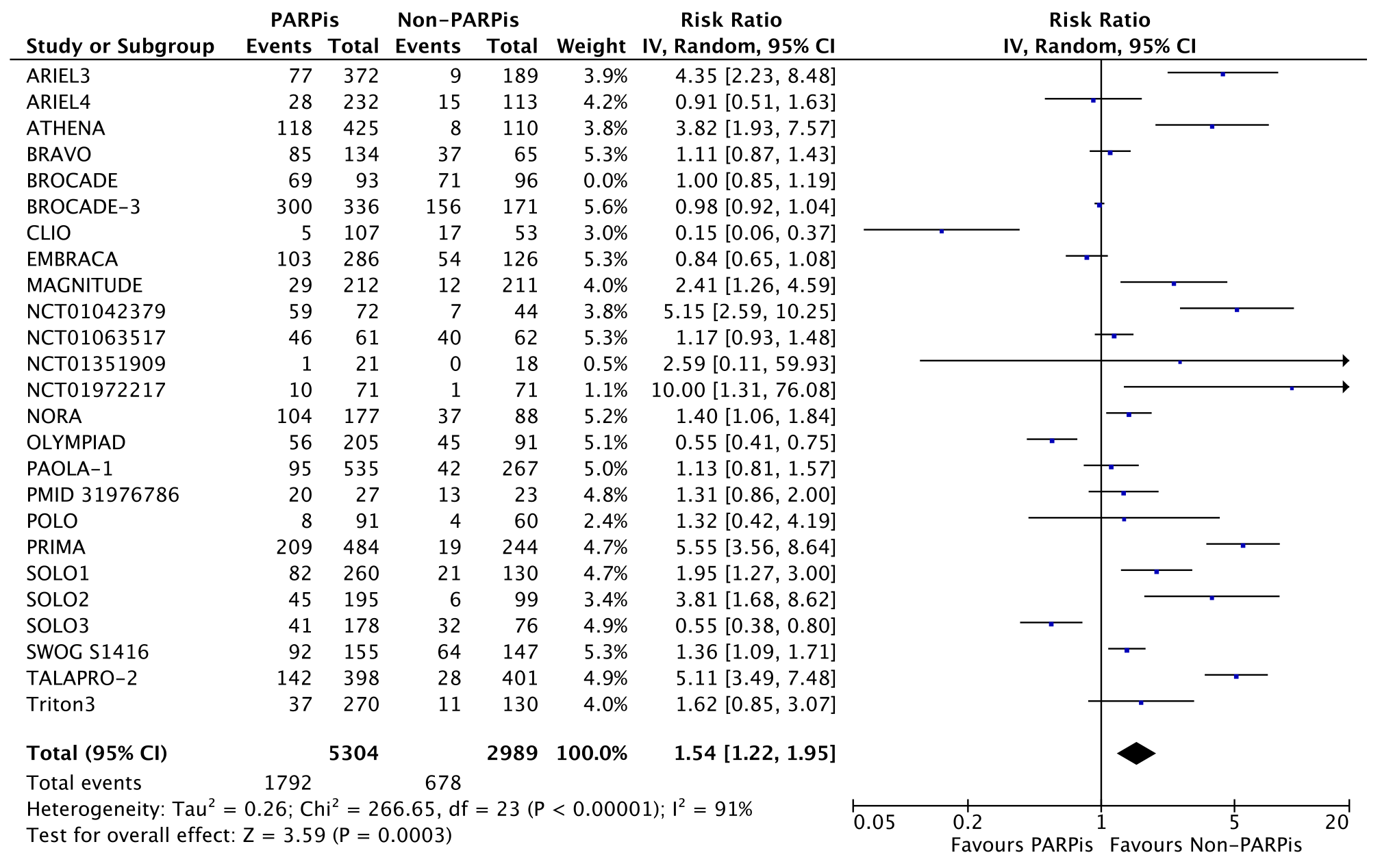 | 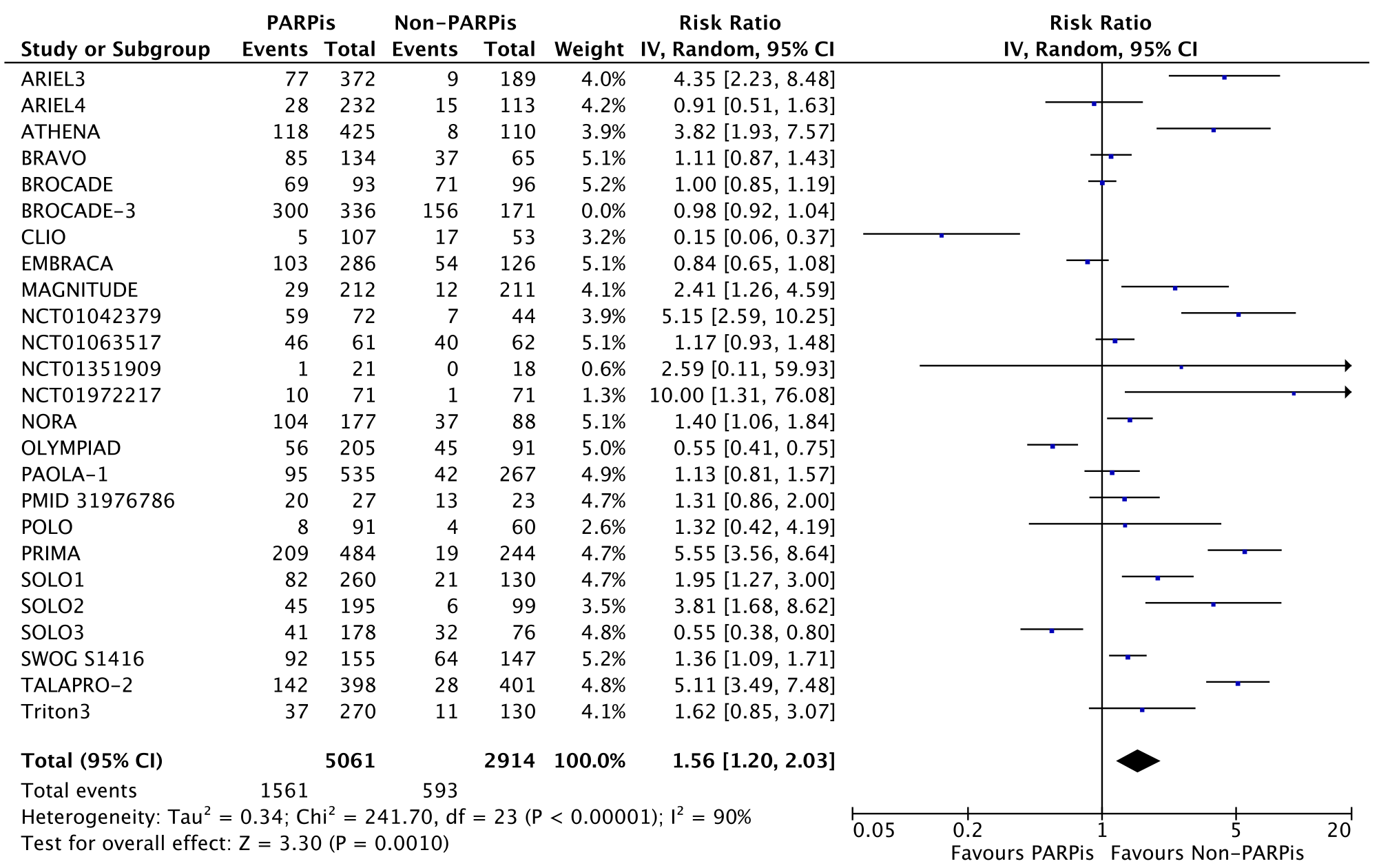 |
| 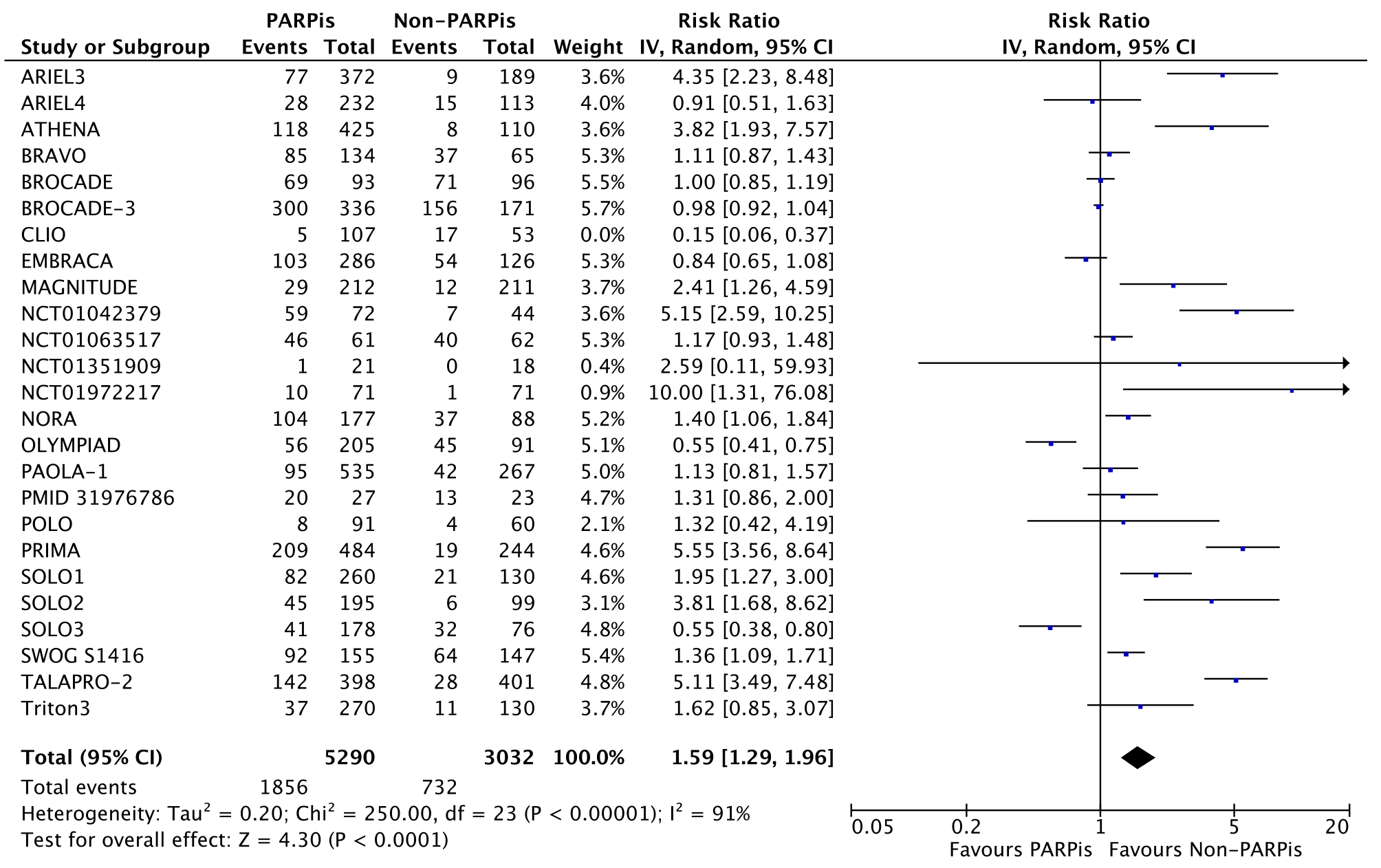 | 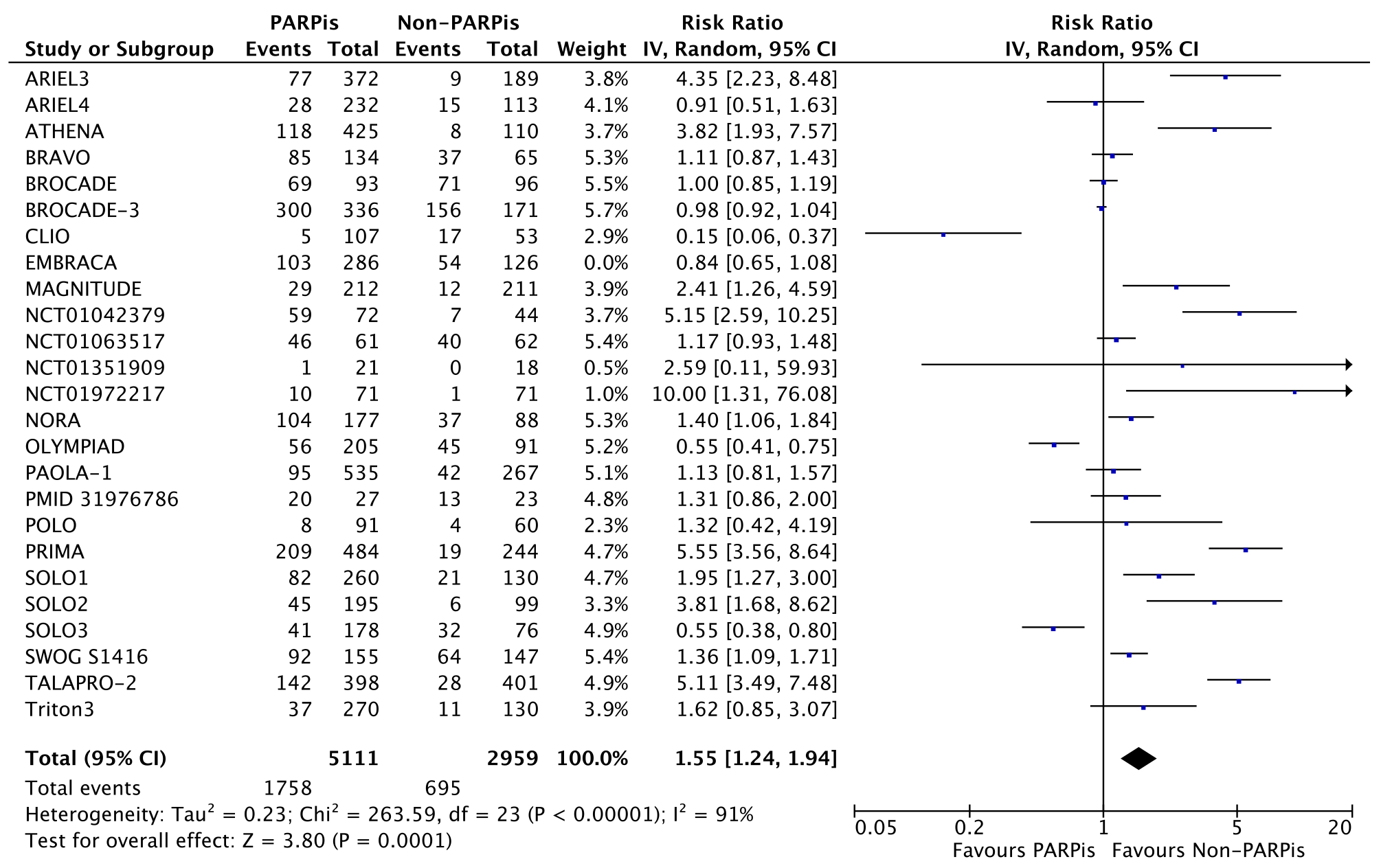 |
| 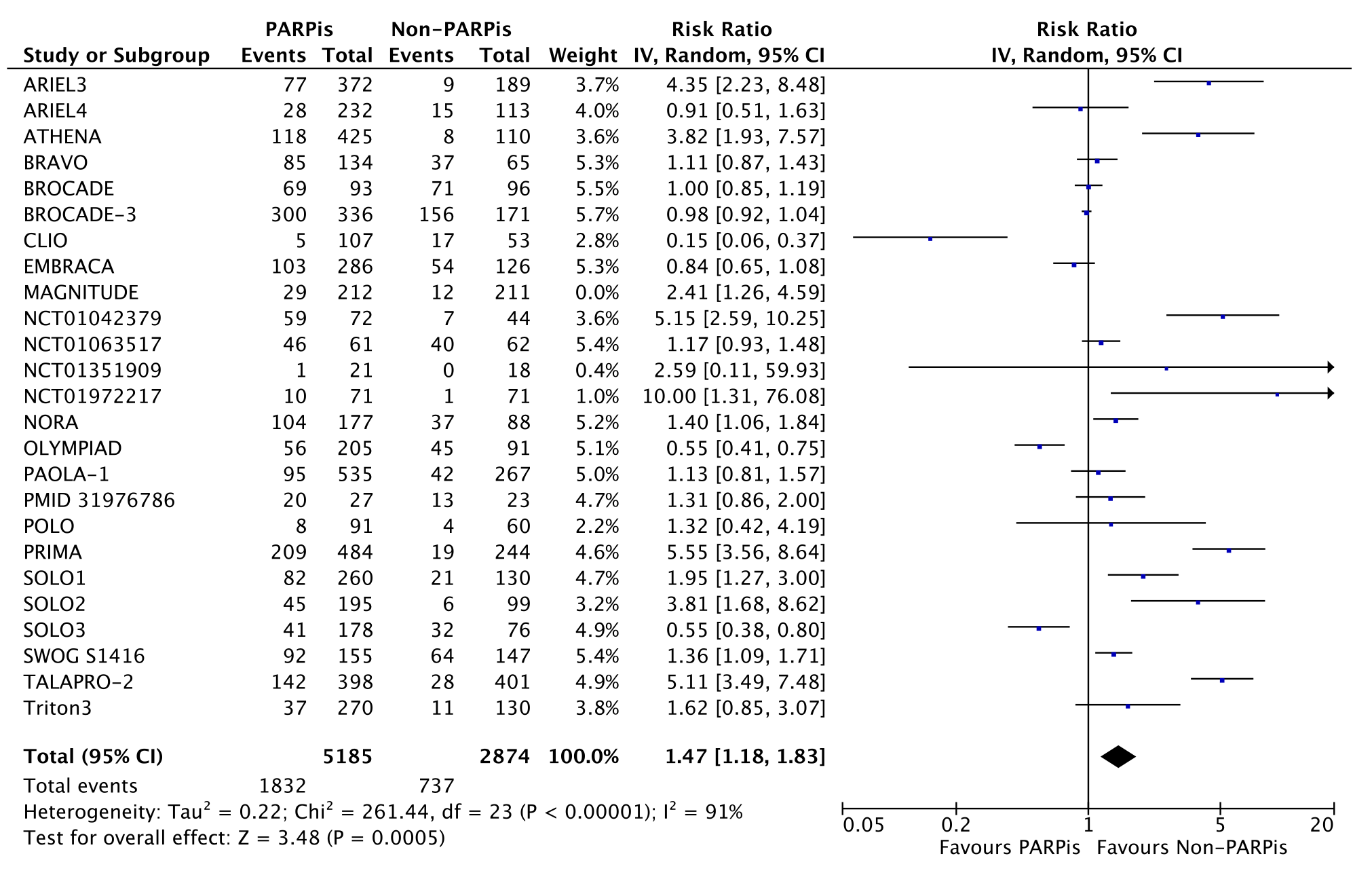 | 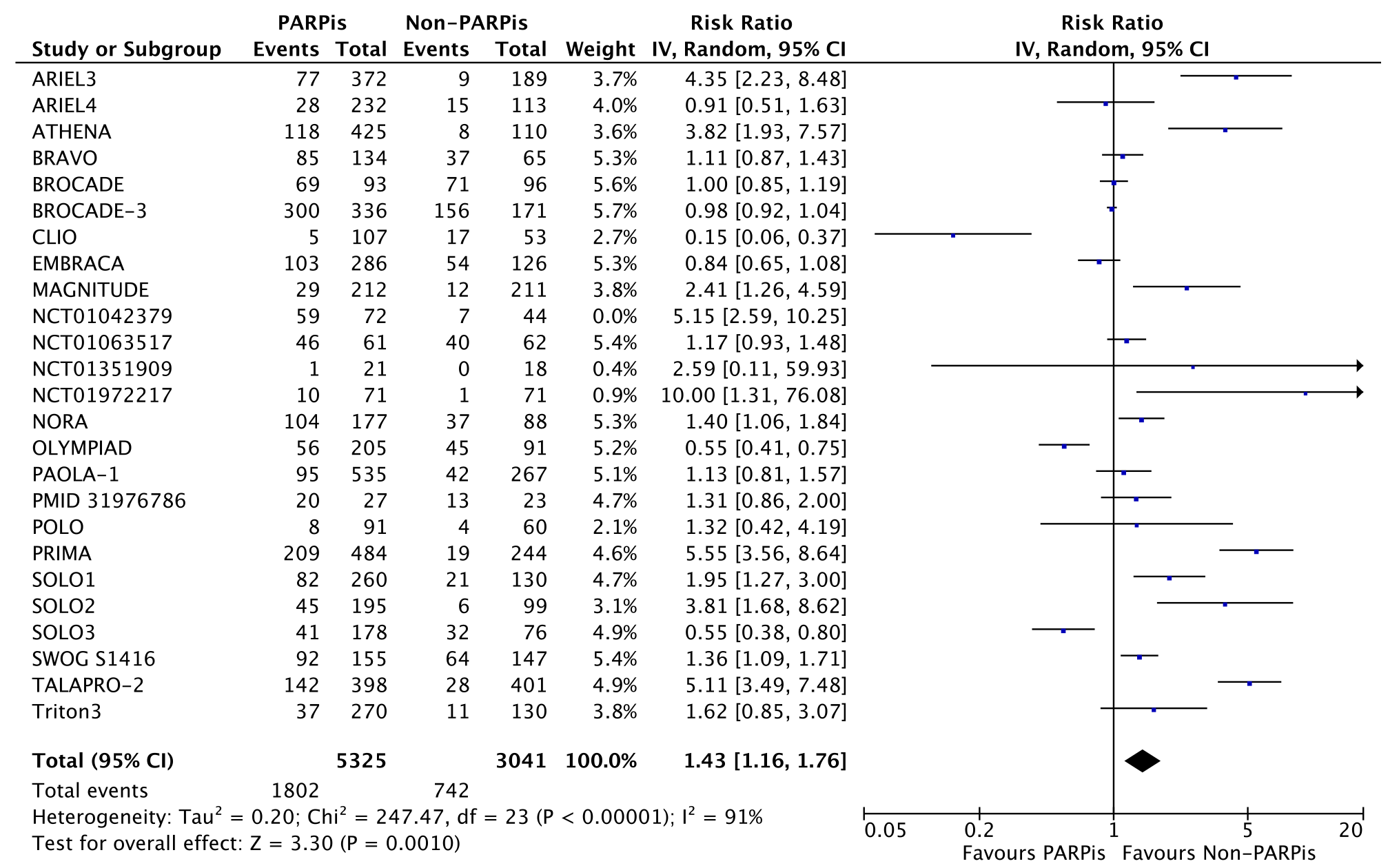 |
| 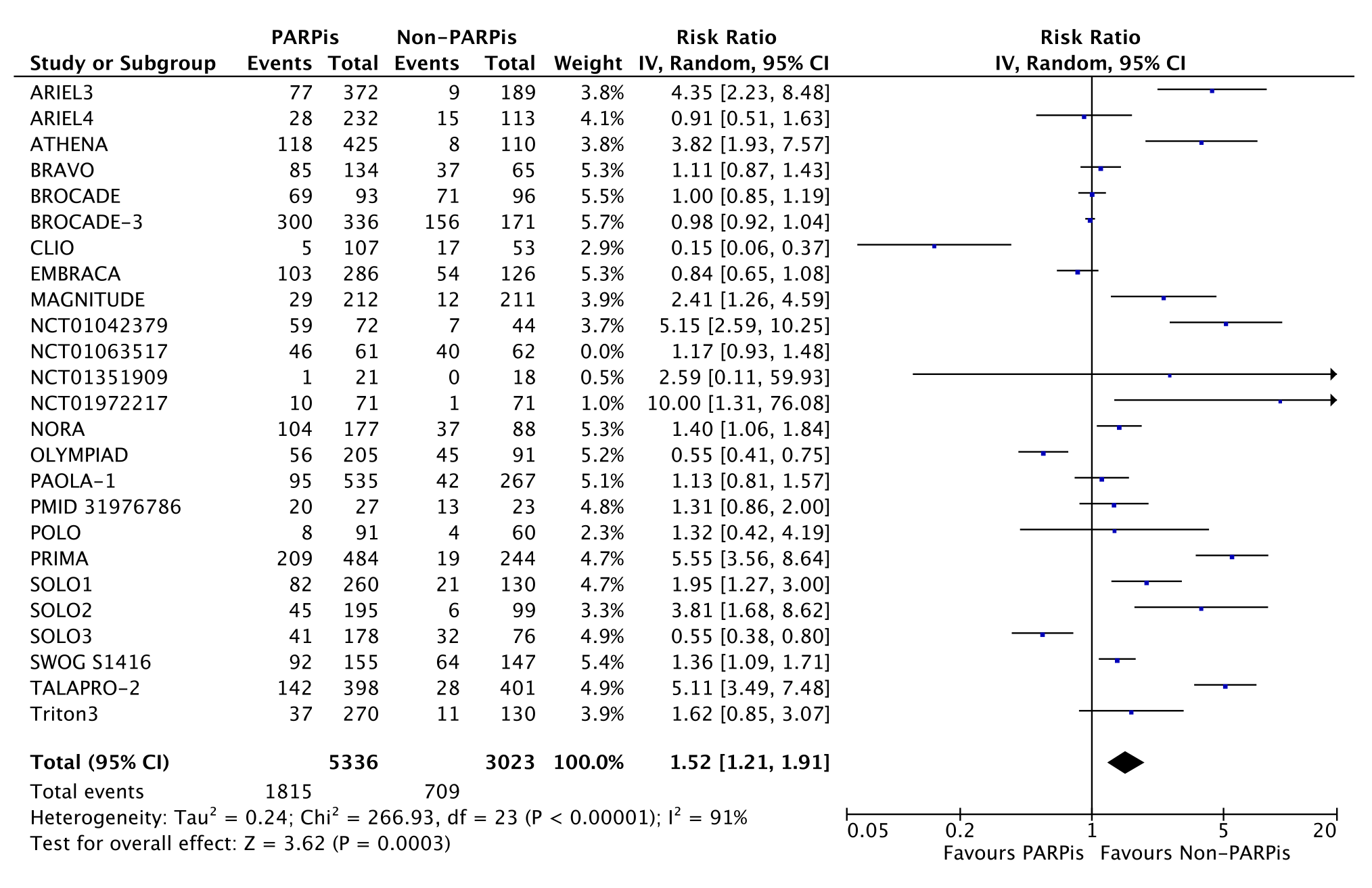 | 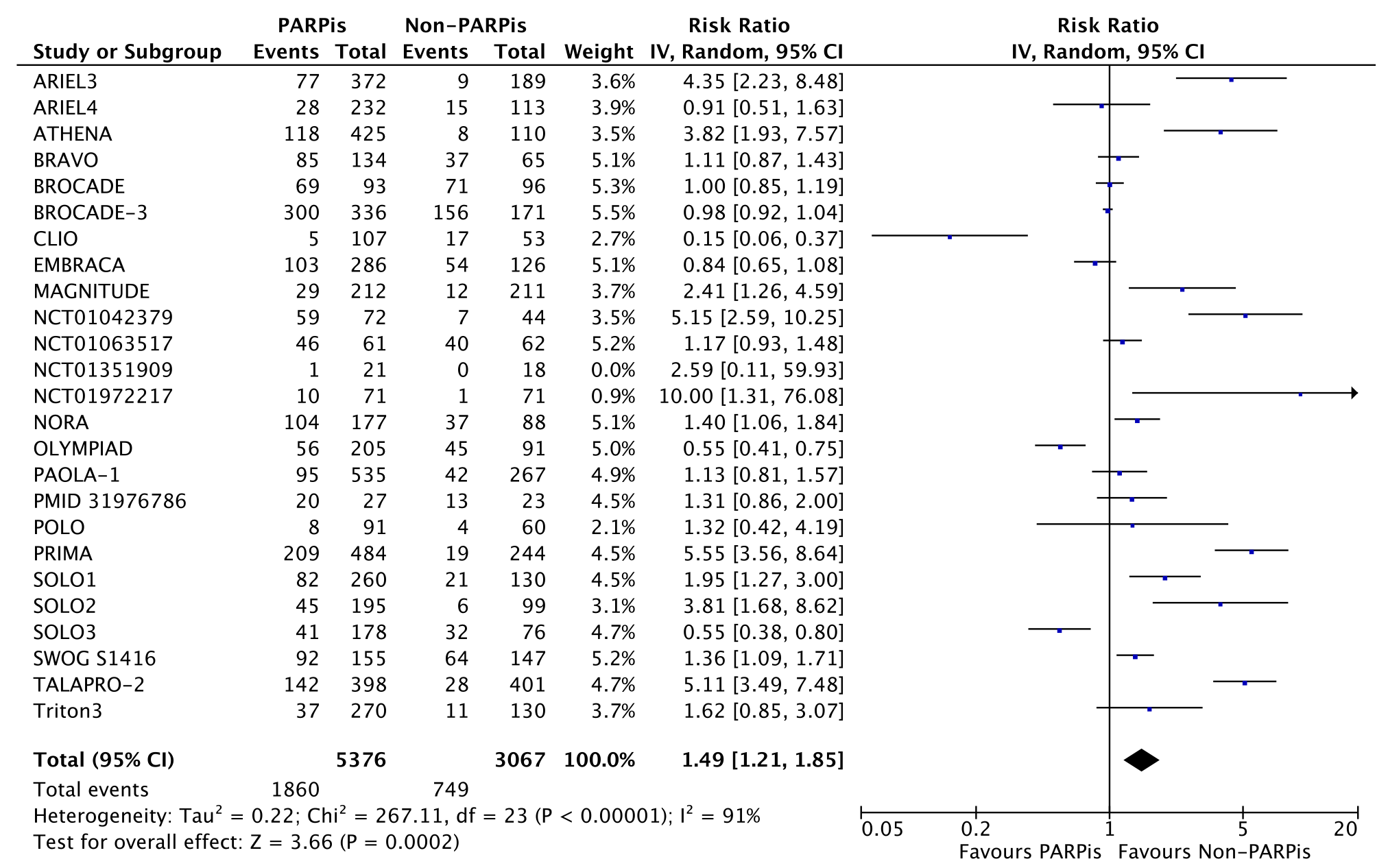 |
| 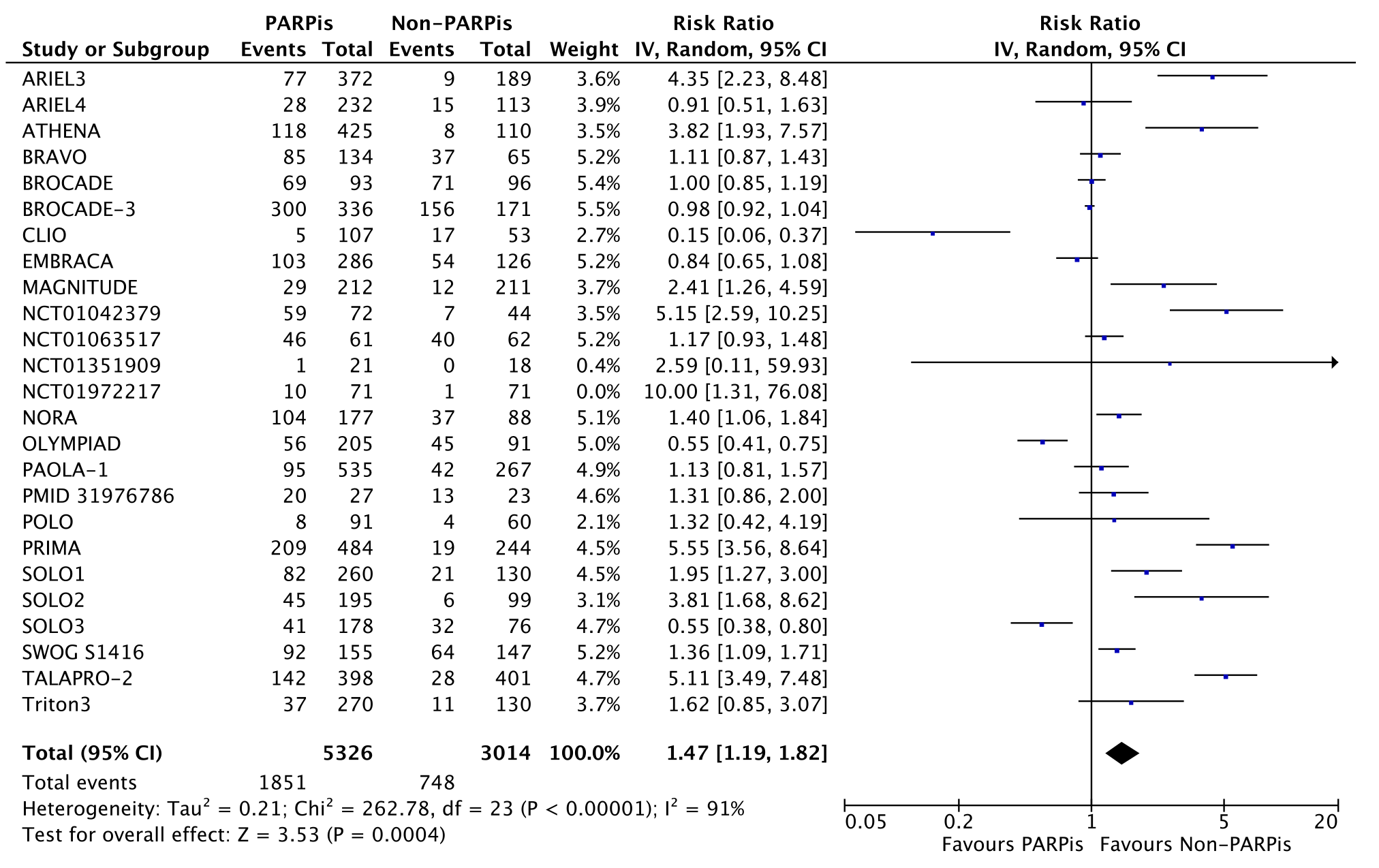 | 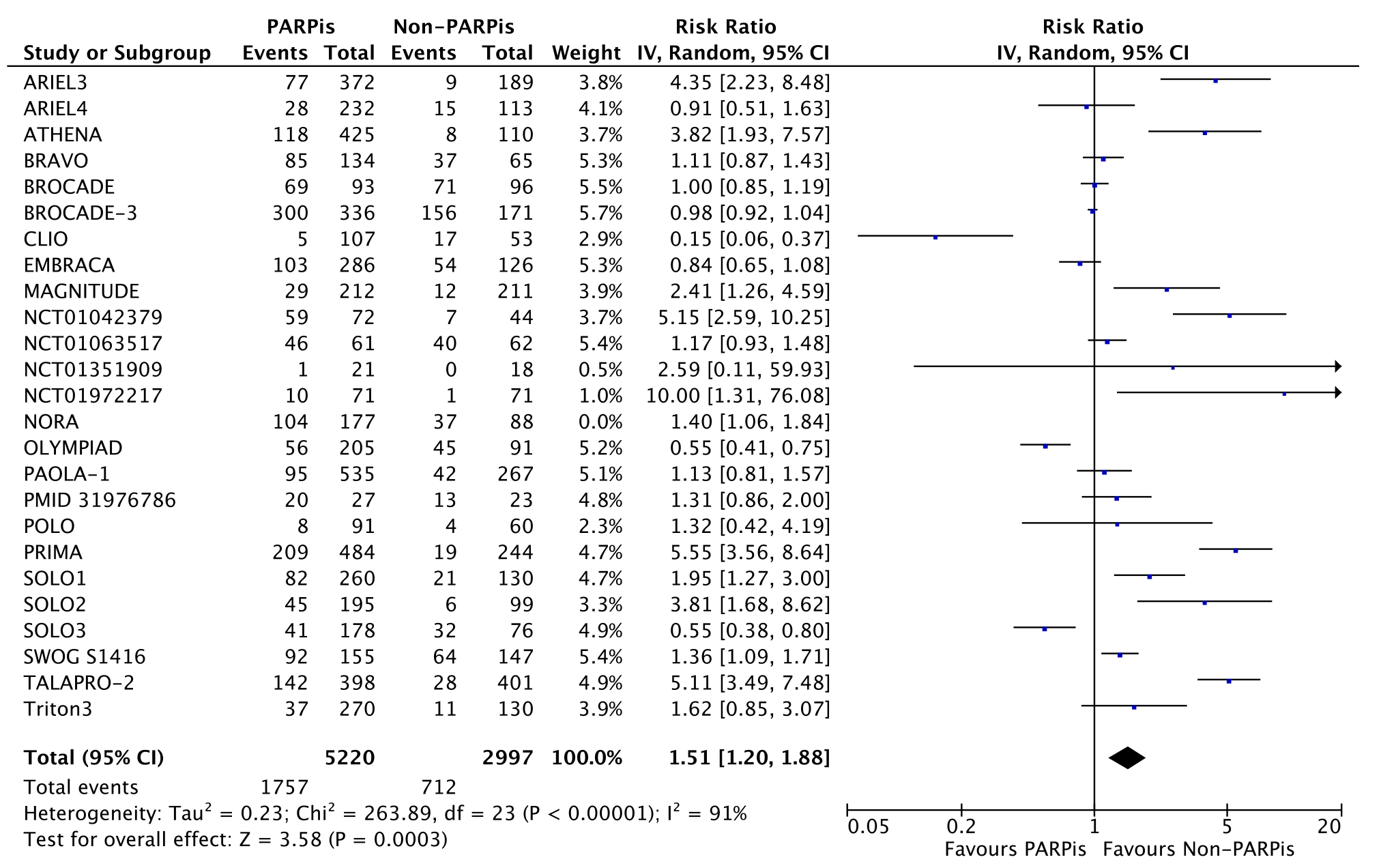 |
| 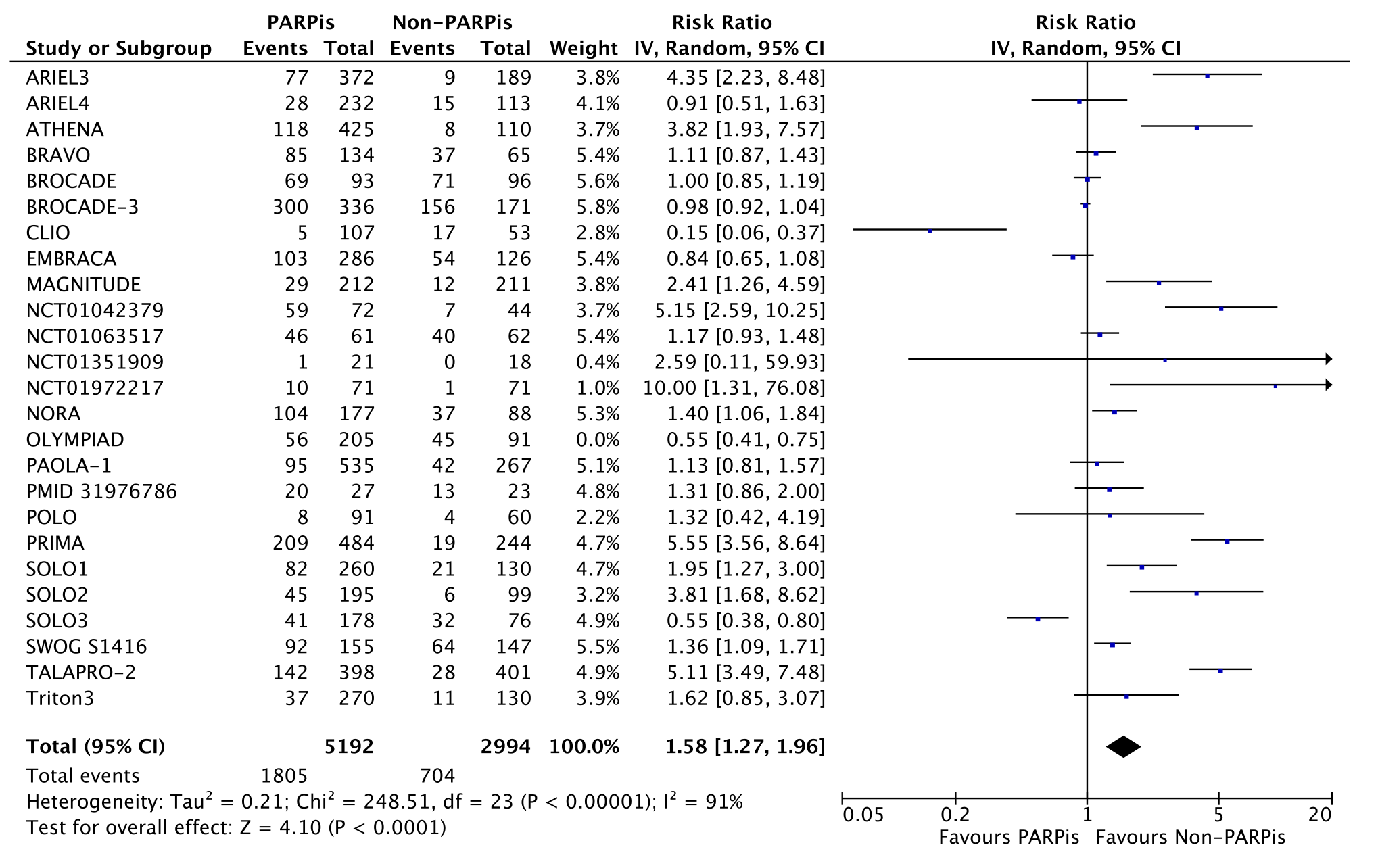 | 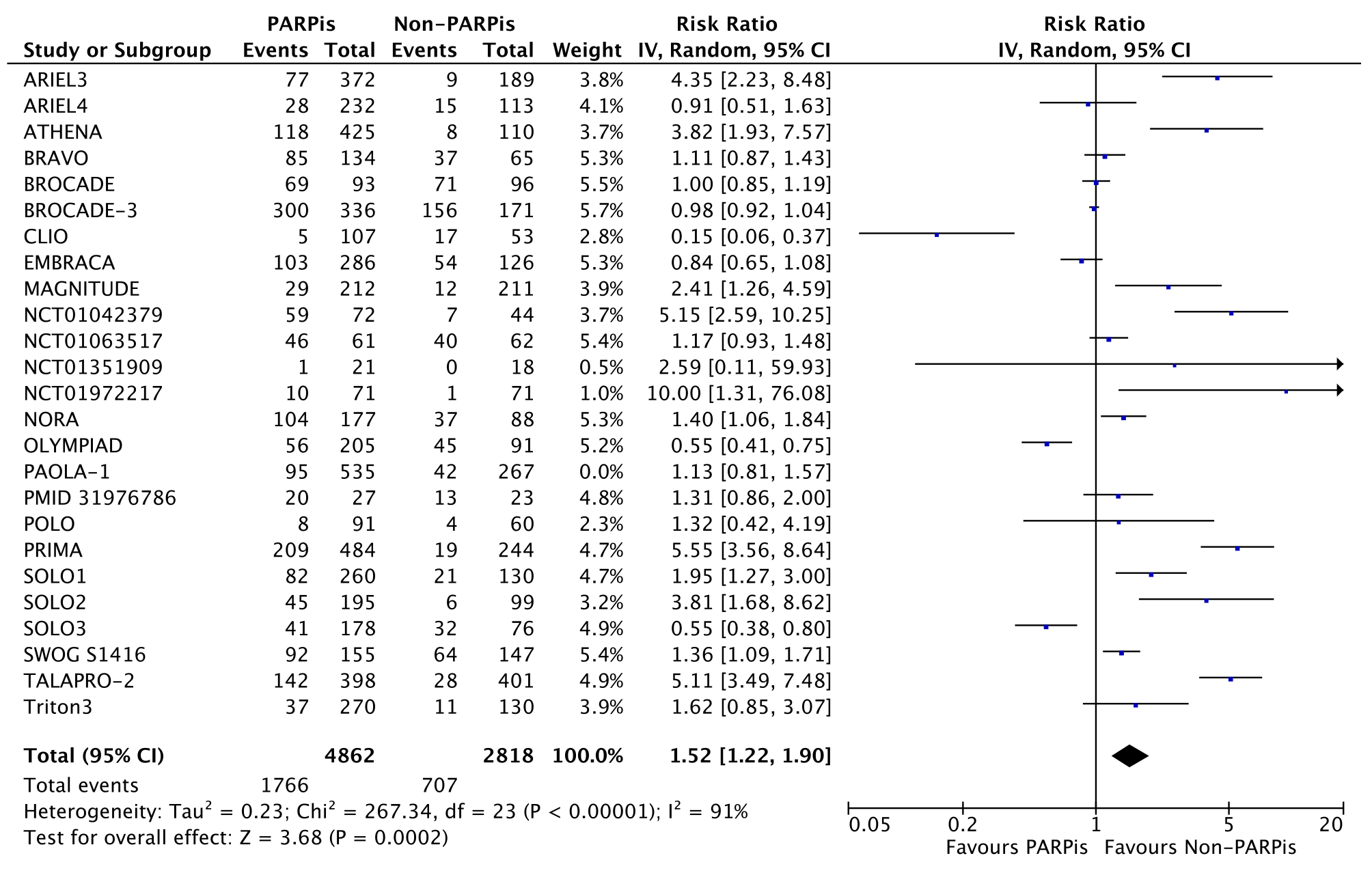 |
| 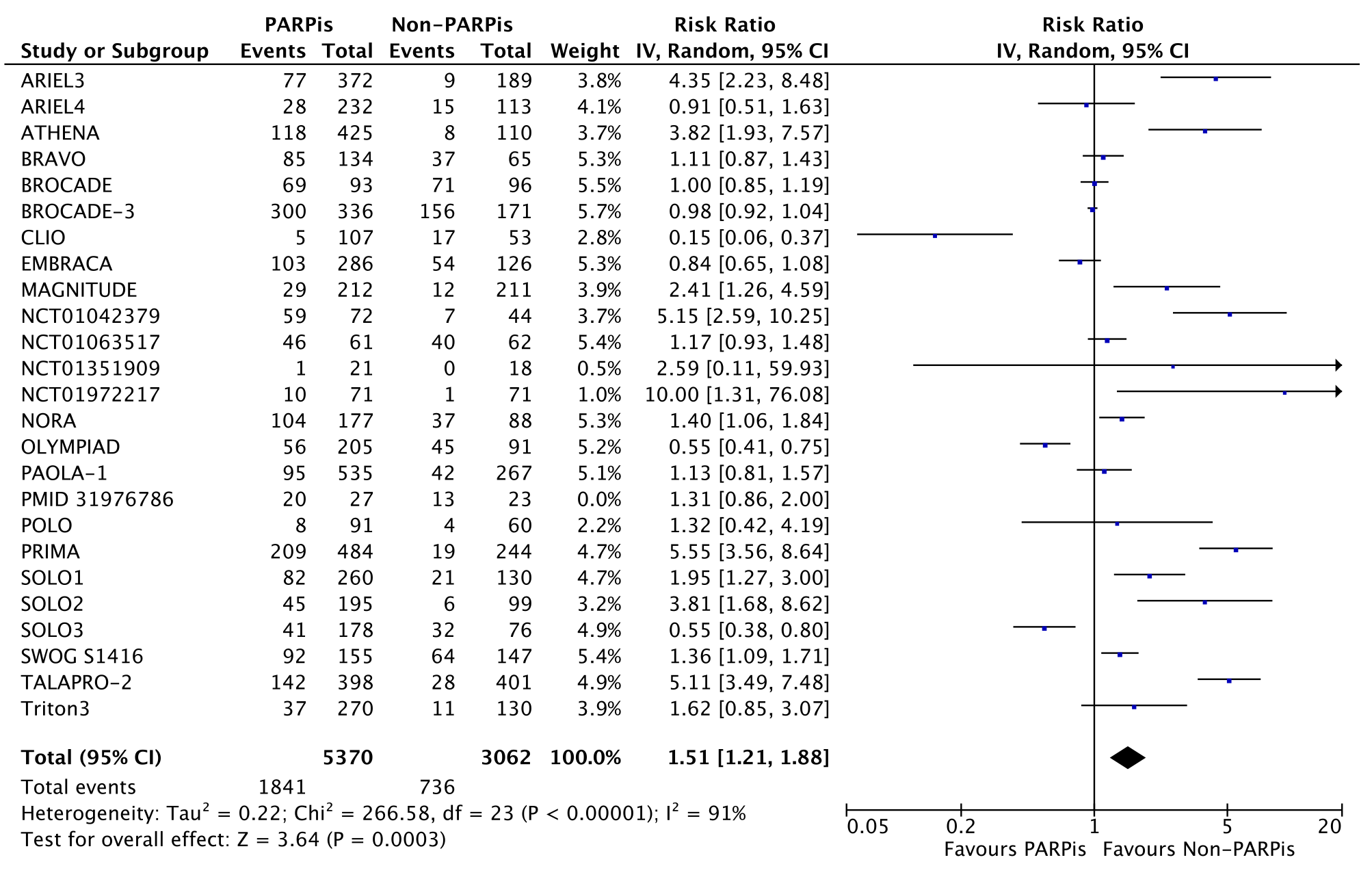 | 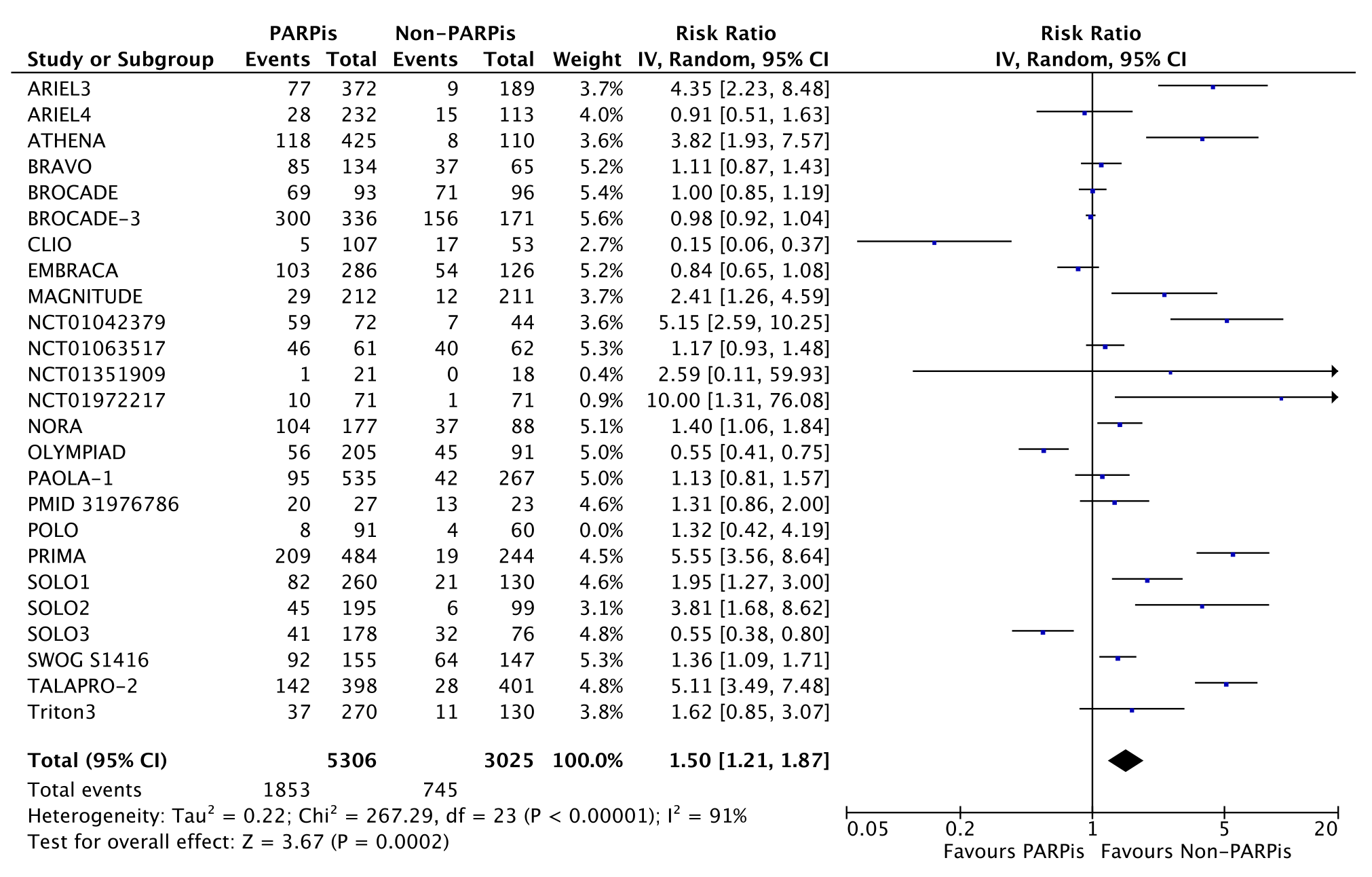 |
| 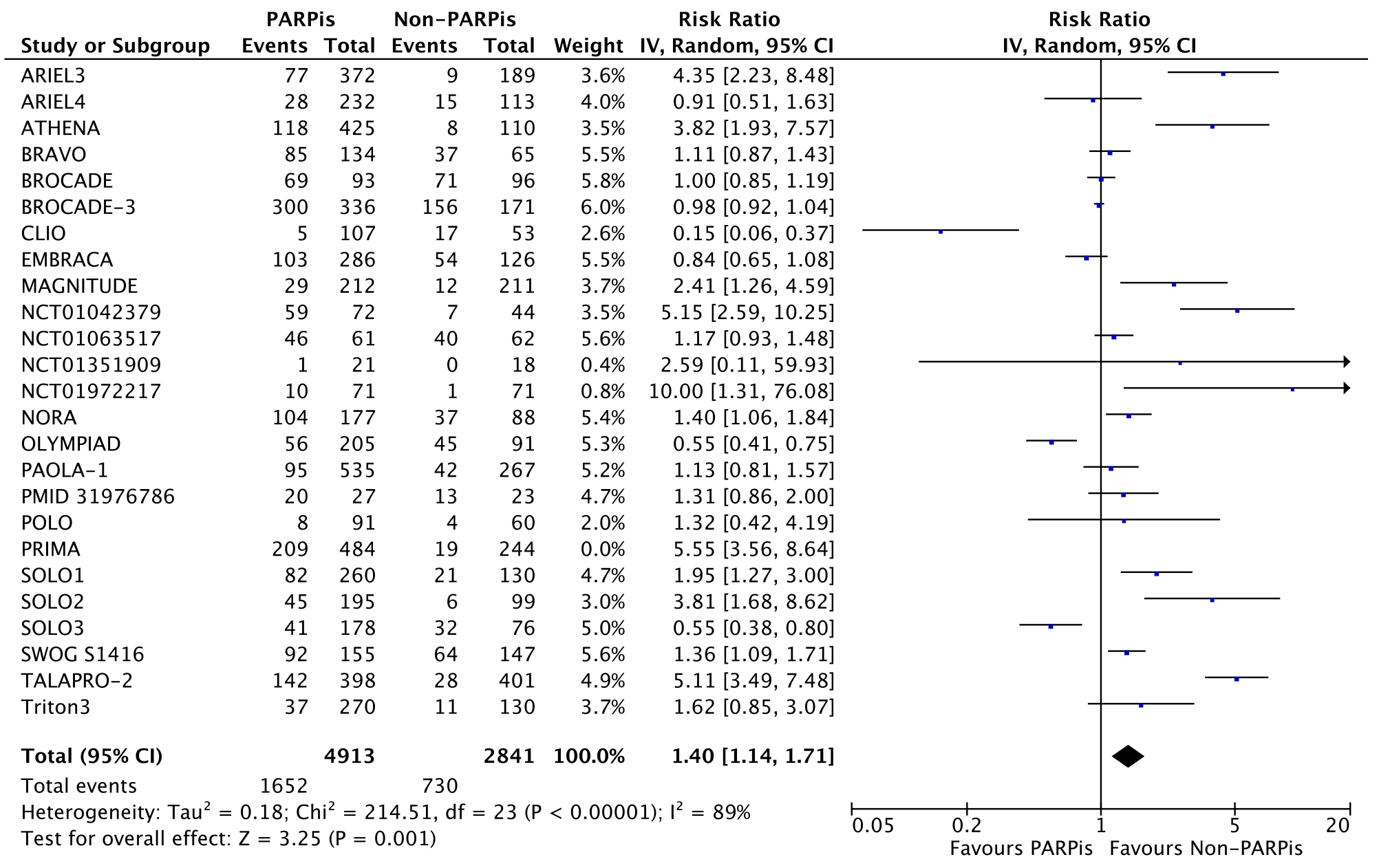 | 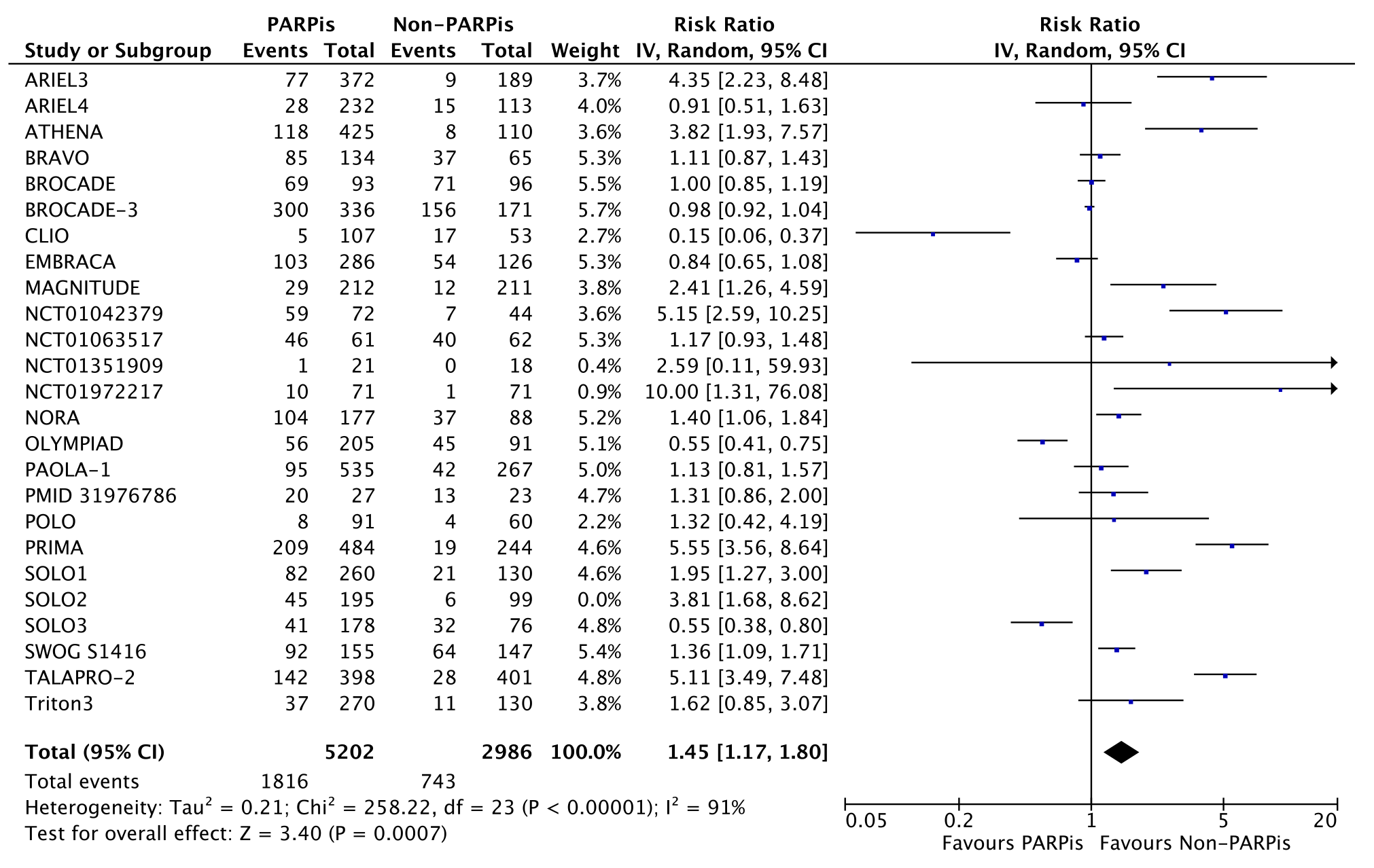 |
| 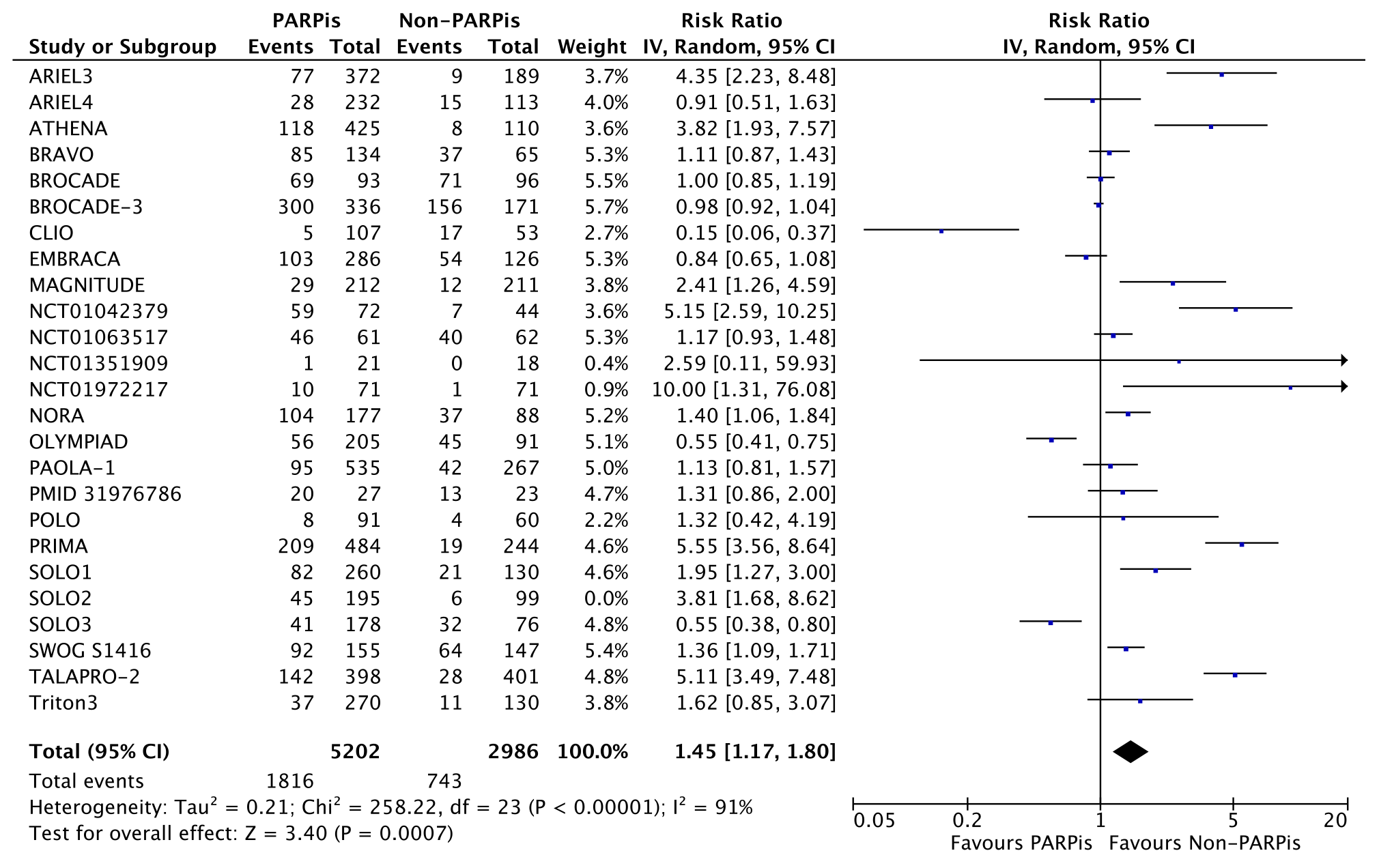 | 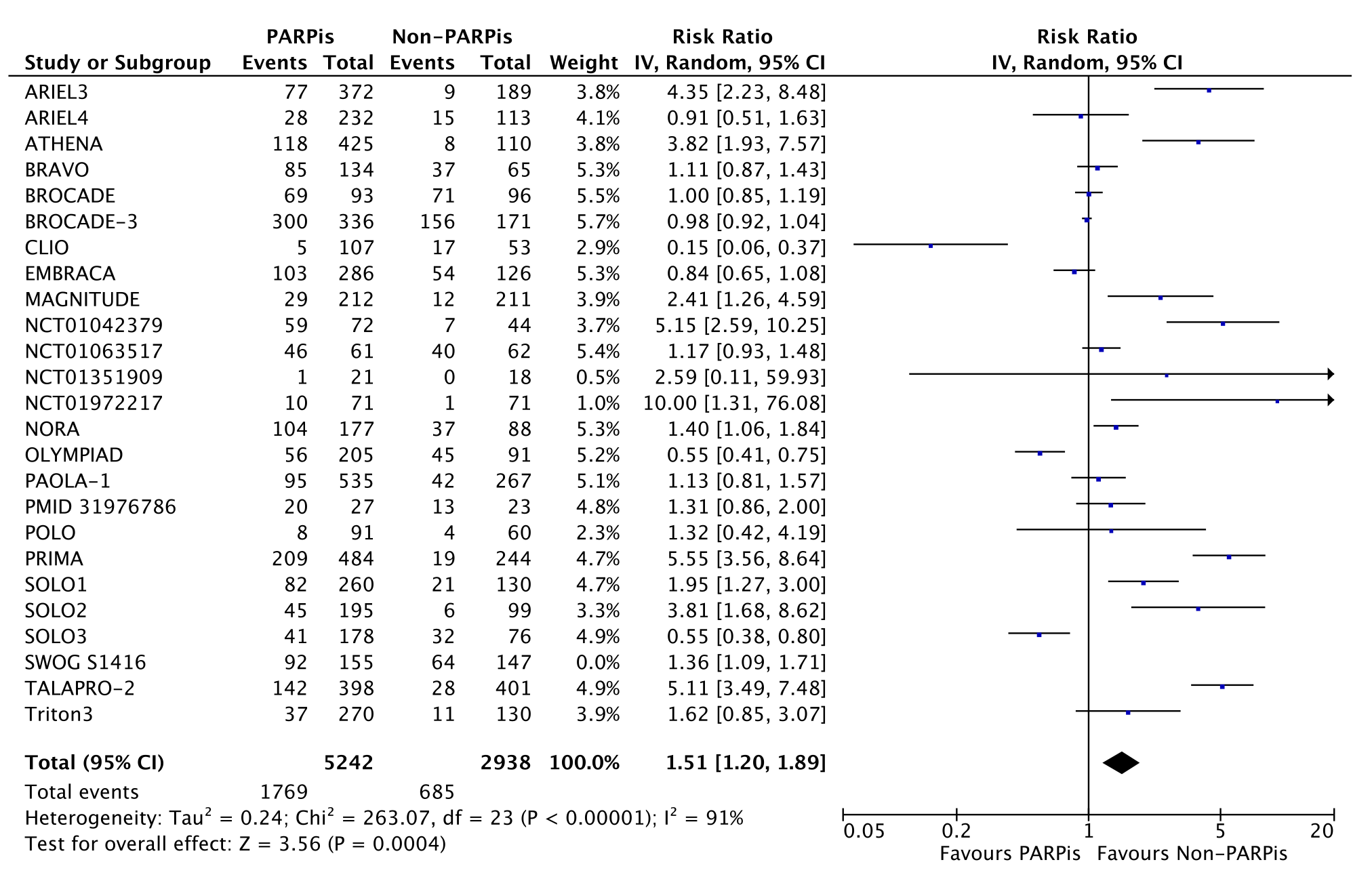 |
| 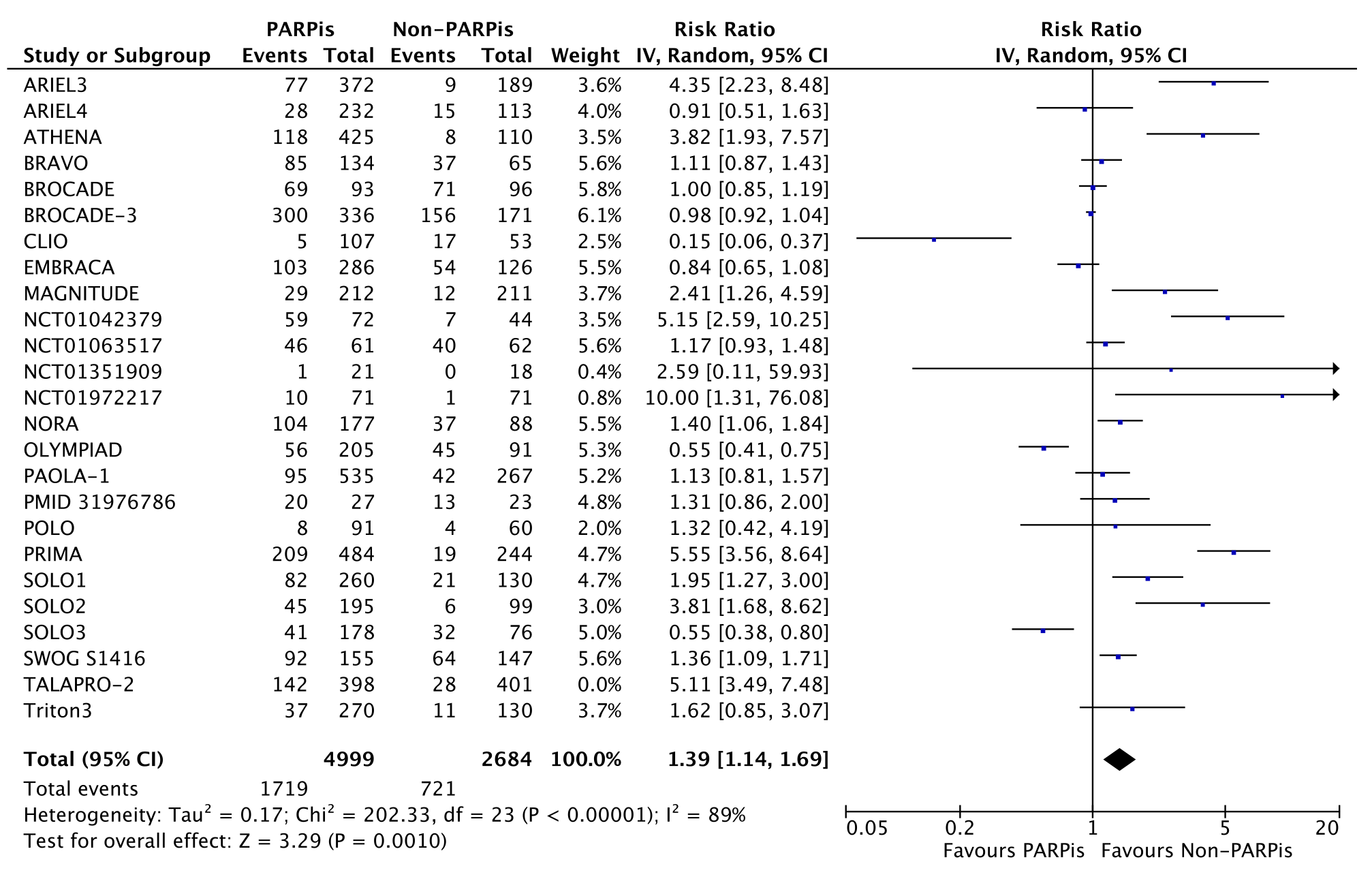 | 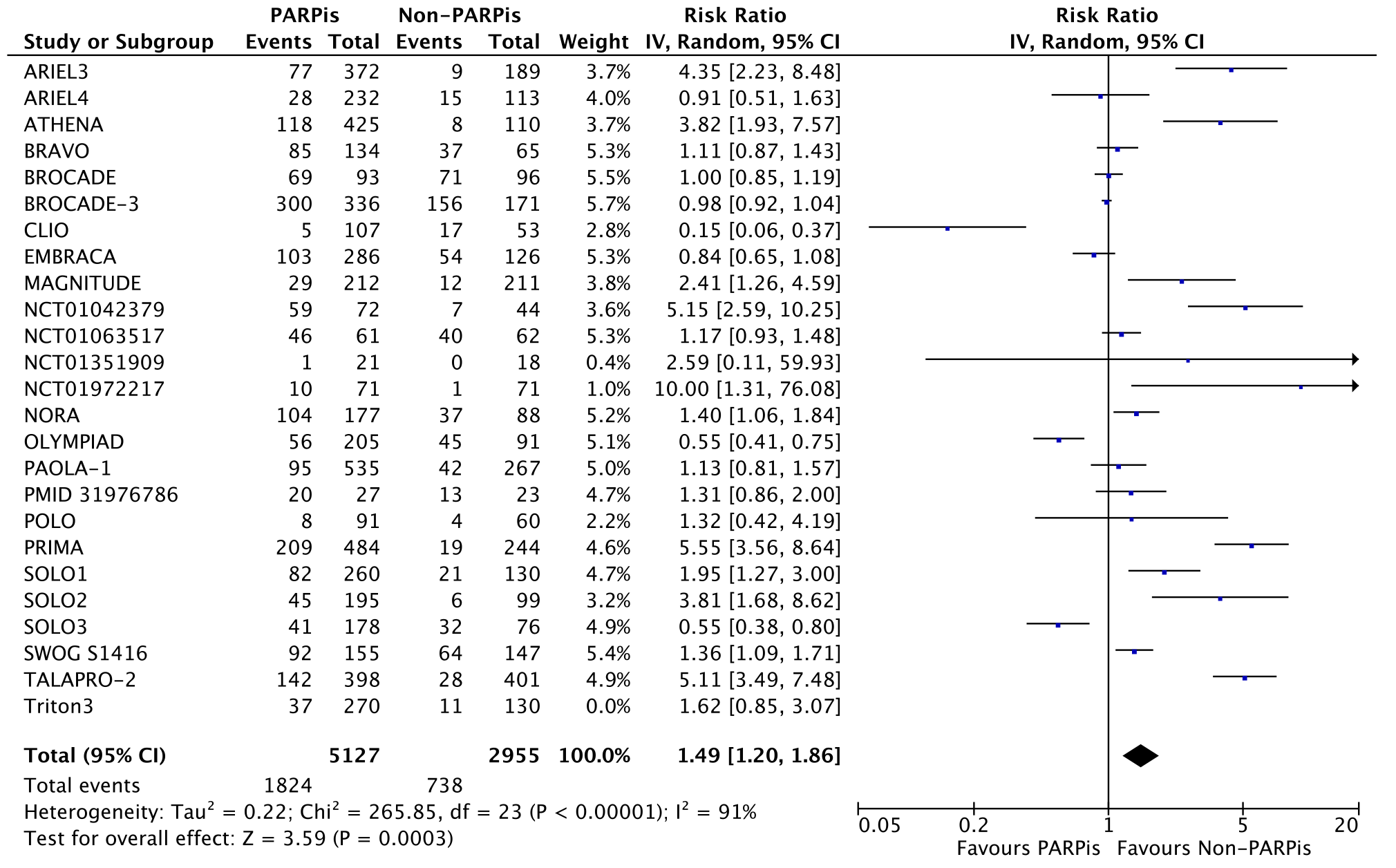 |

C

| 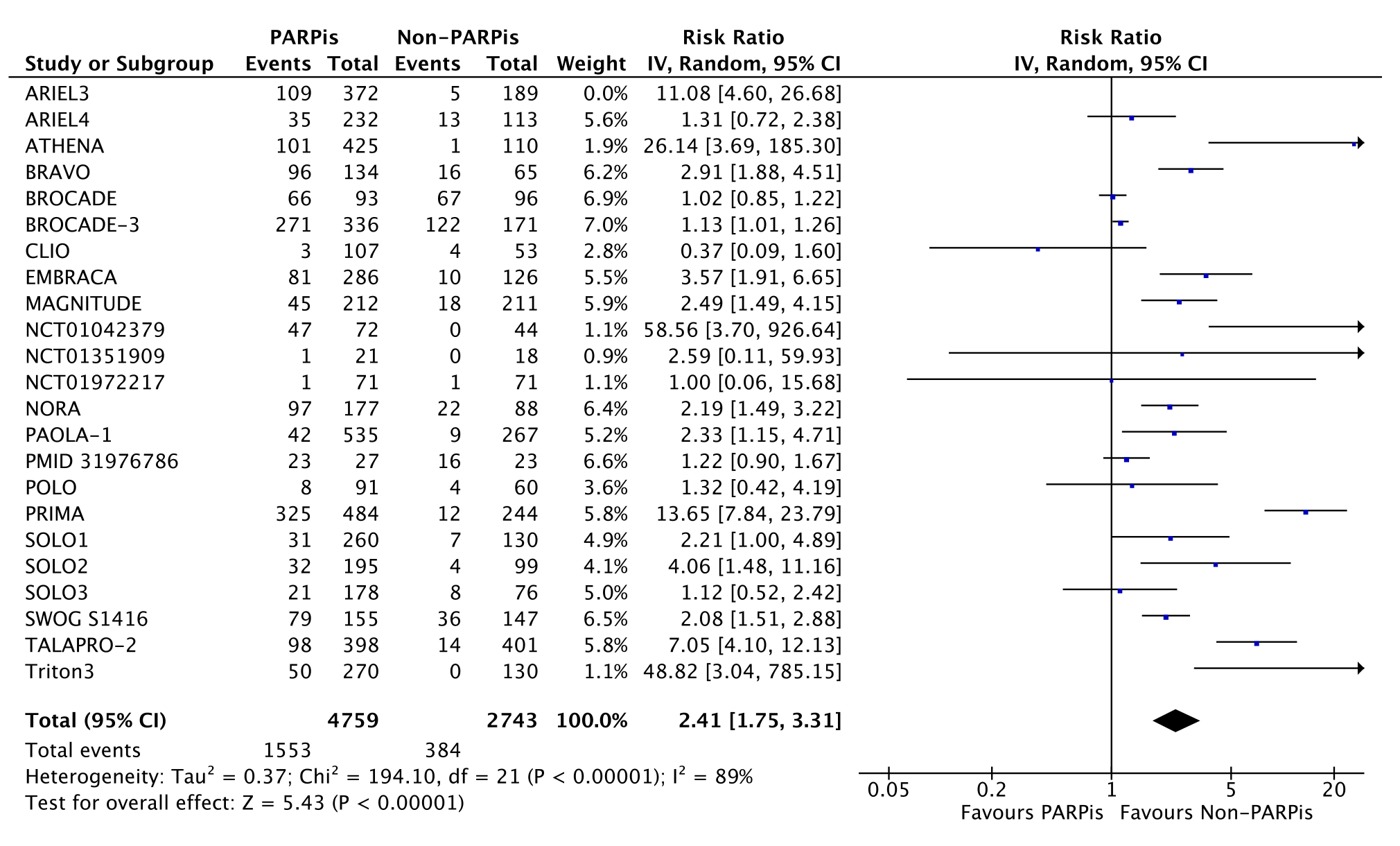 | 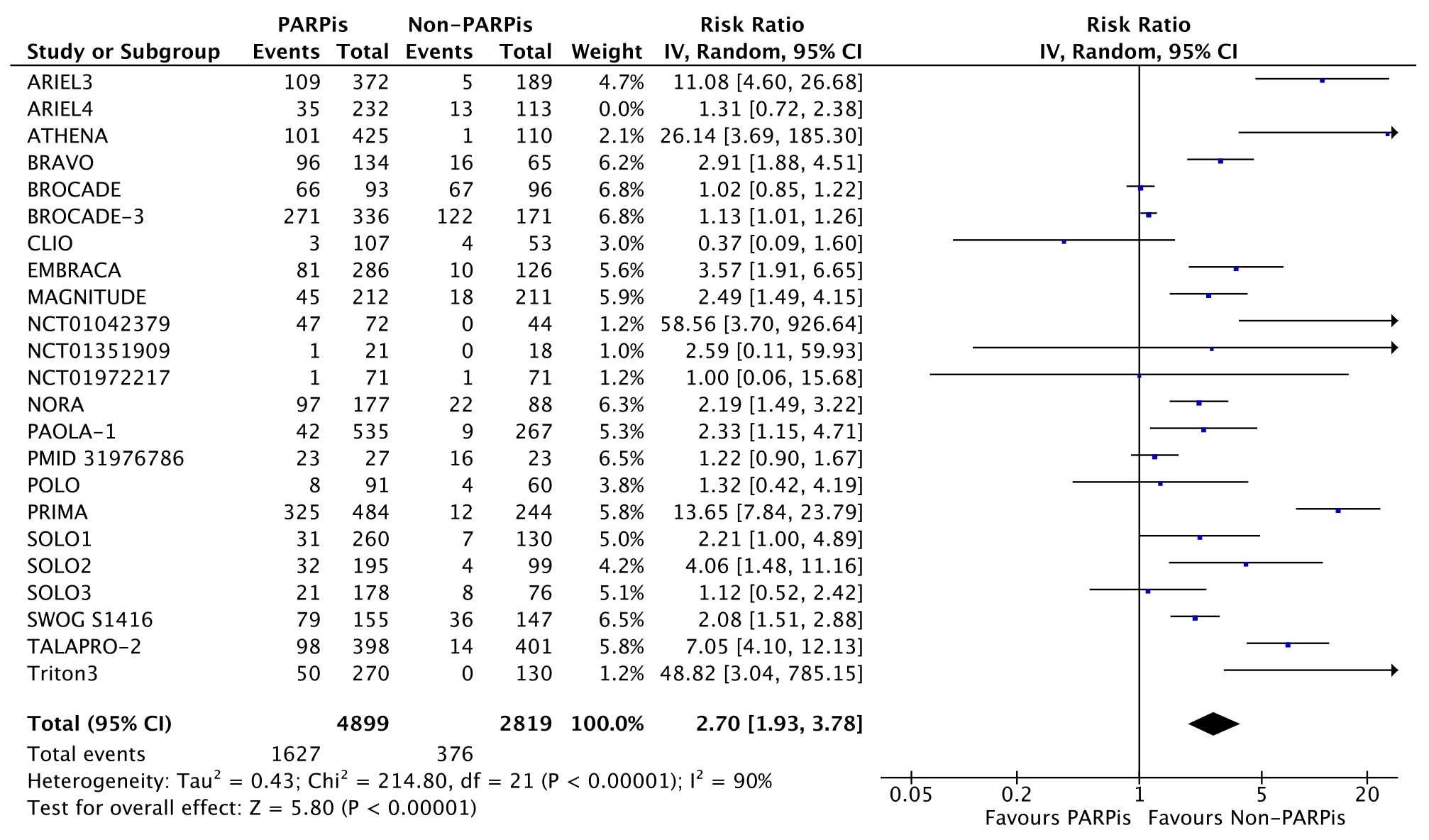 |
| --- | --- |
| 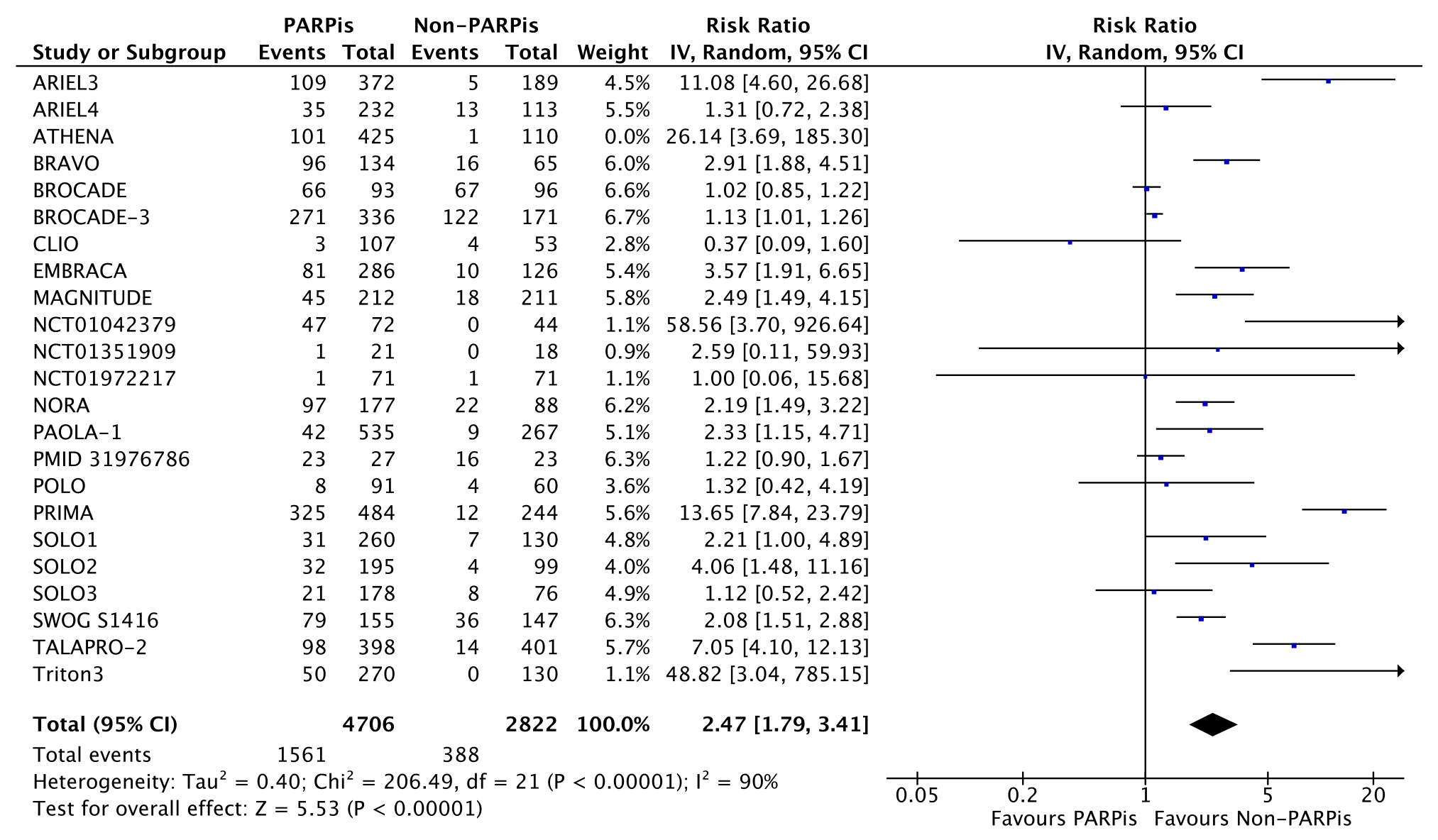 | 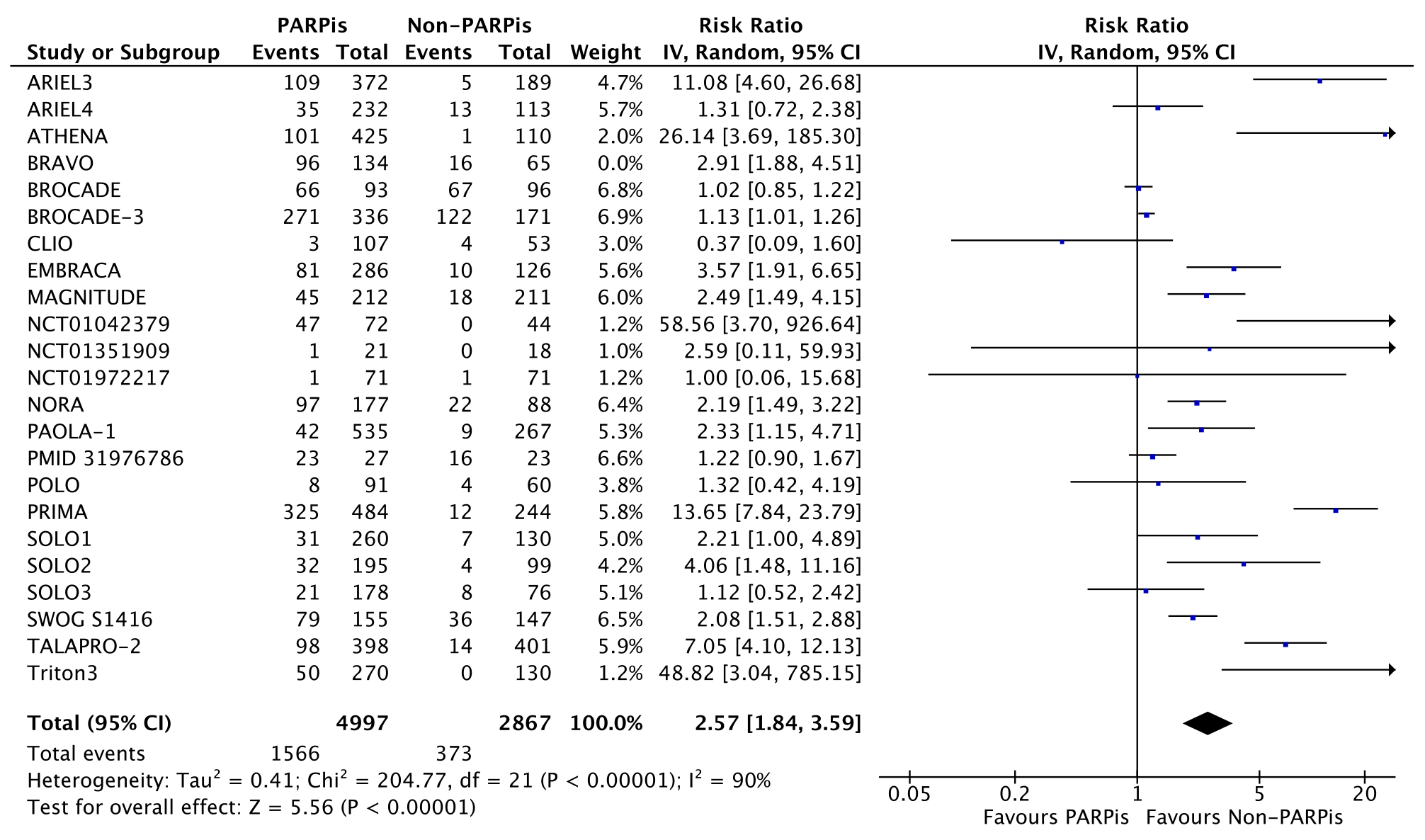 |
| 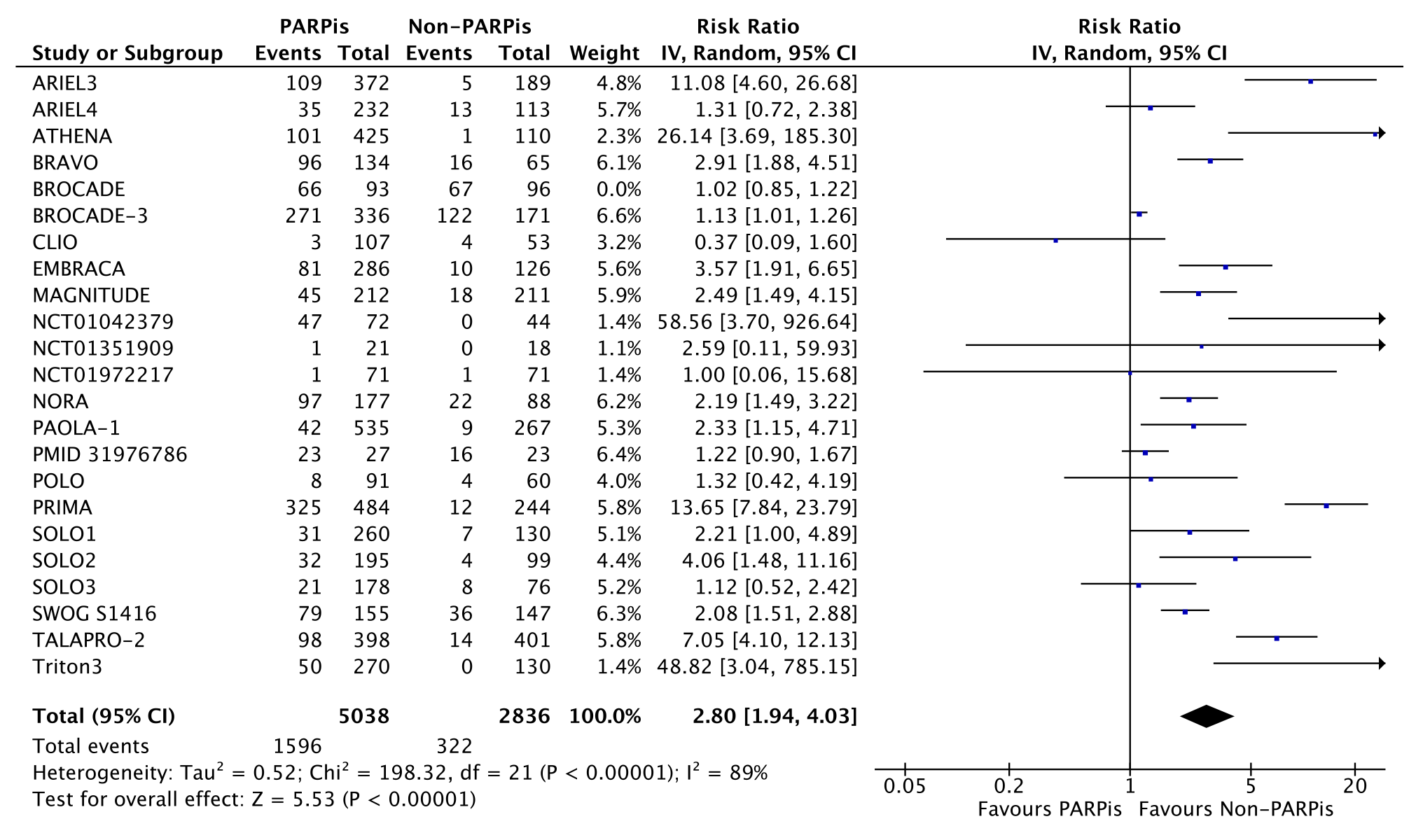 | 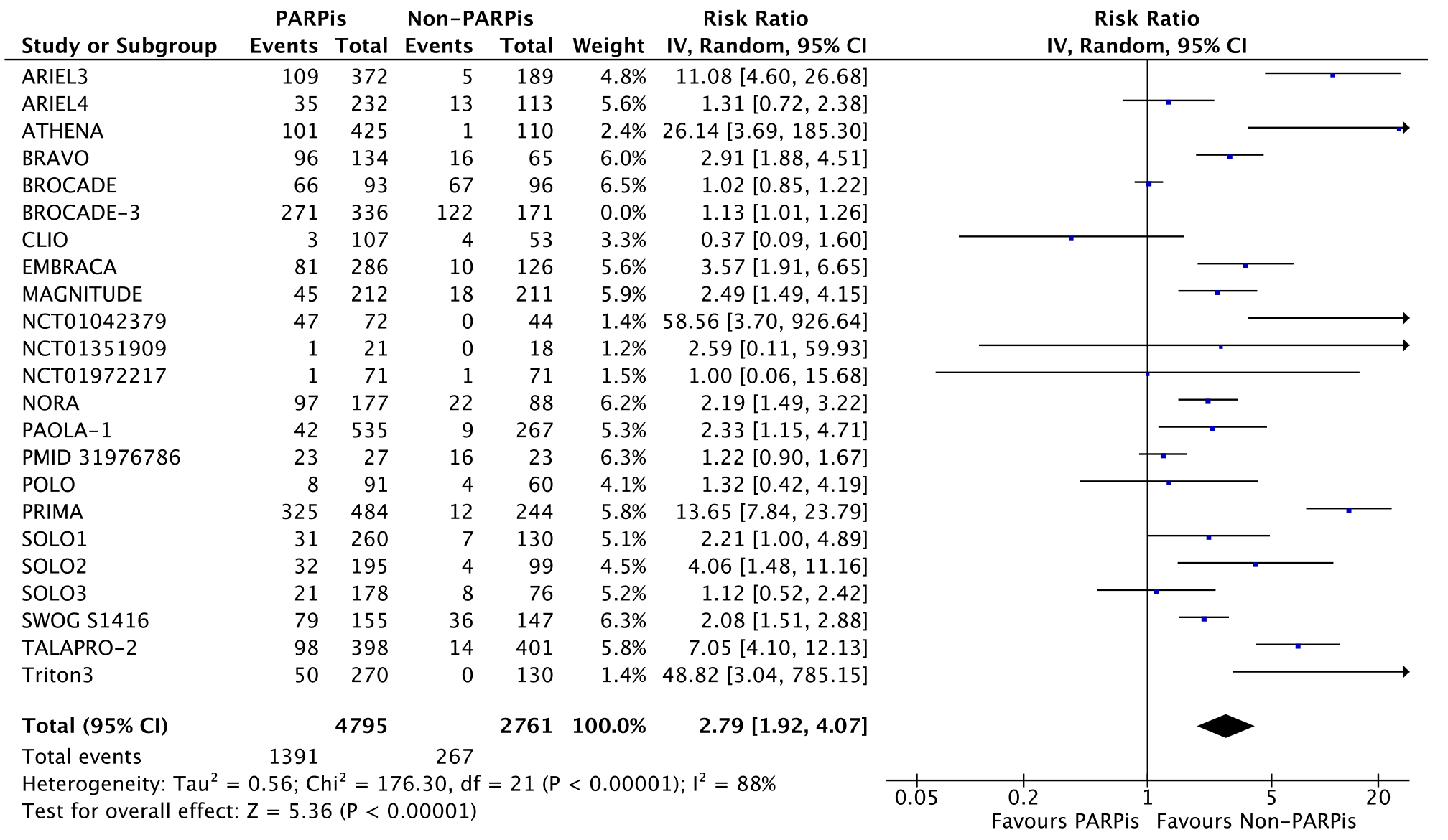 |
| 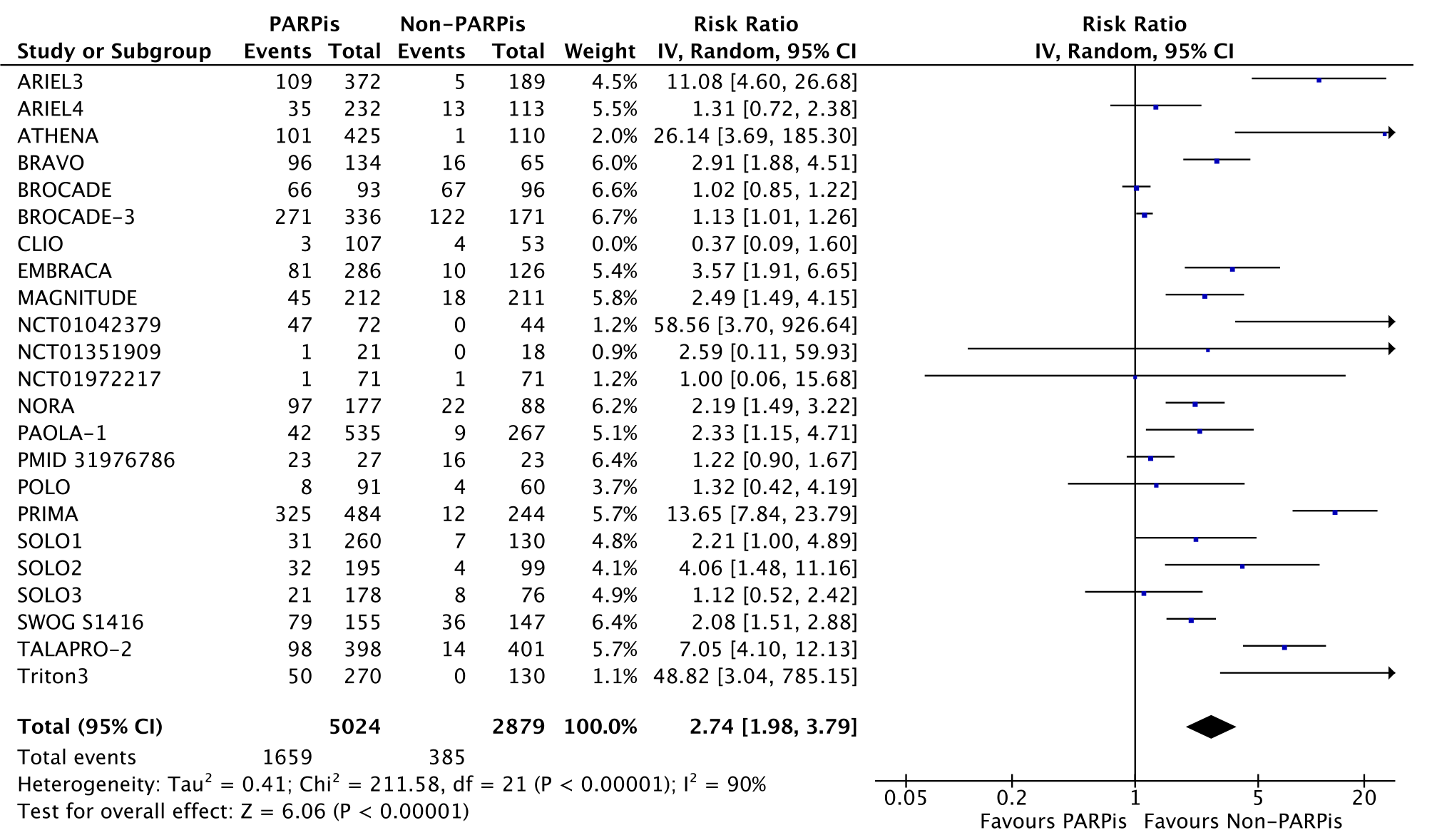 | 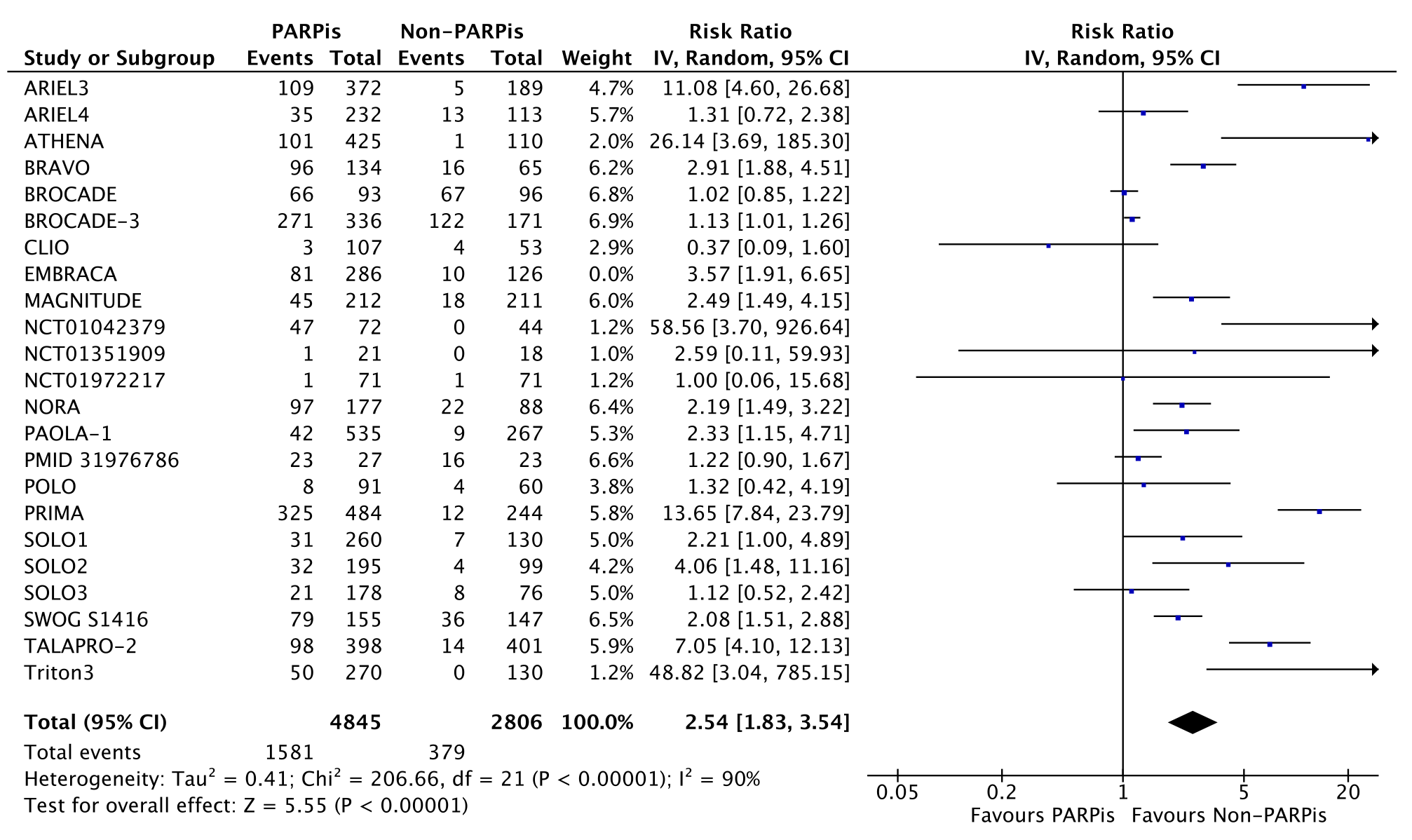 |
| 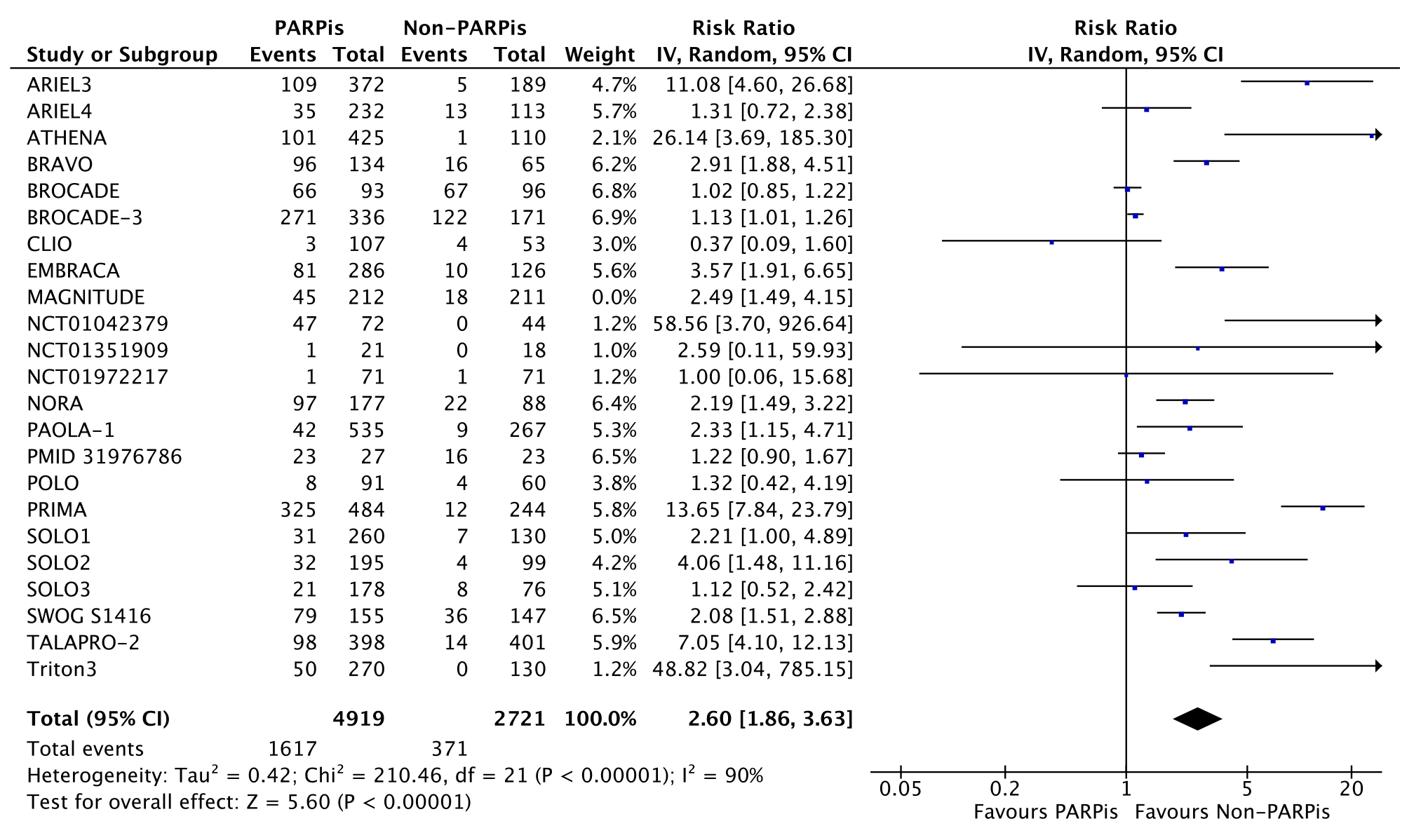 | 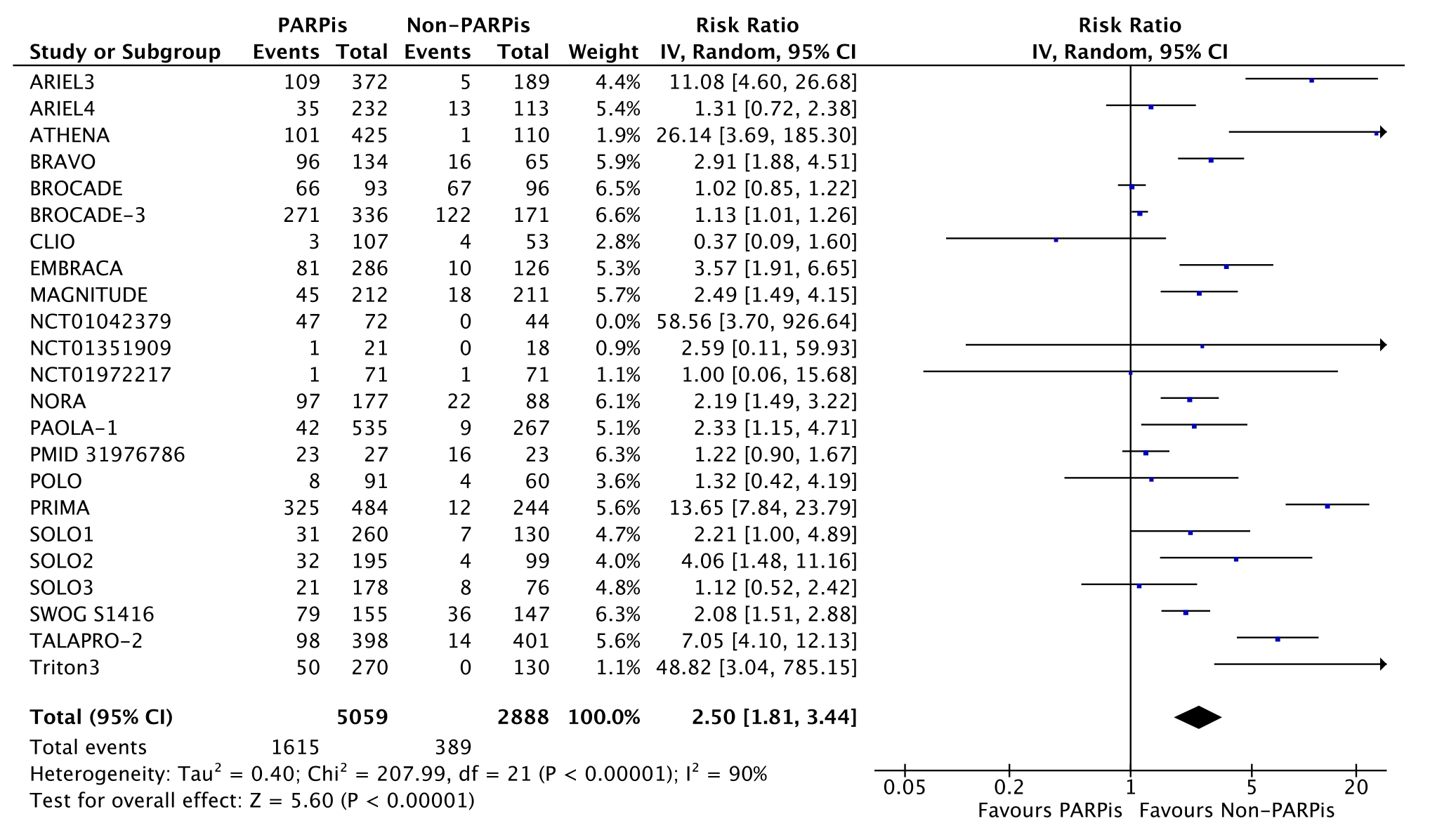 |
| 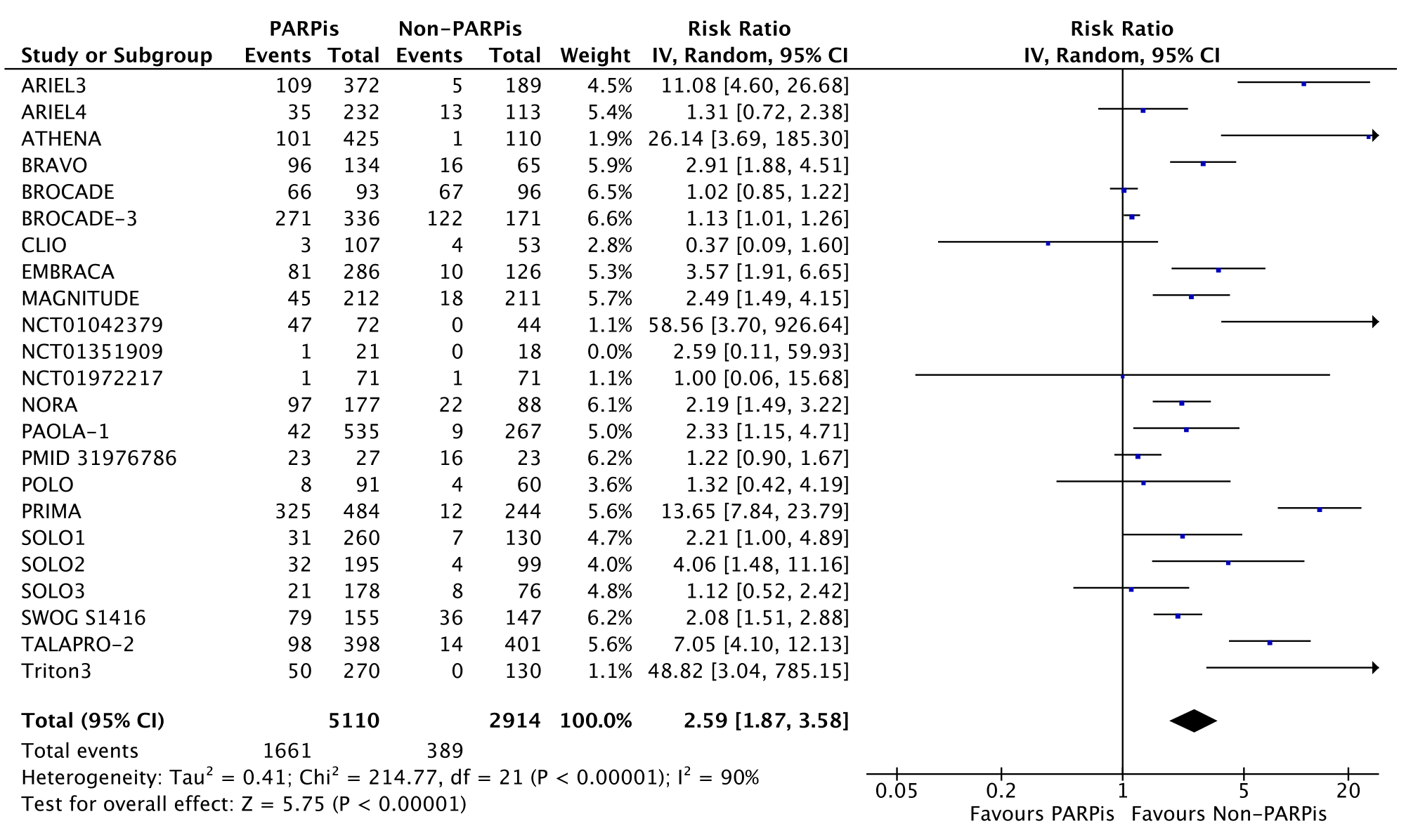 | 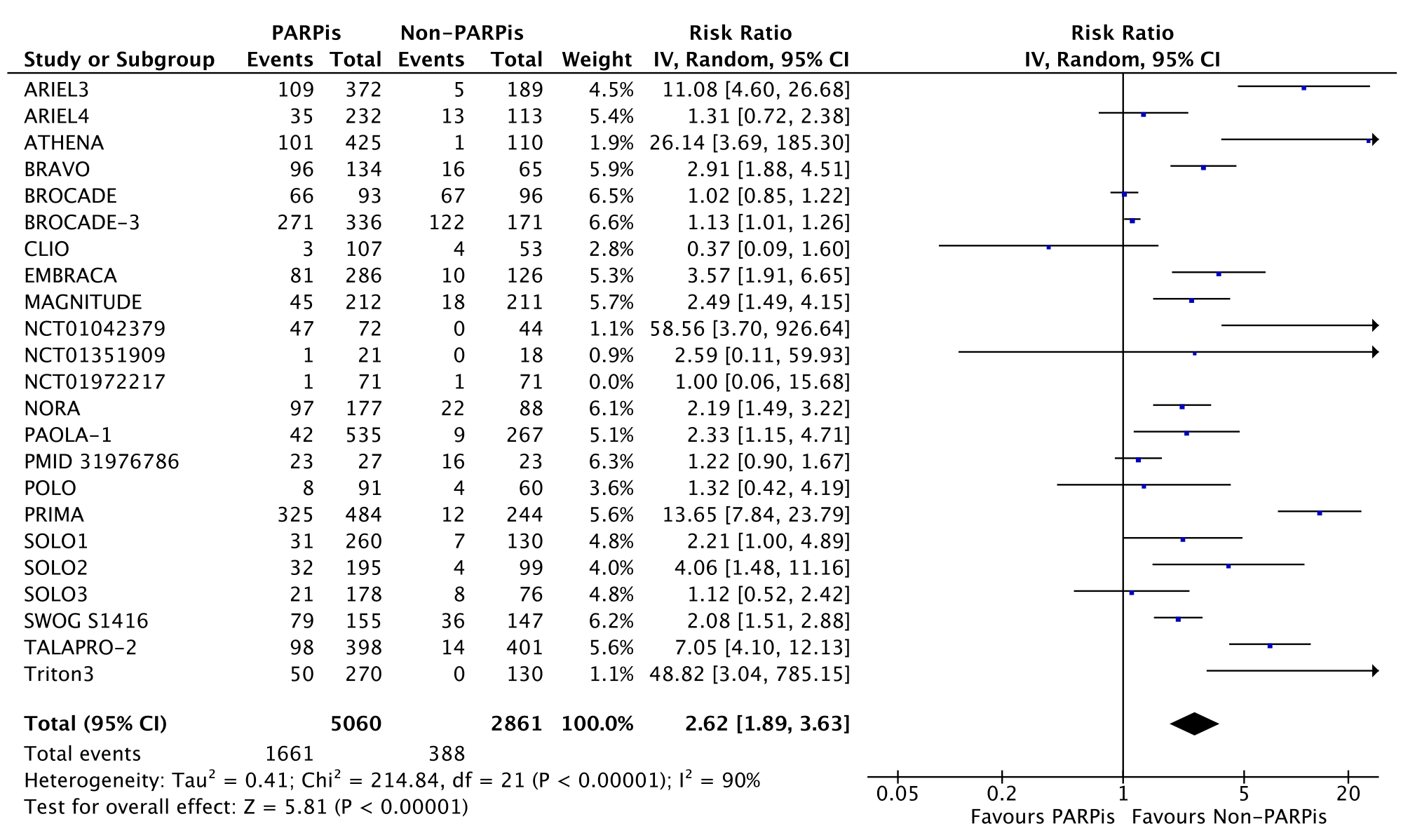 |
| 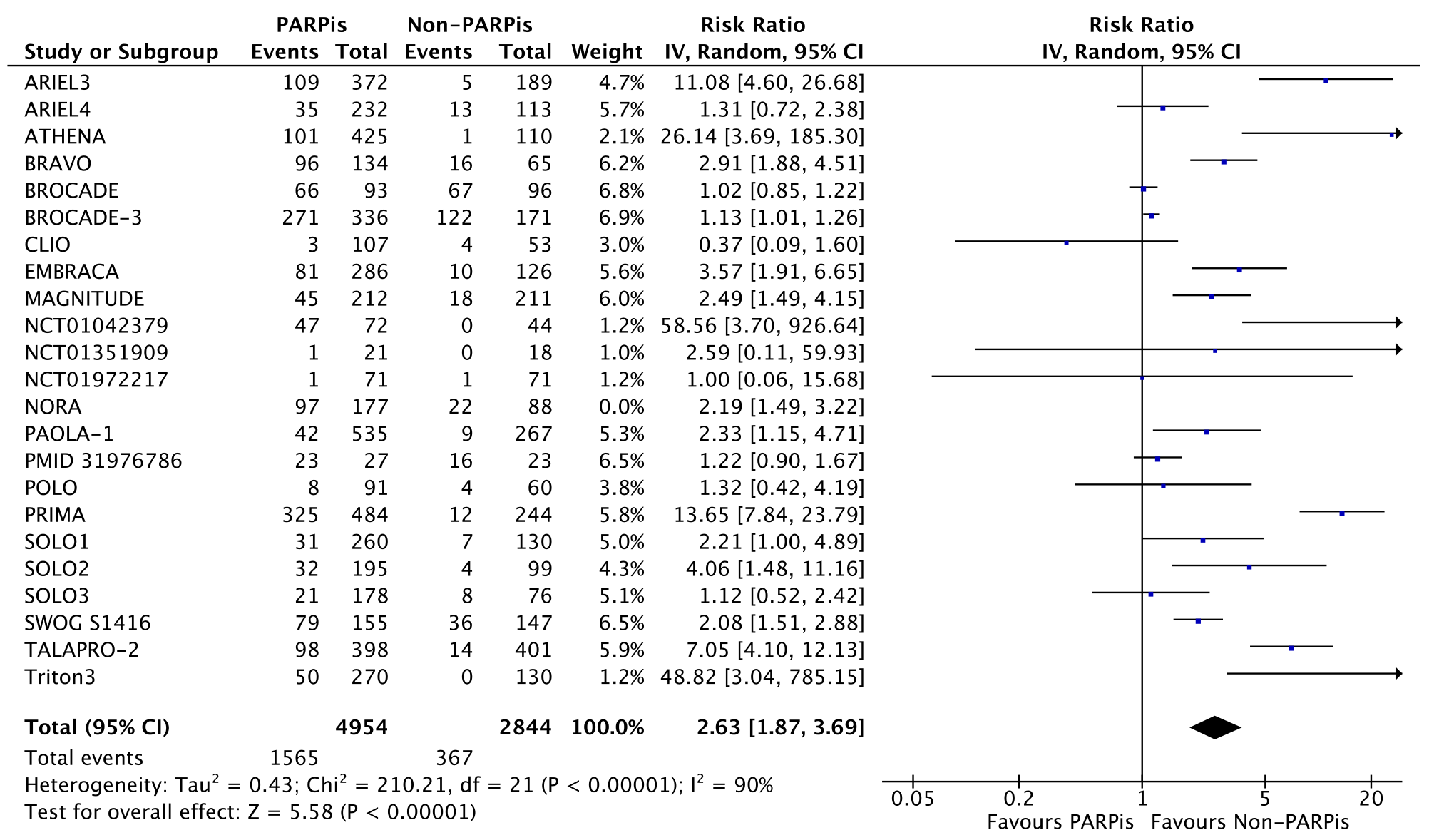 | 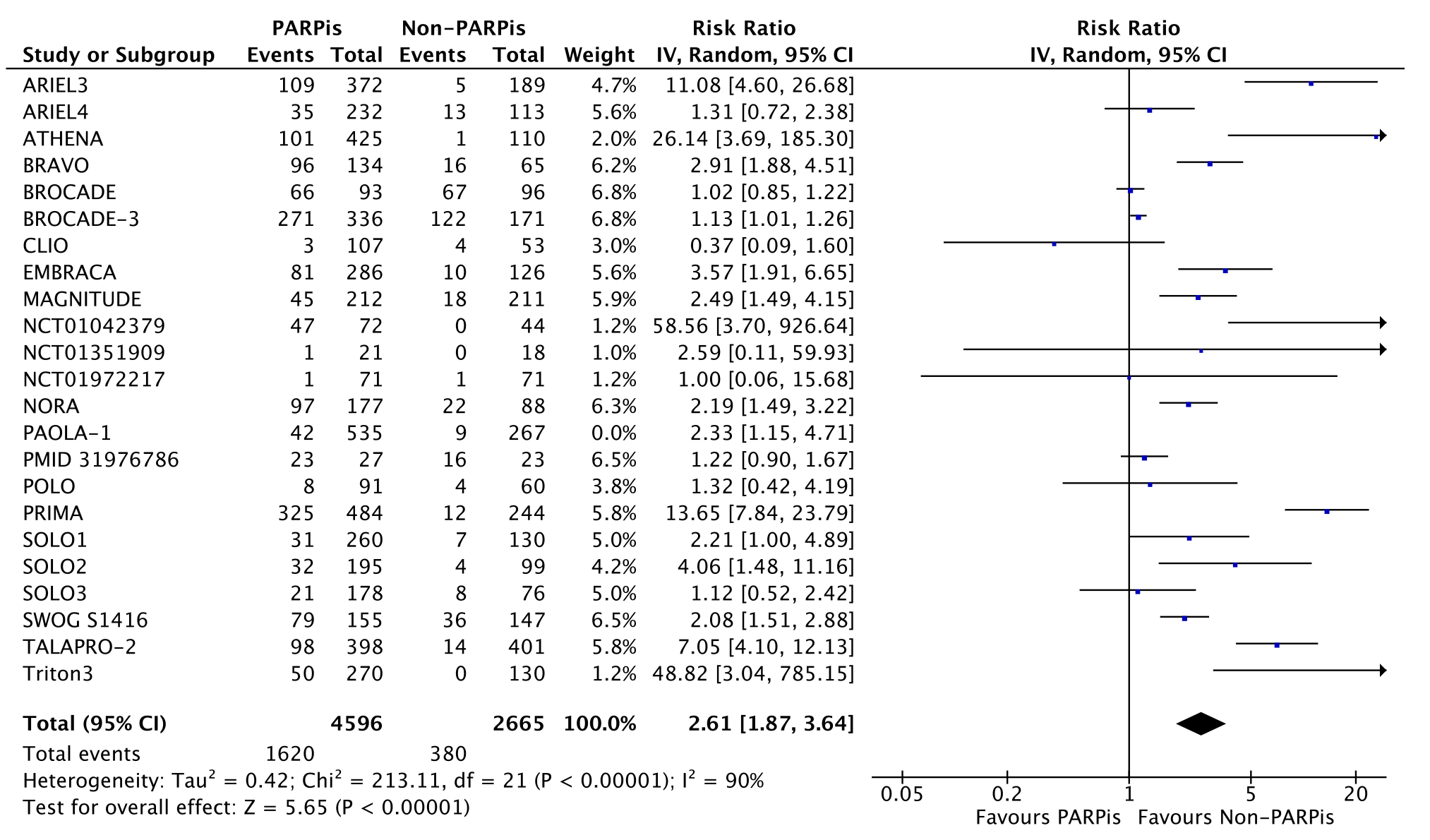 |
| 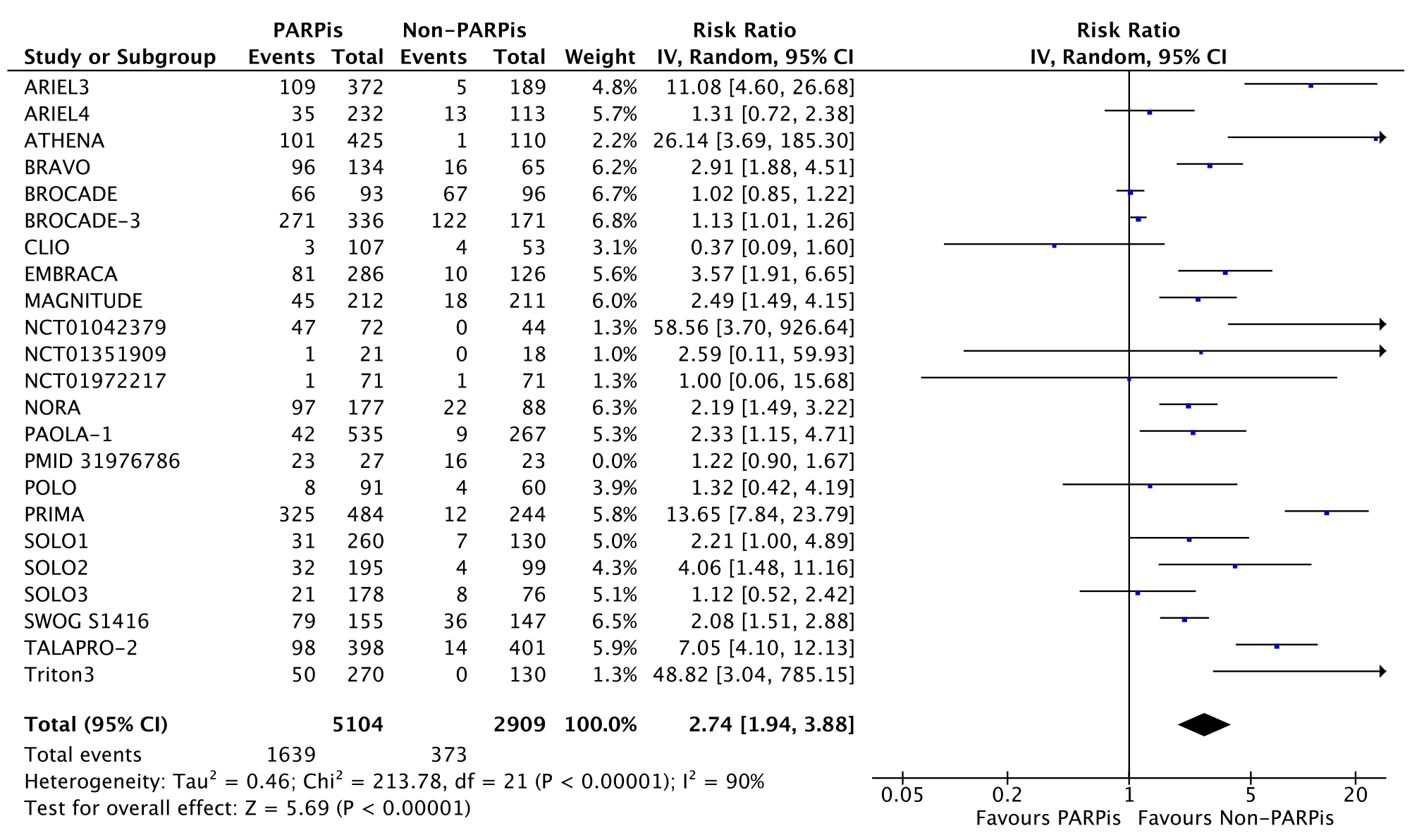 | 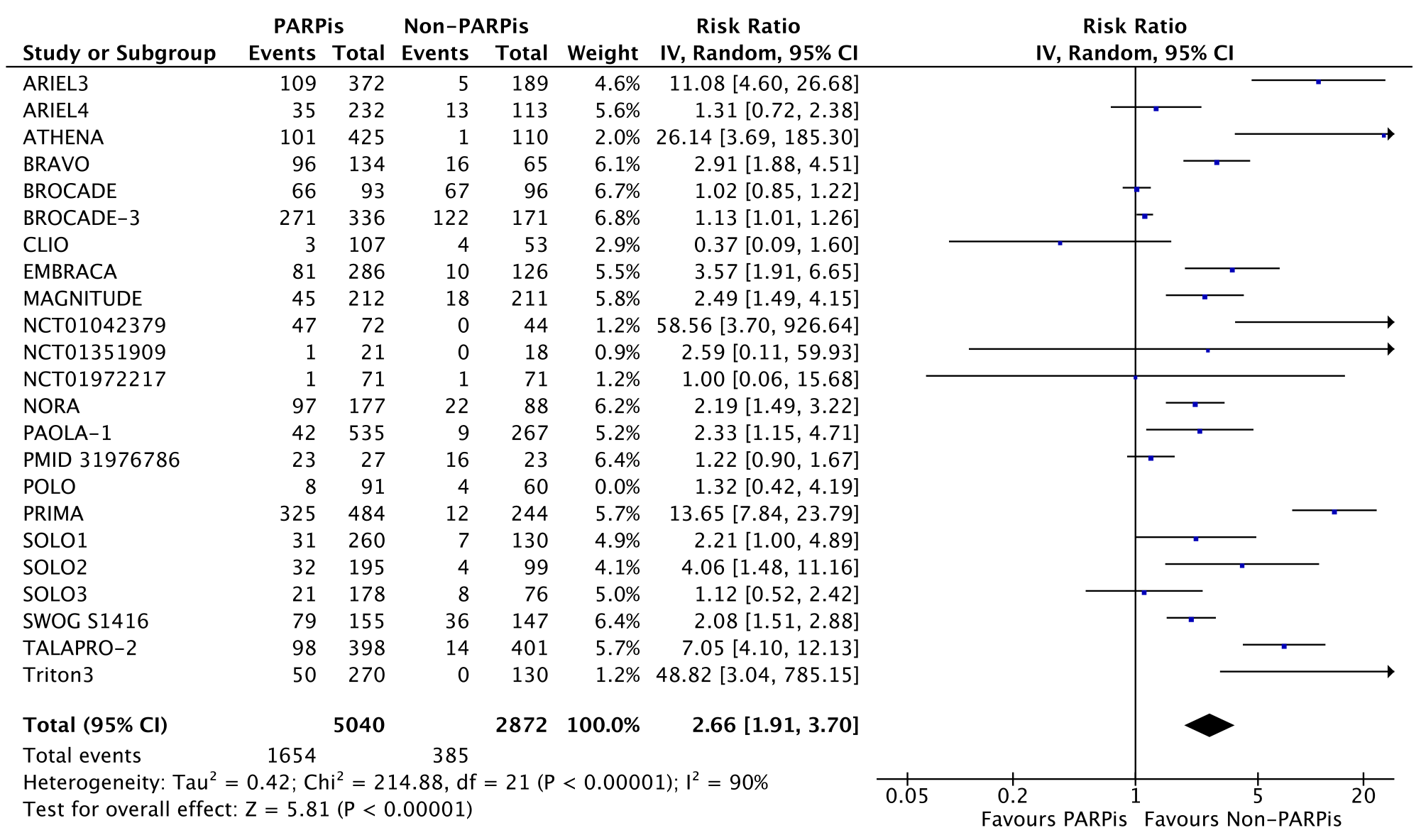 |
| 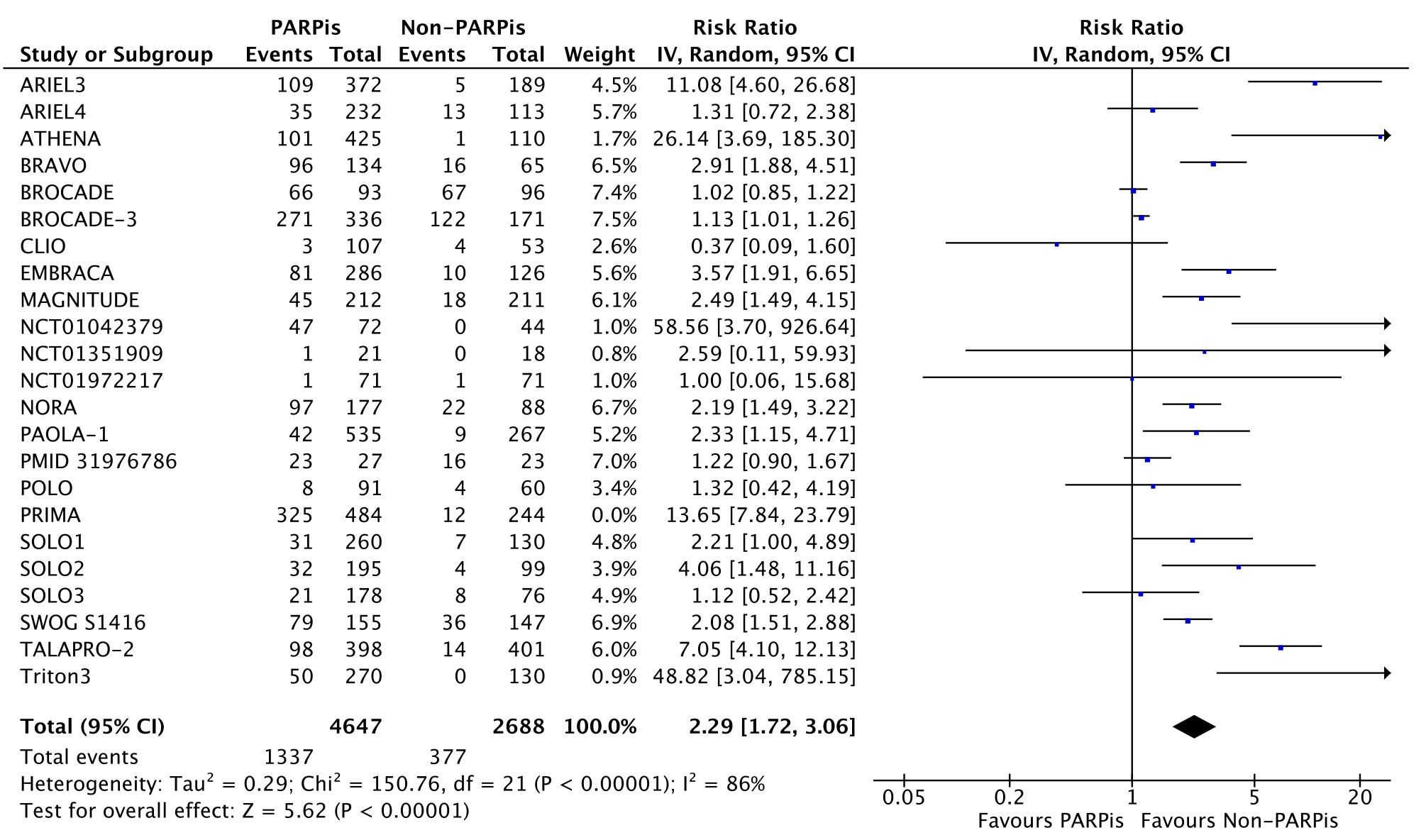 | 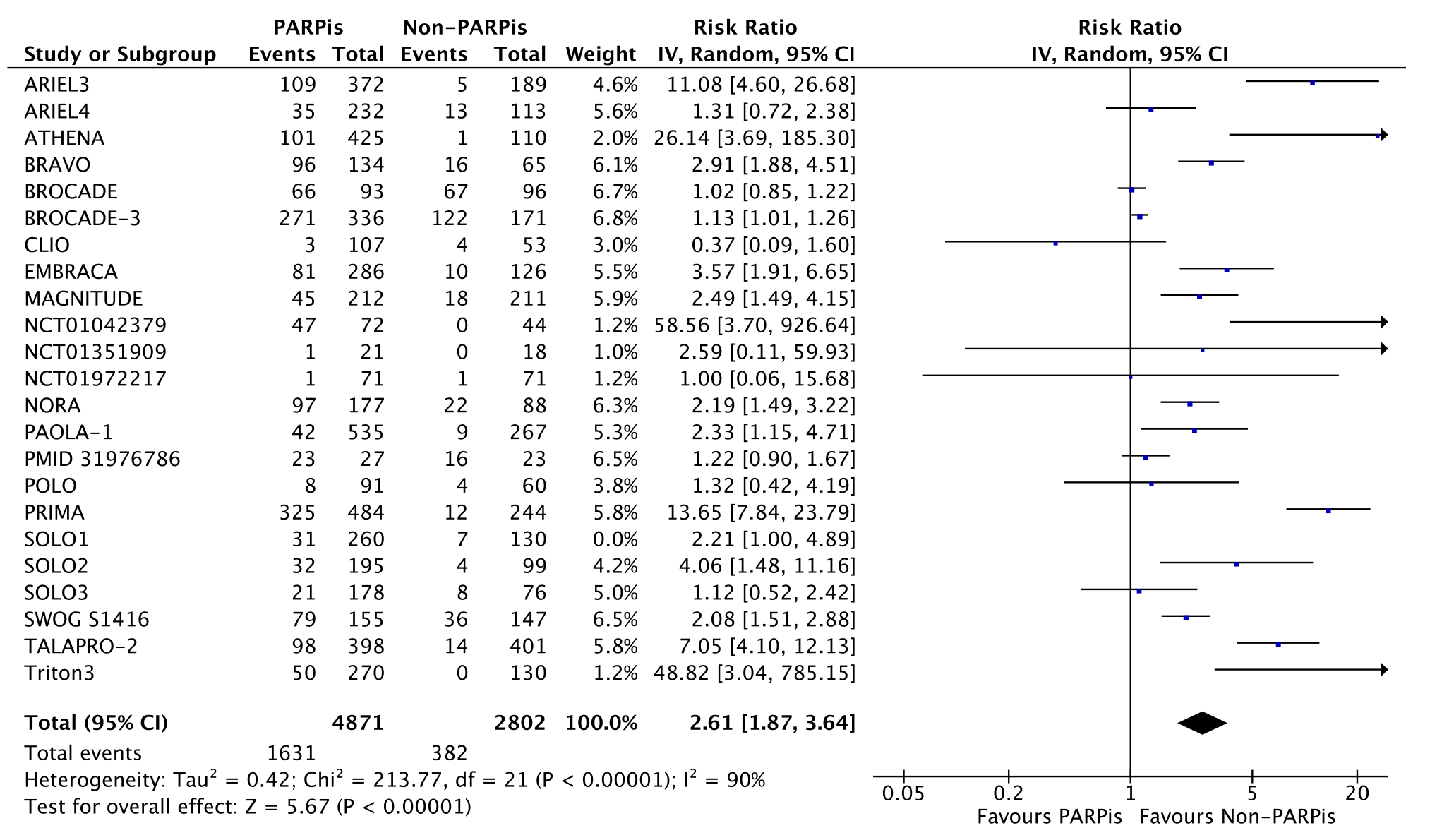 |
| 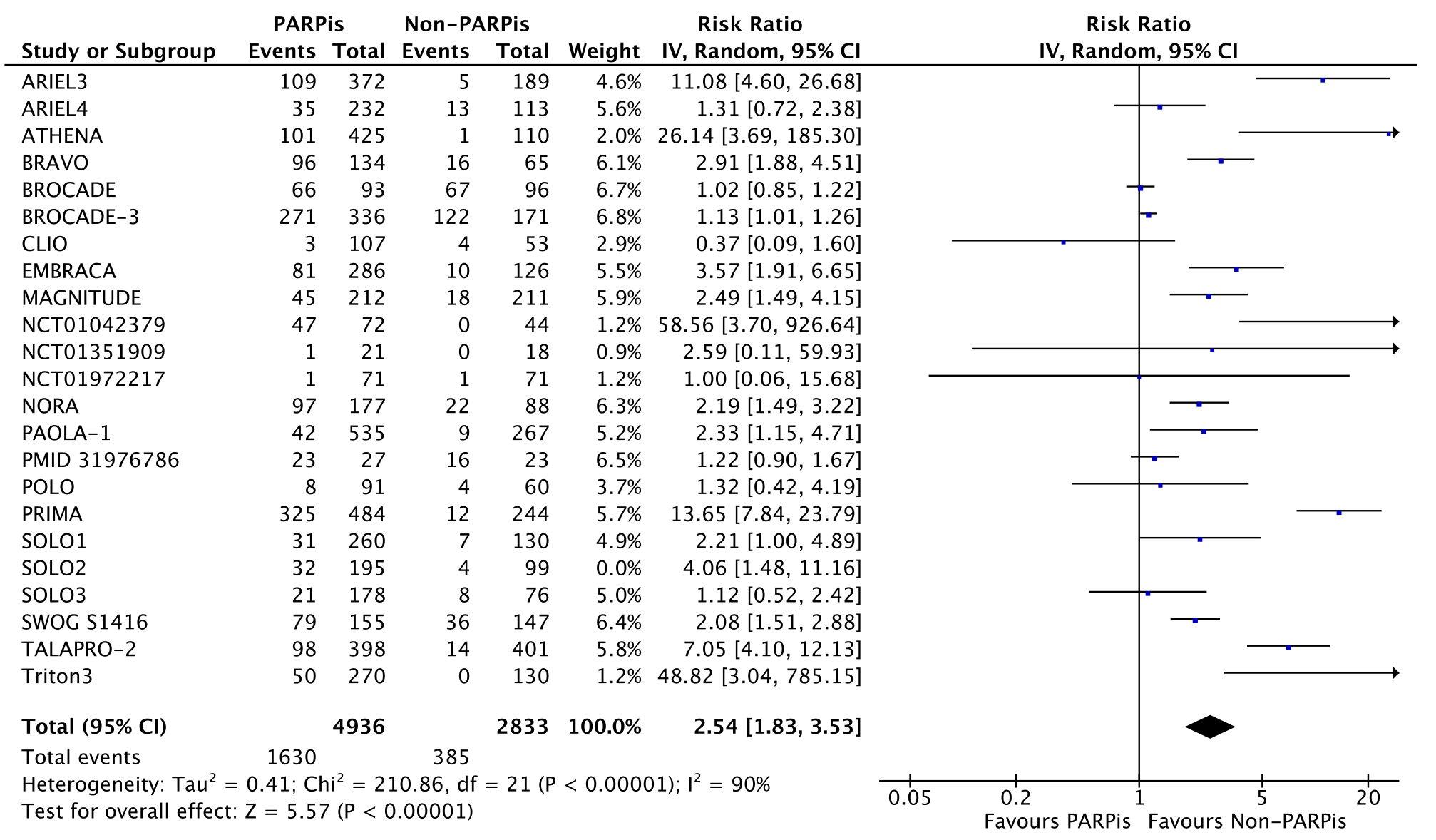 | 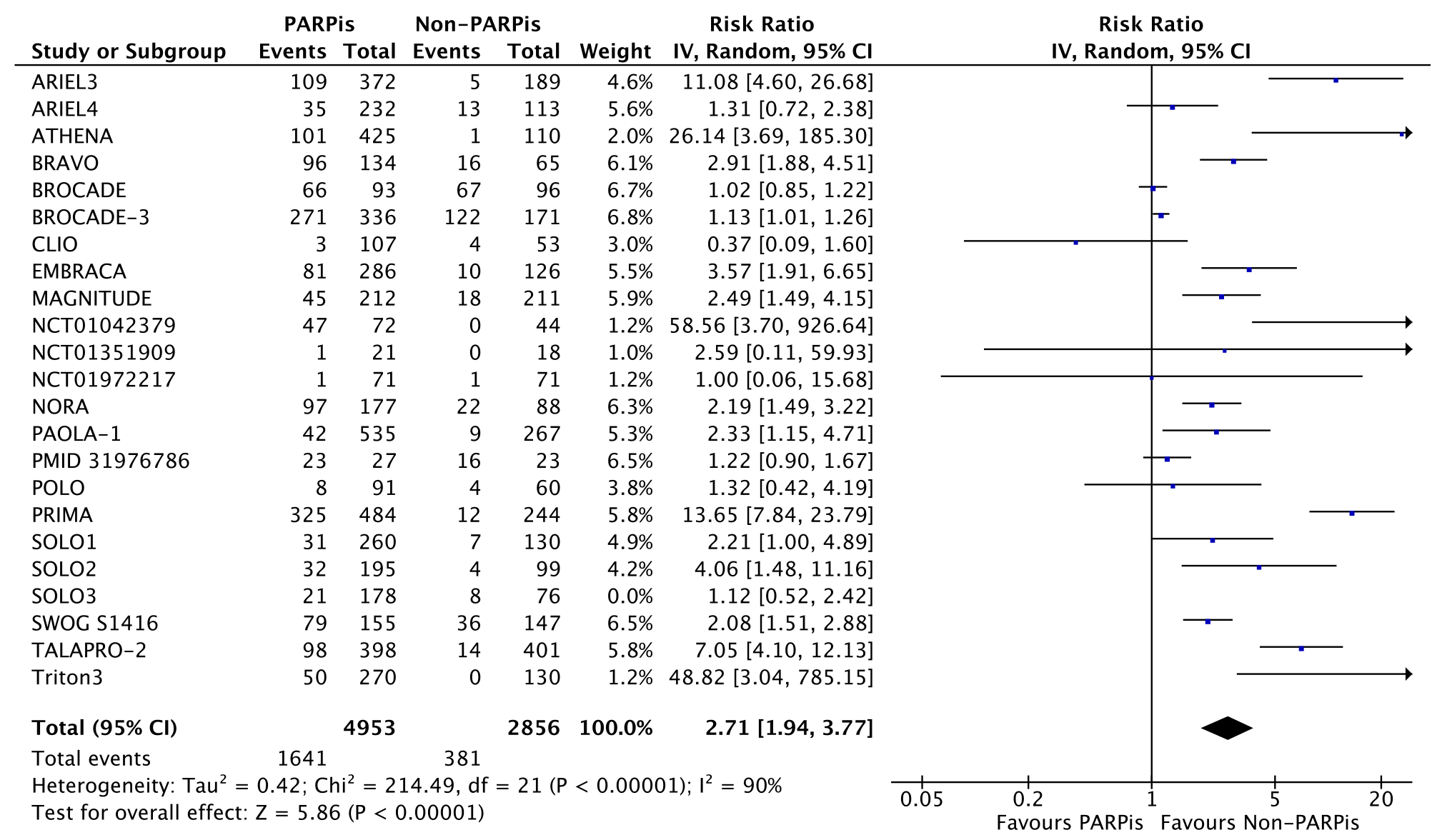 |
| 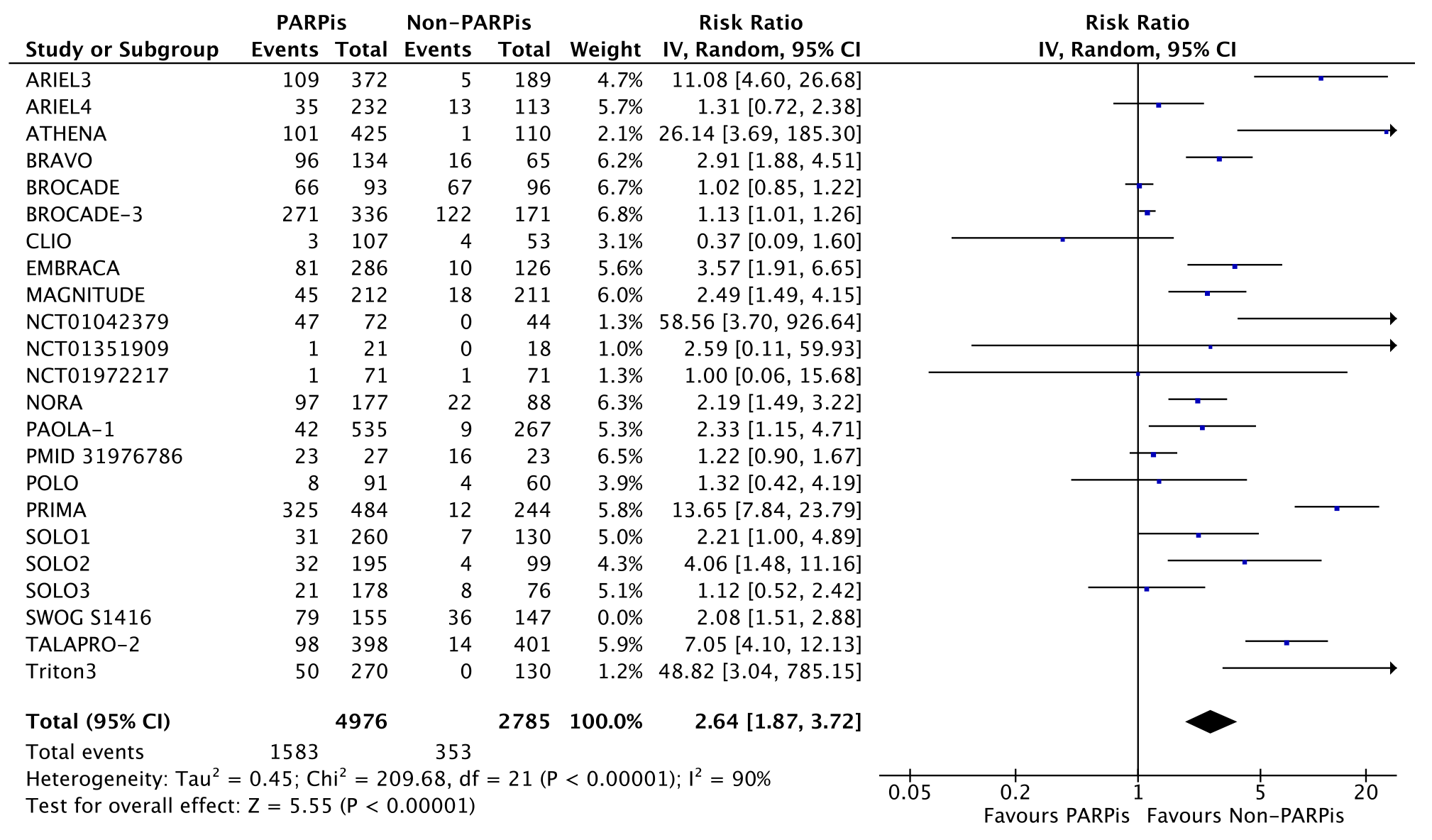 | 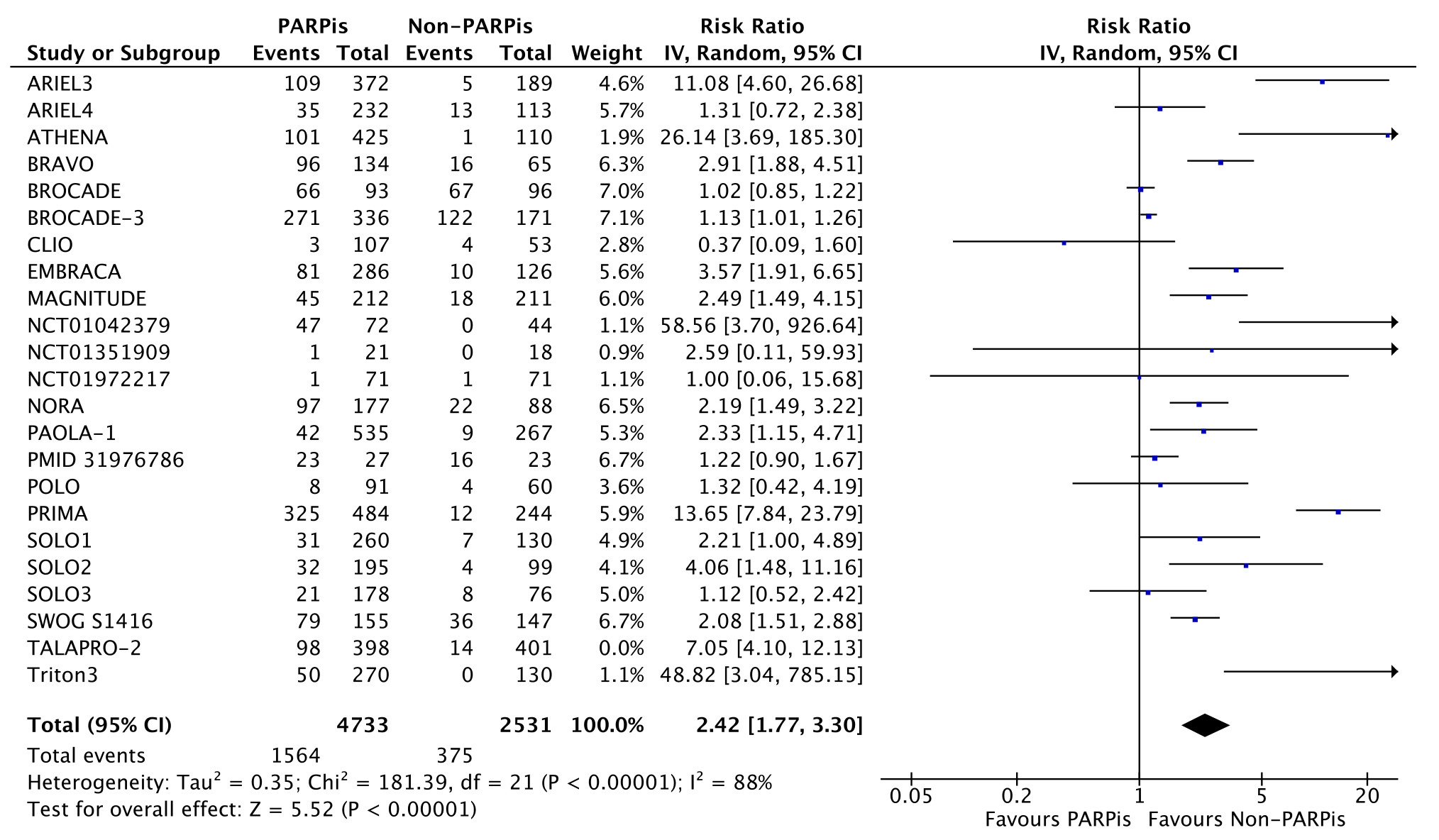 |
| 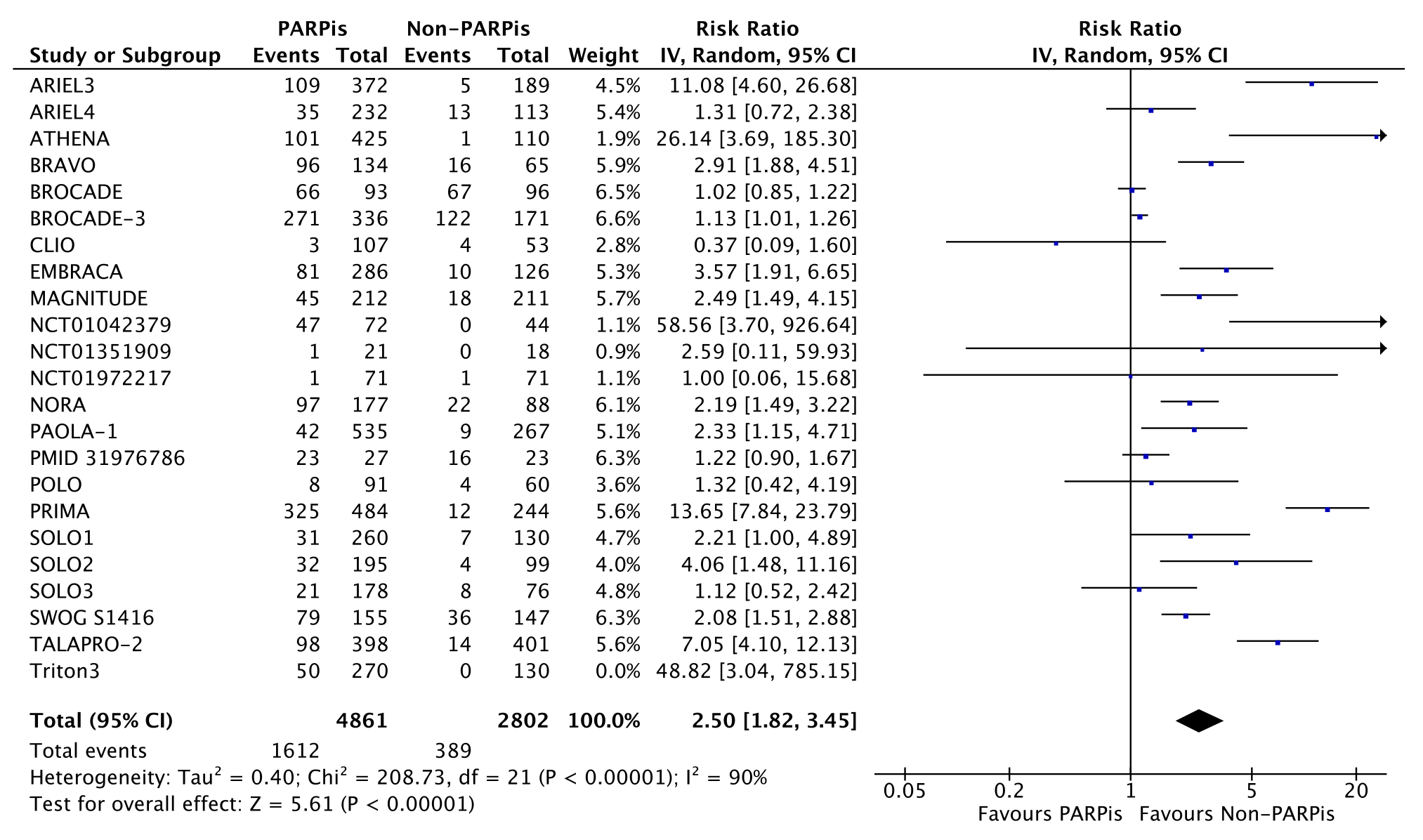 |  |

D

| 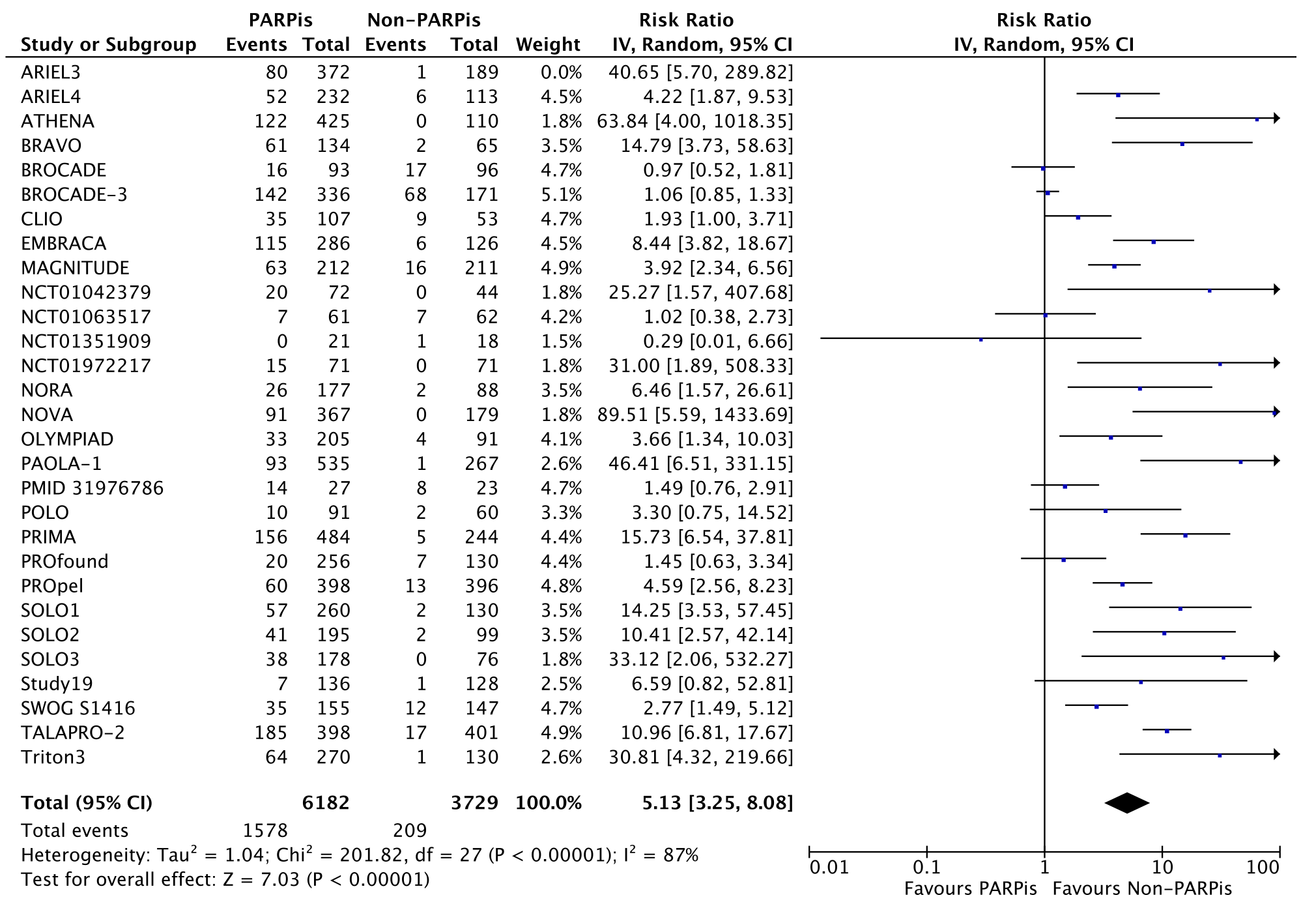 | 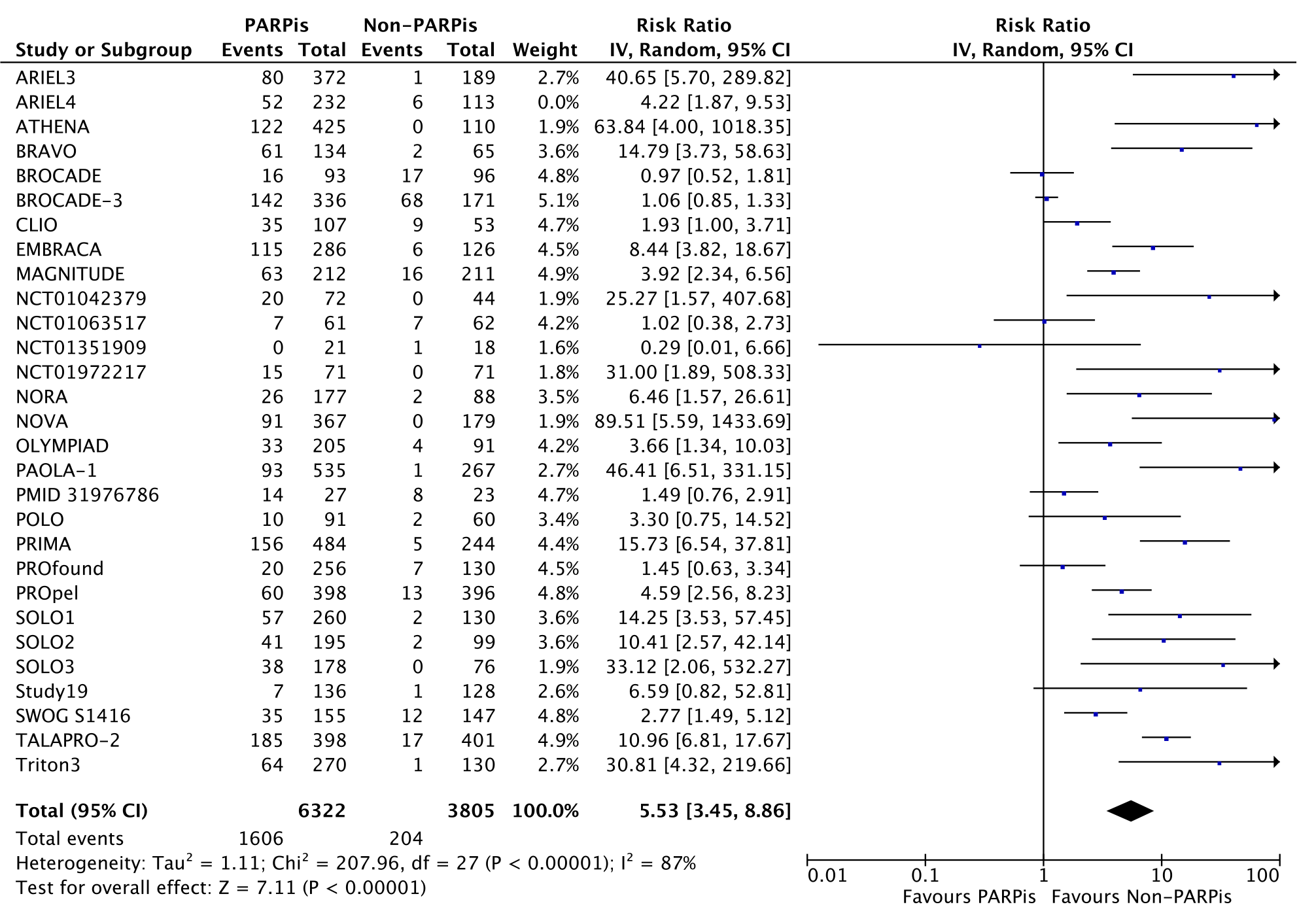 |
| --- | --- |
| 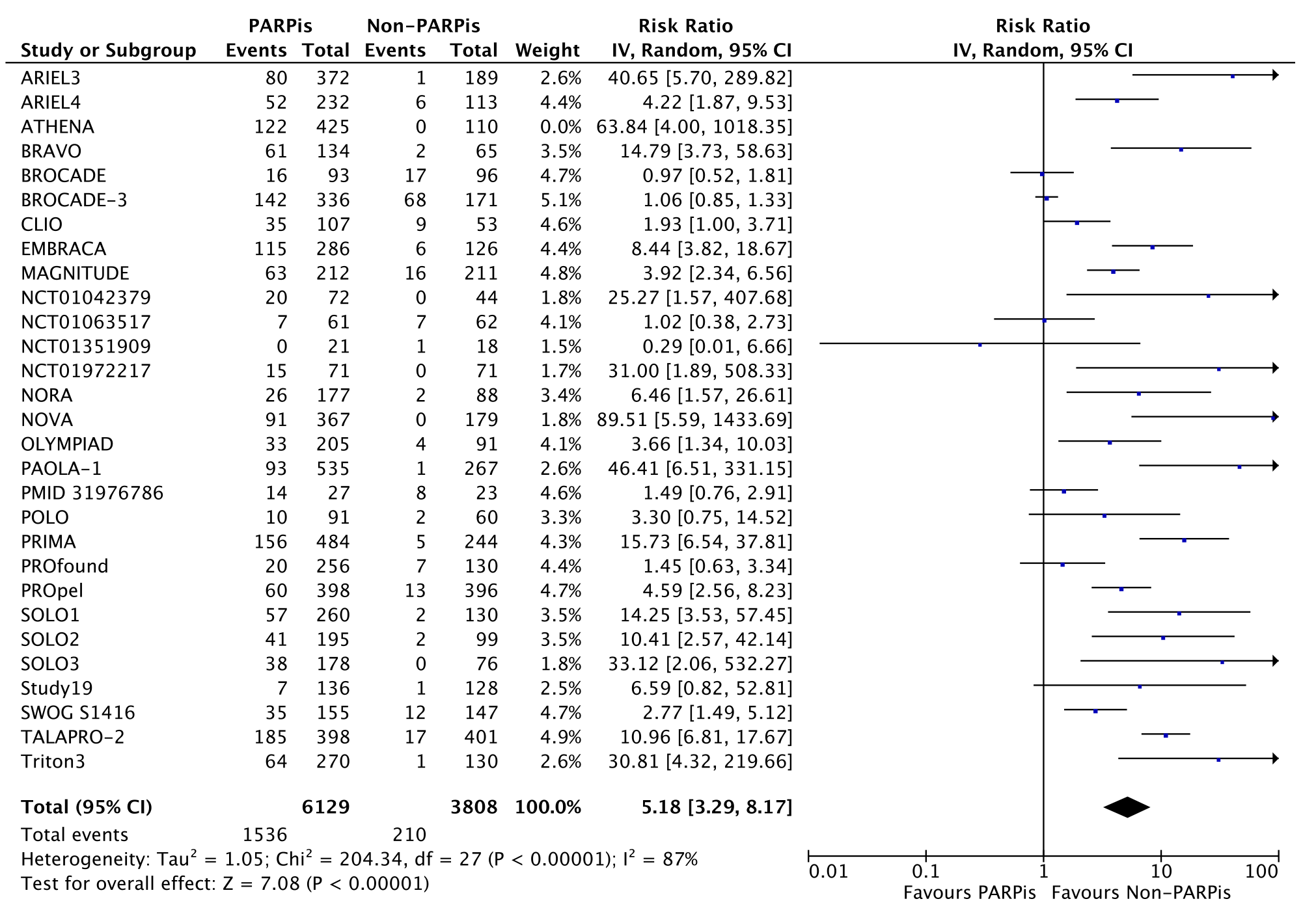 | 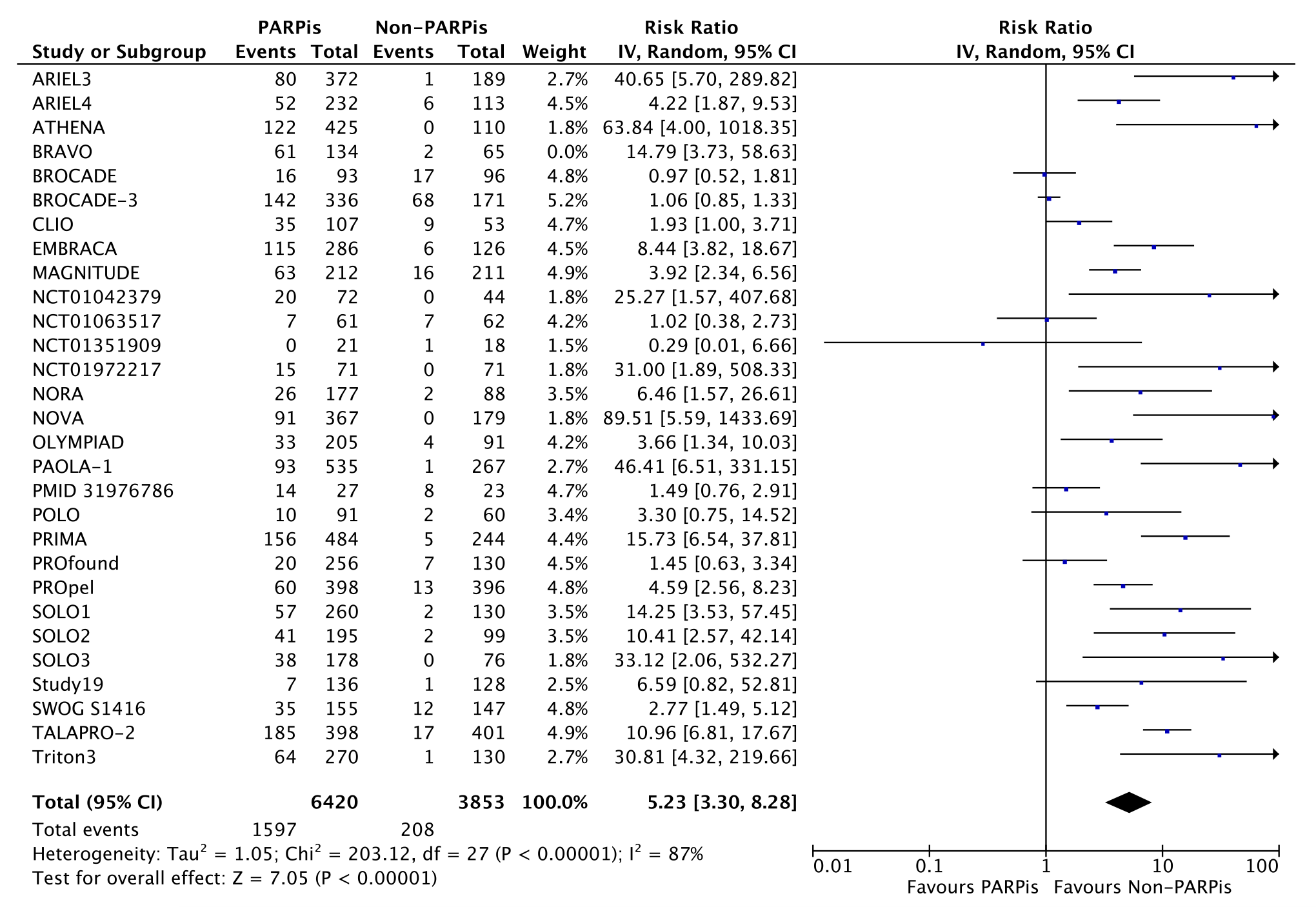 |
| 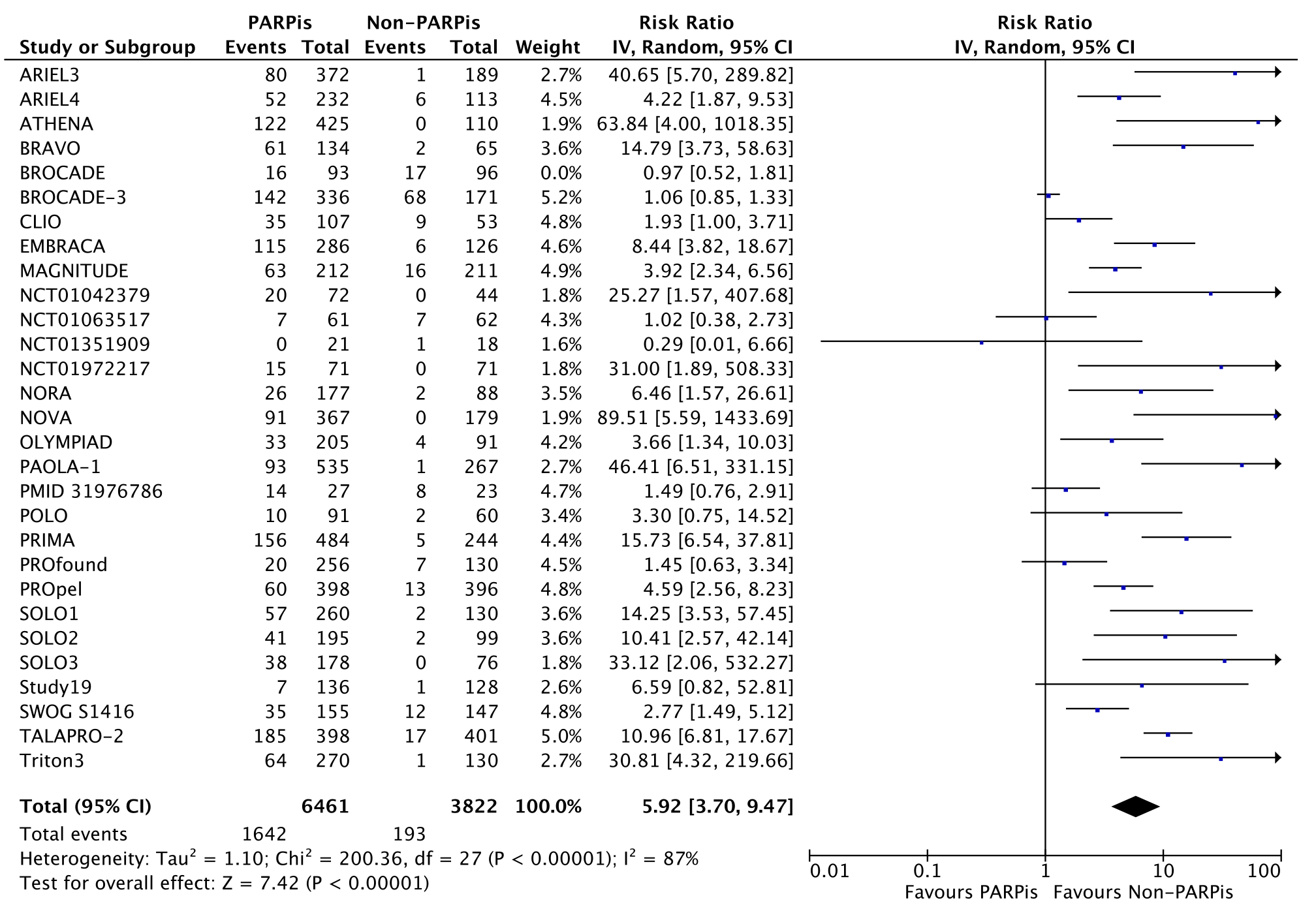 | 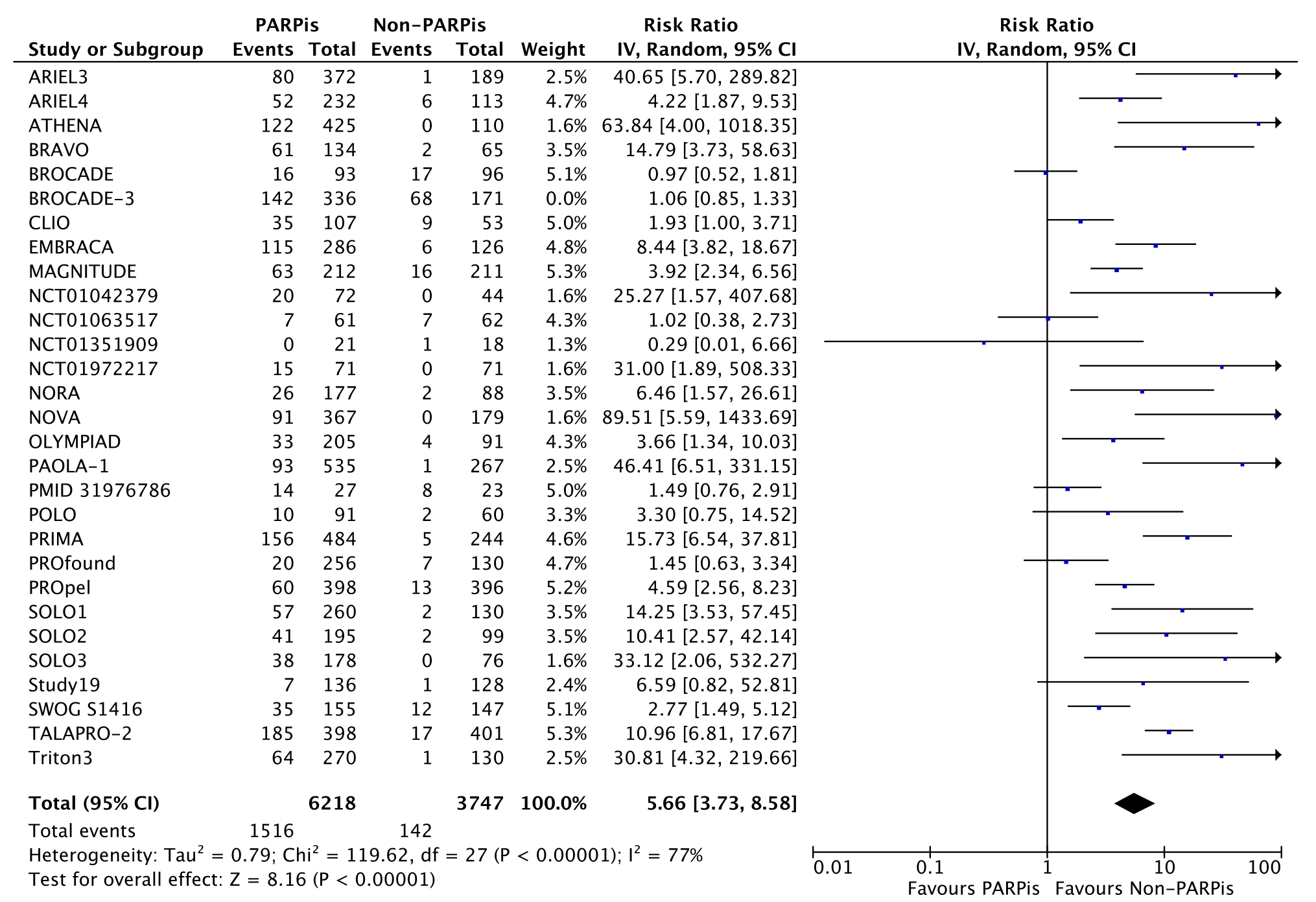 |
| 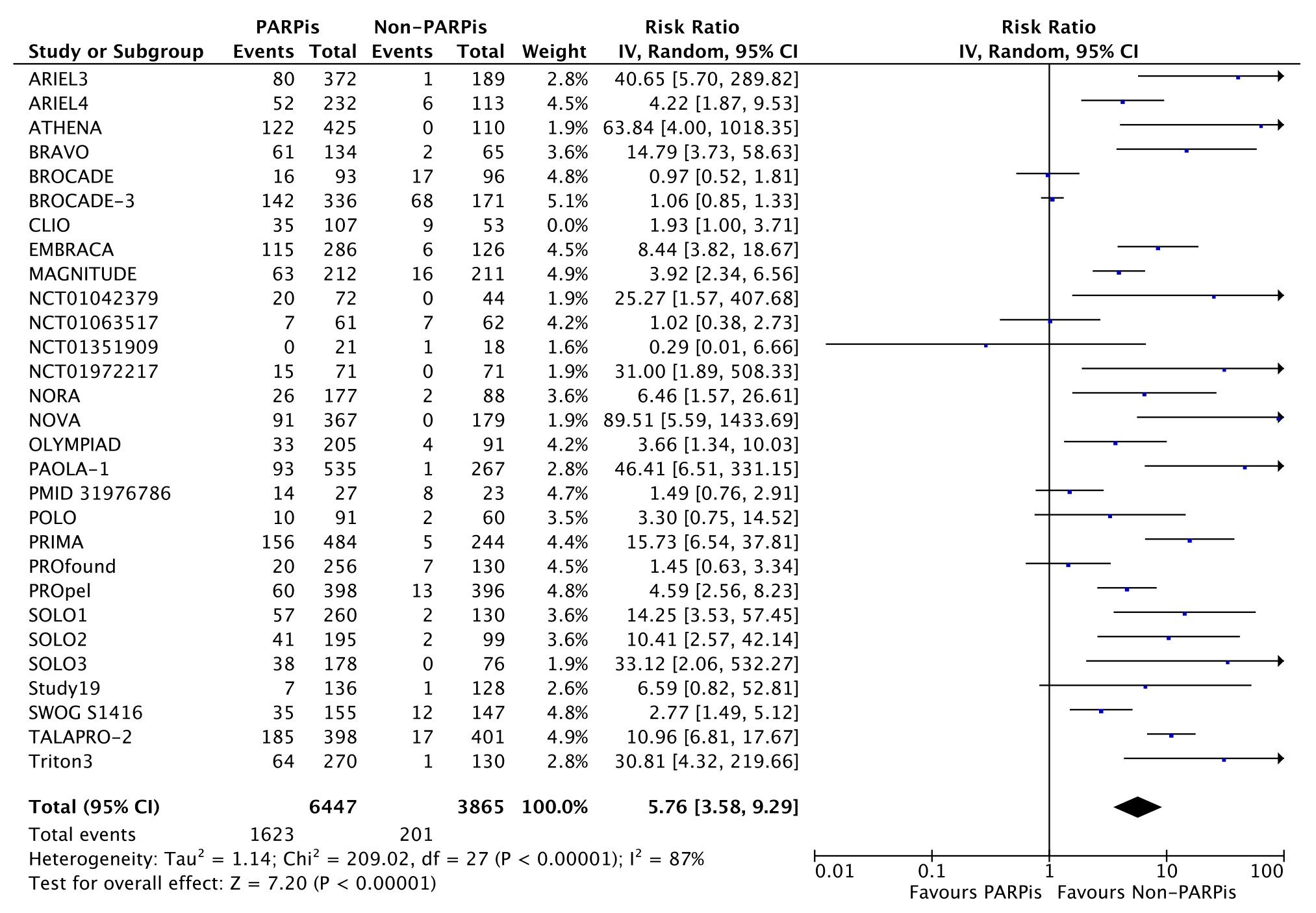 | 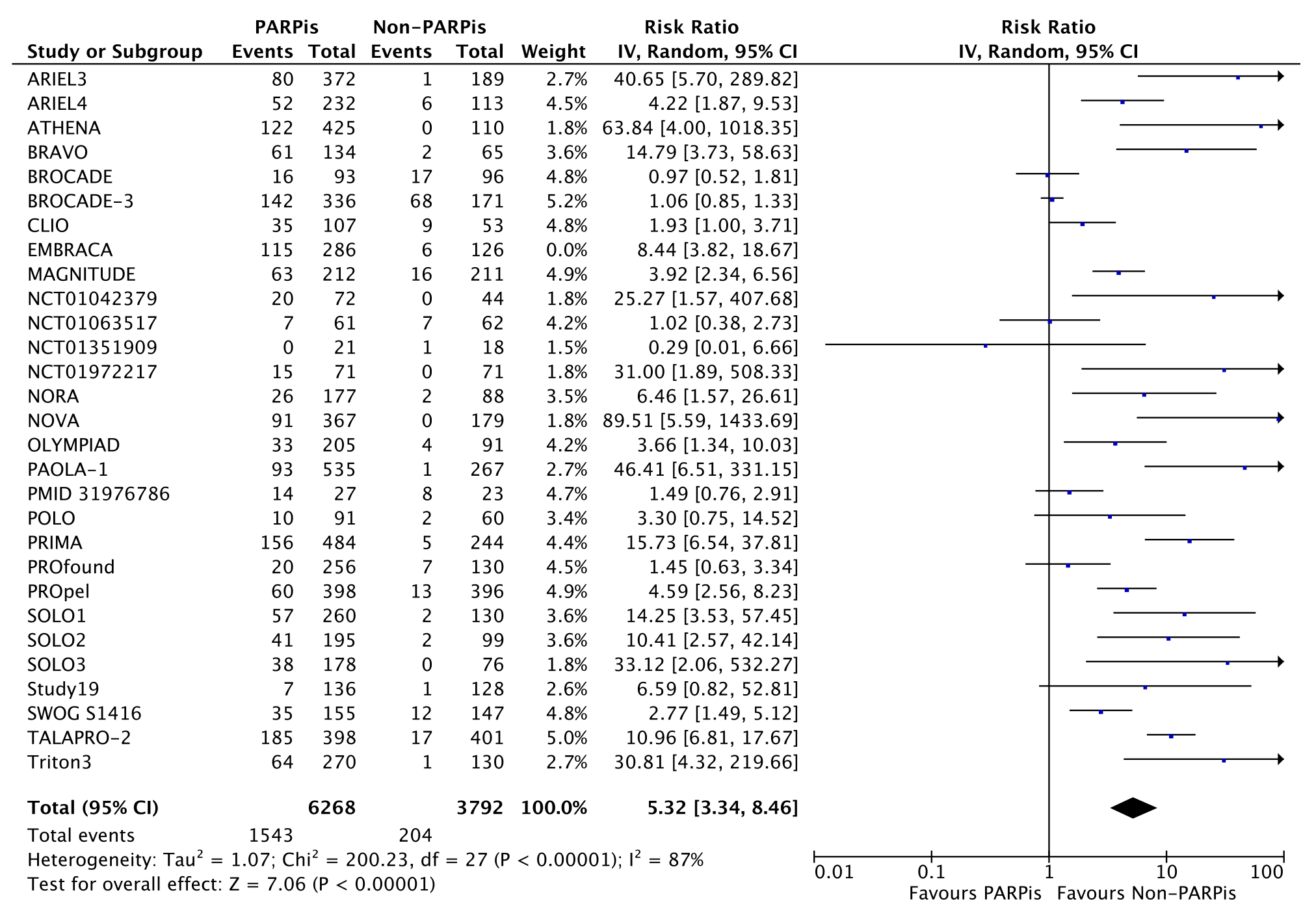 |
| 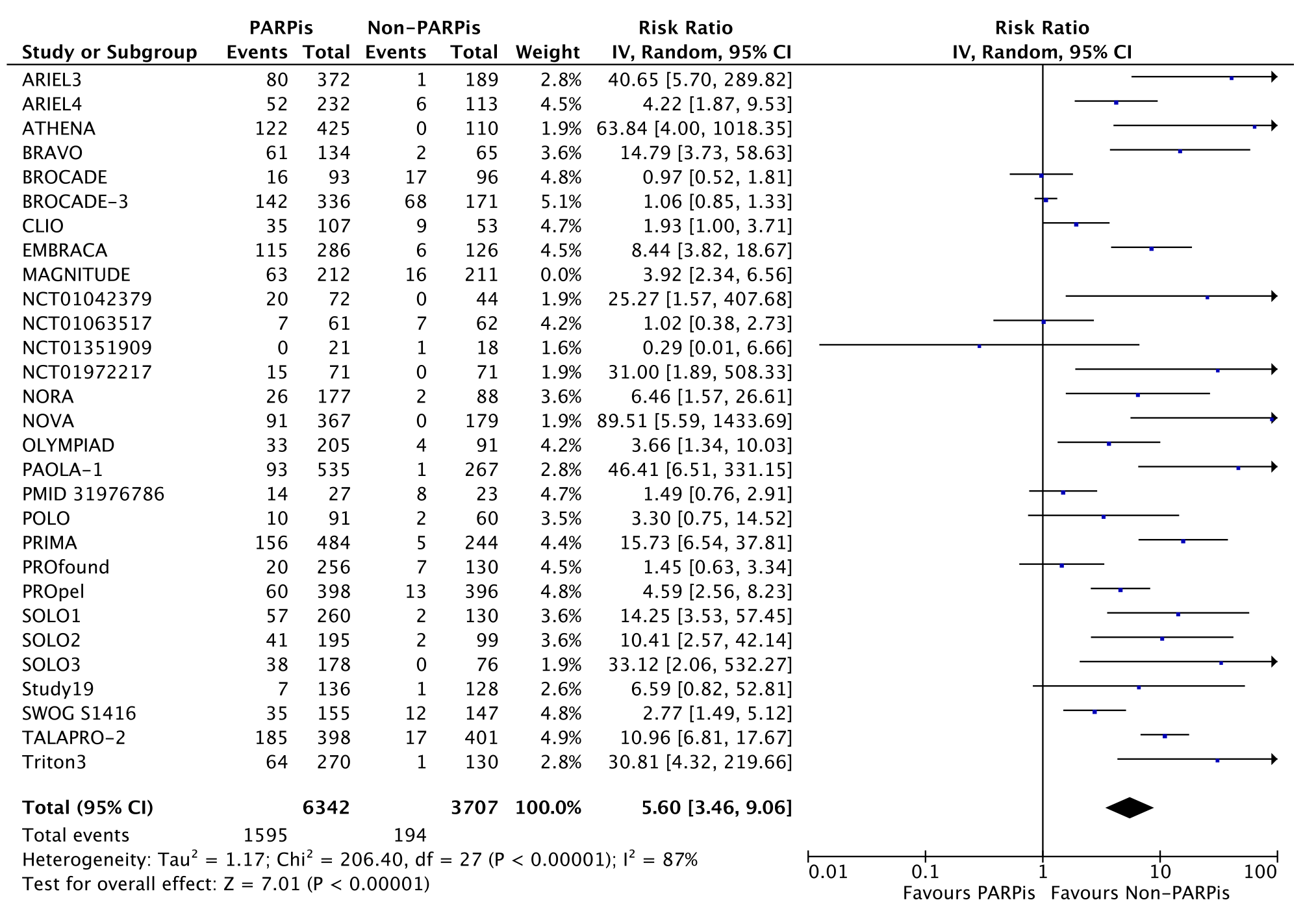 | 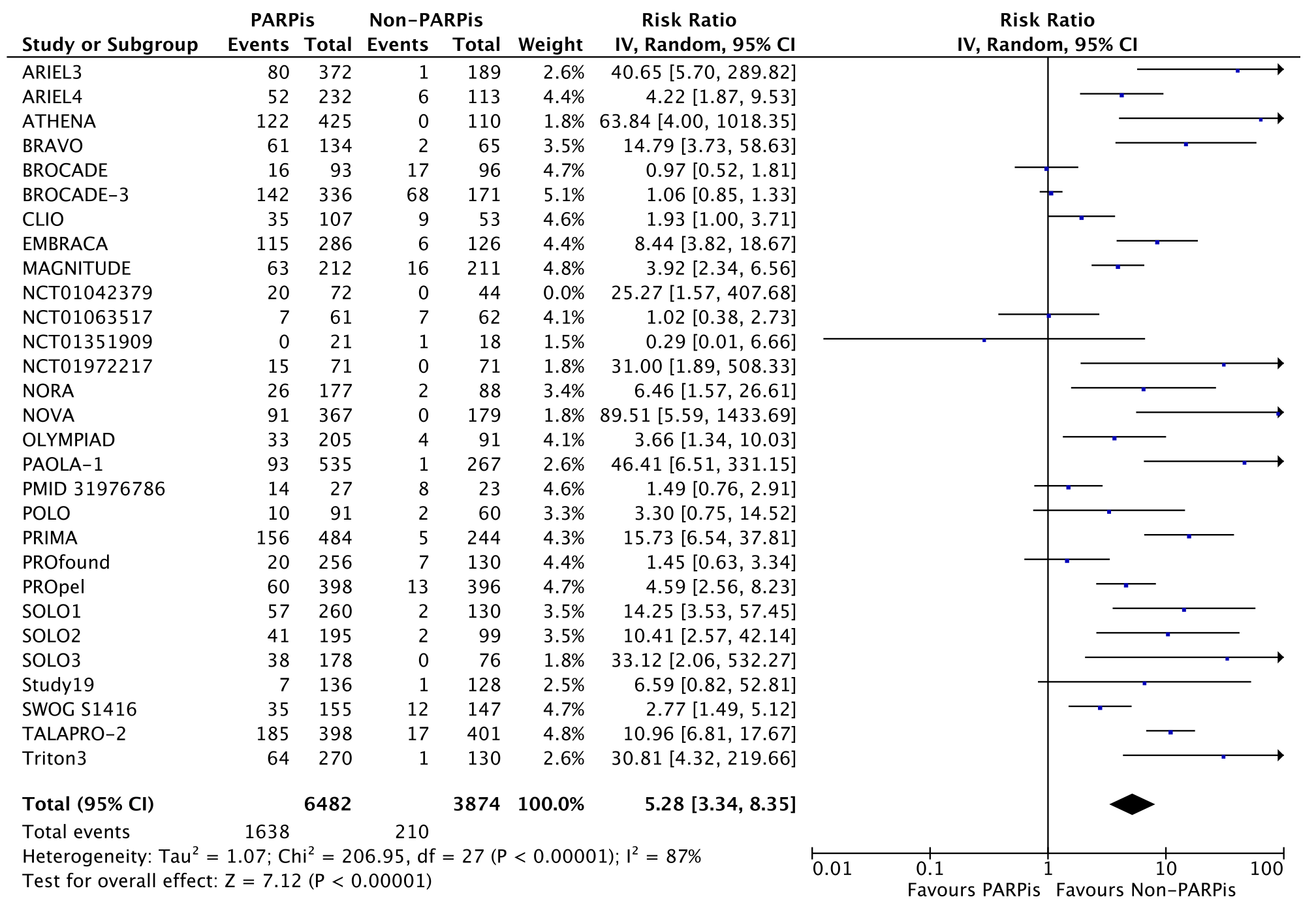 |
| 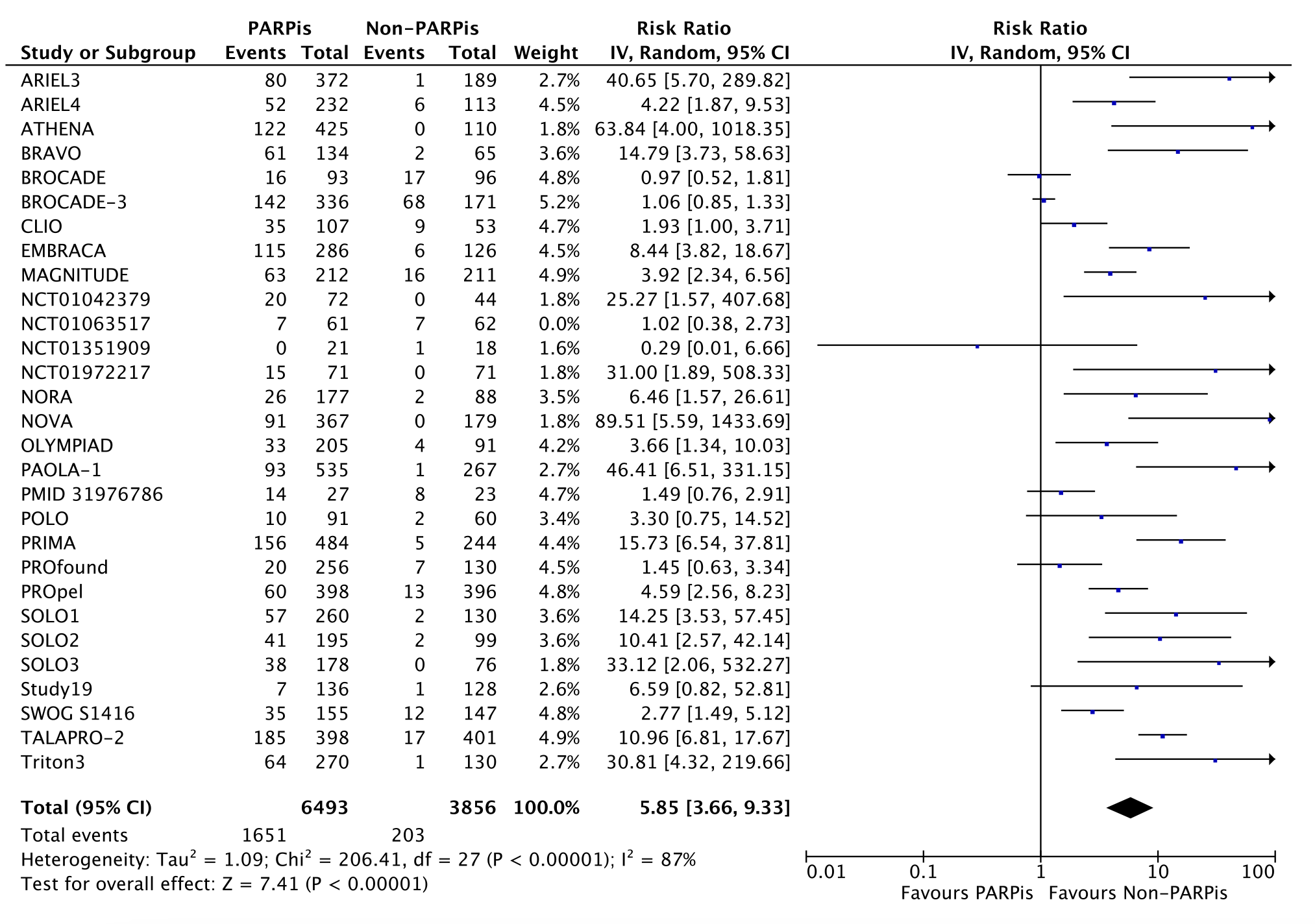 | 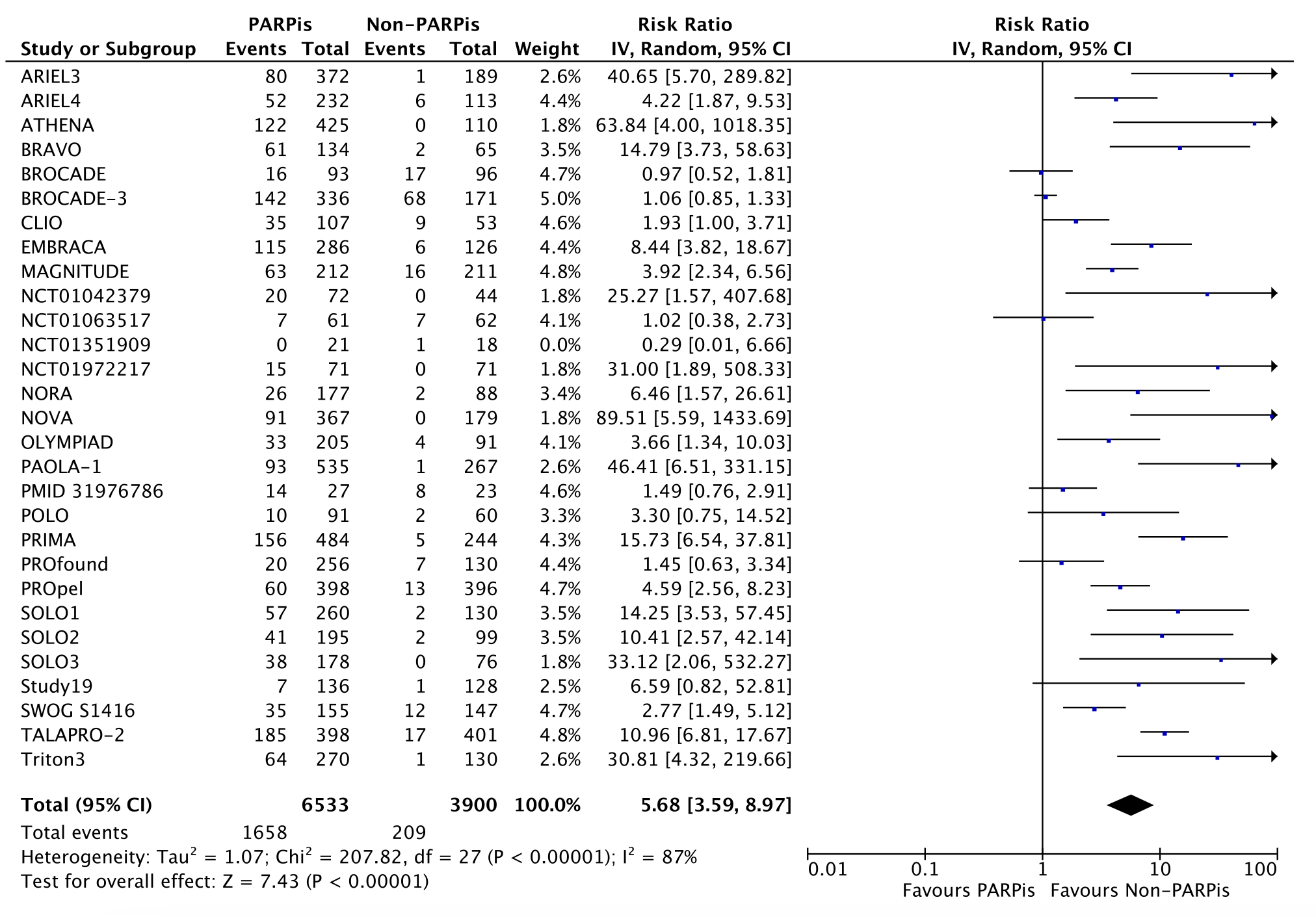 |
| 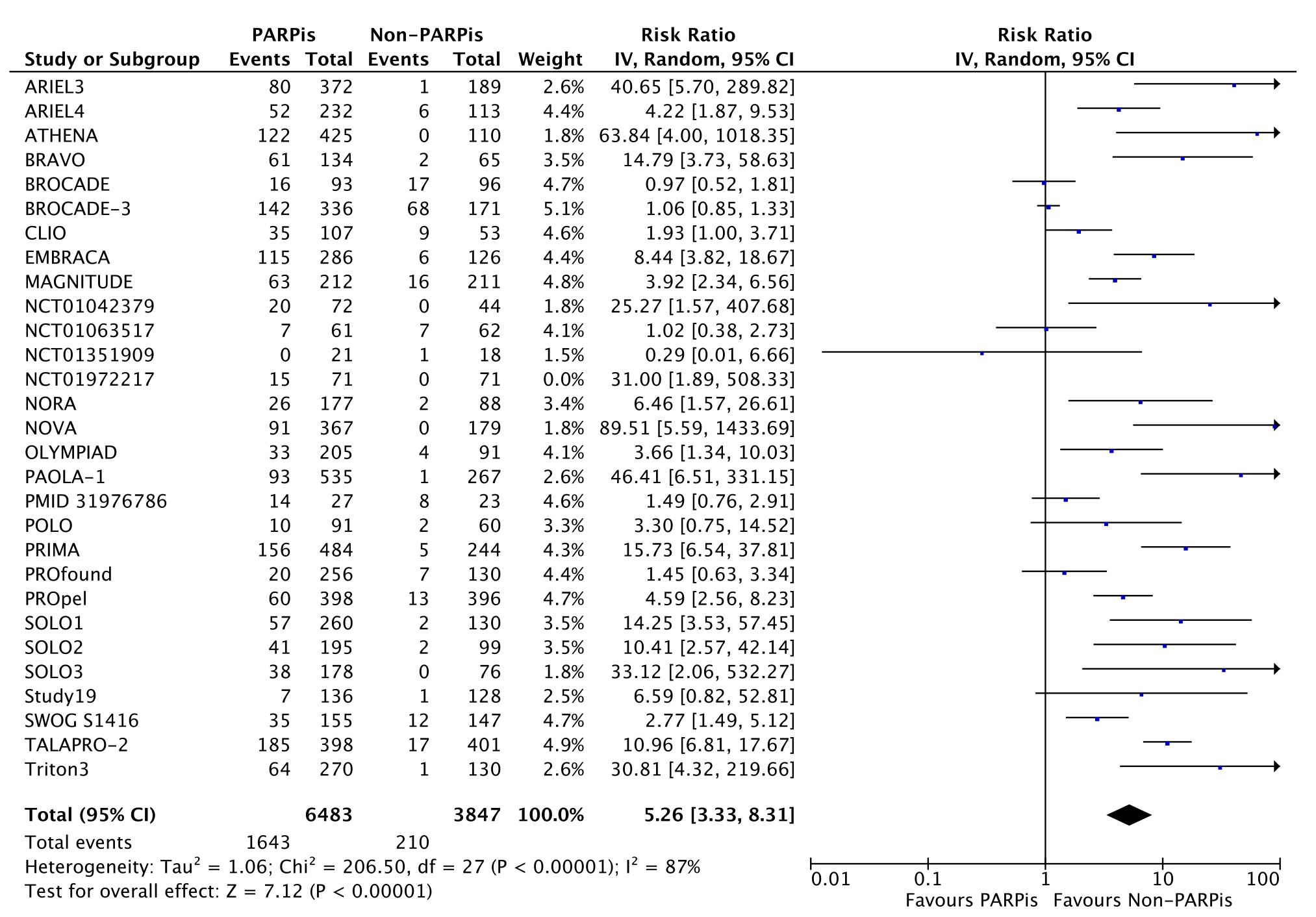 | 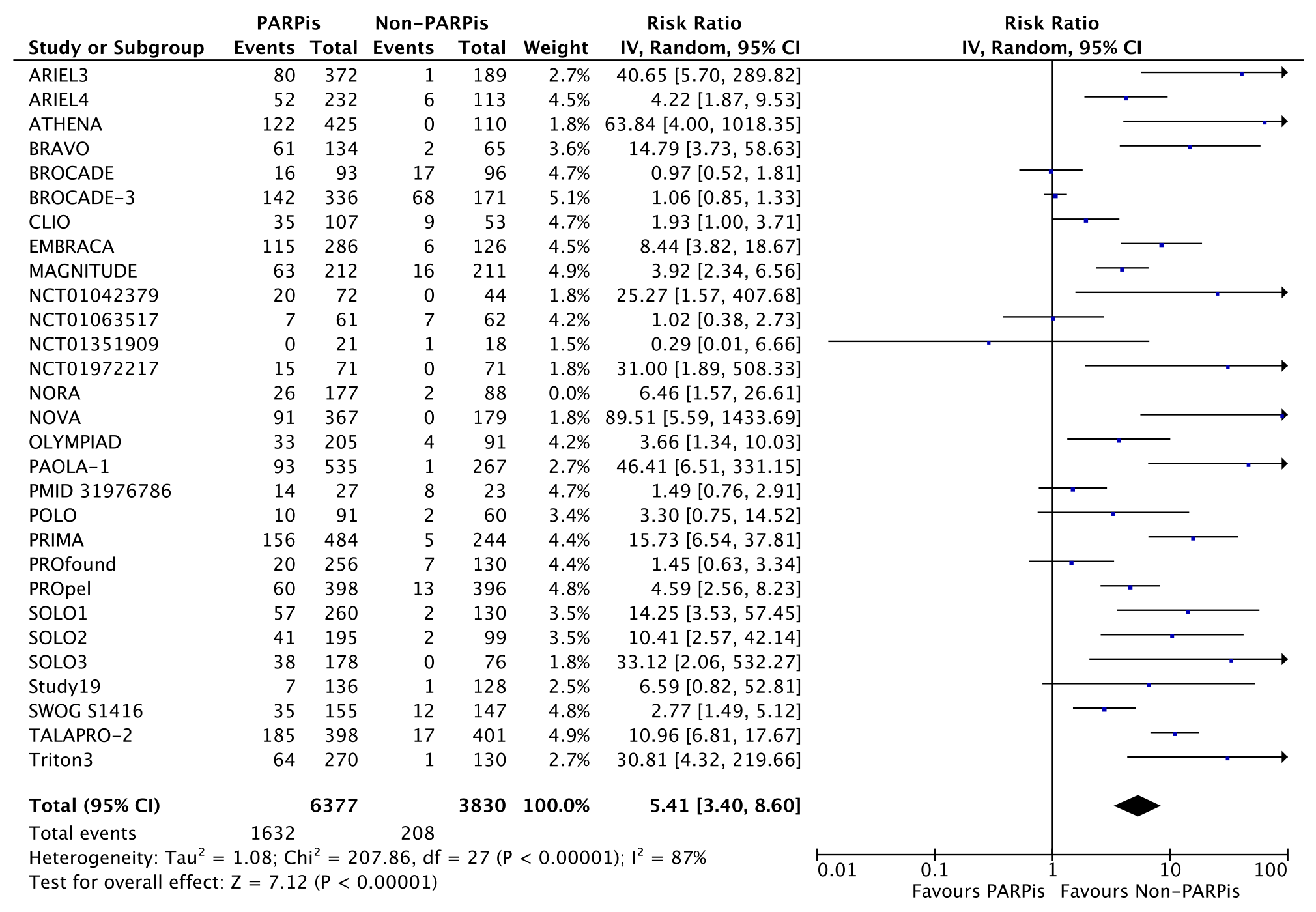 |
| 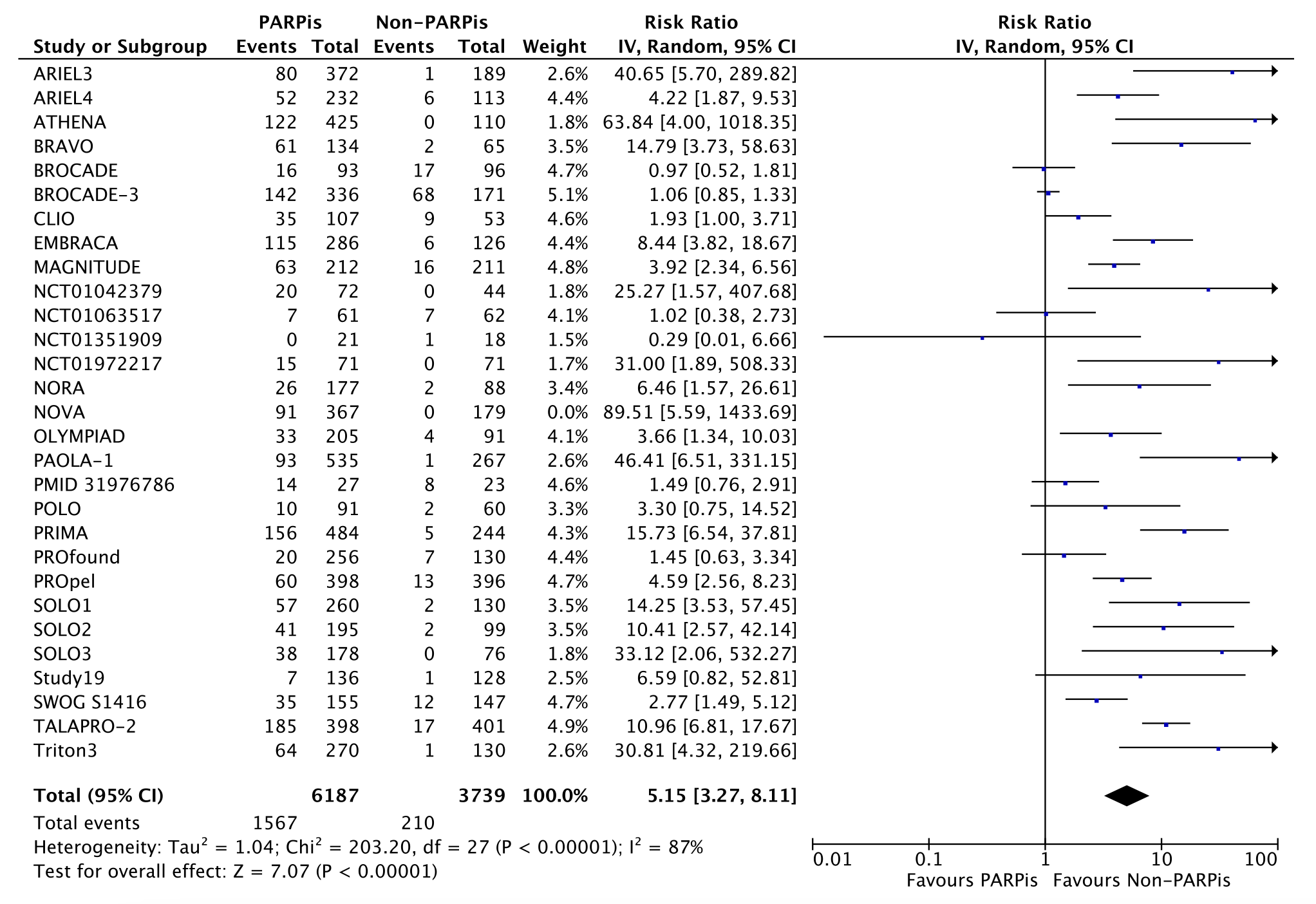 | 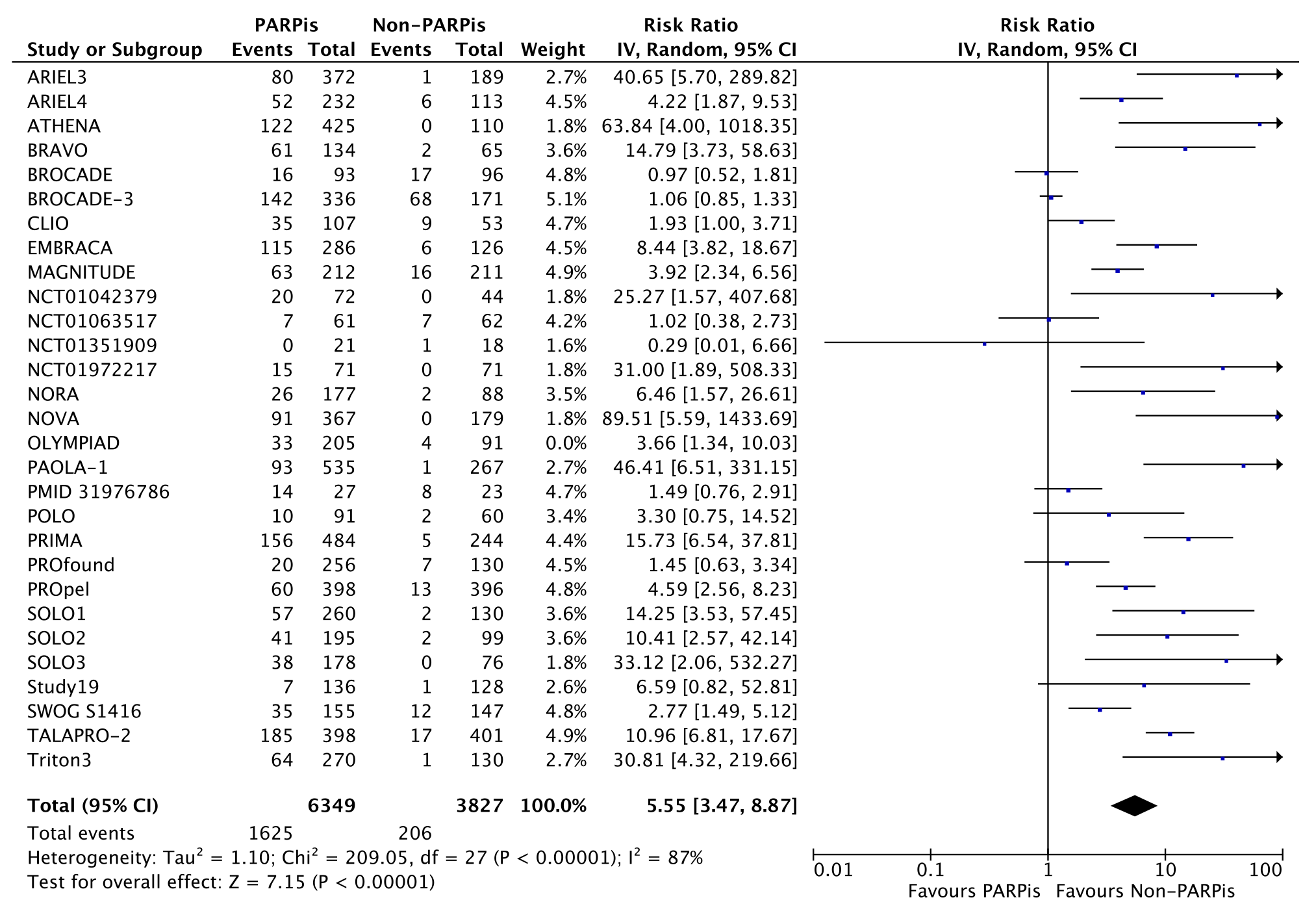 |
| 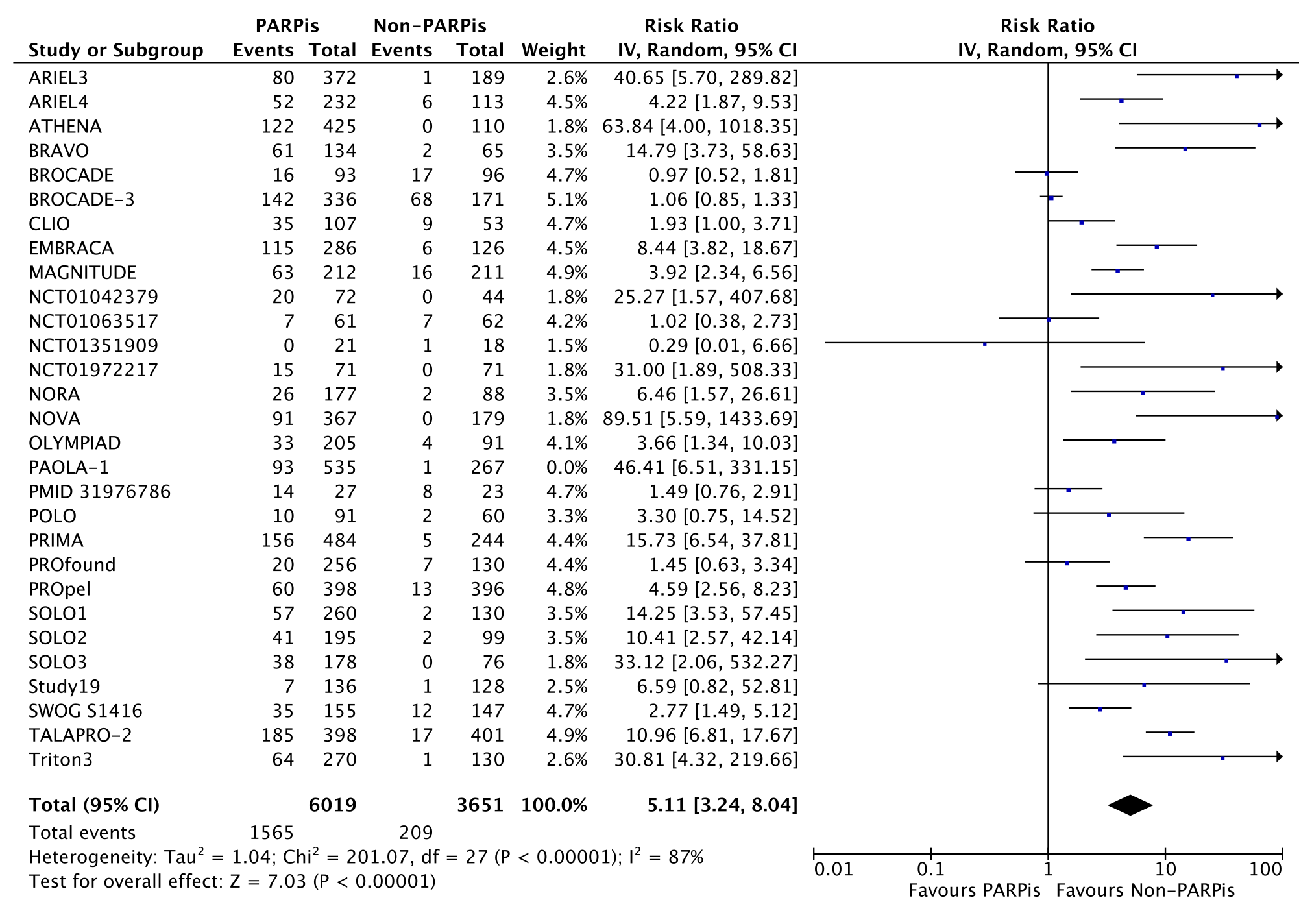 | 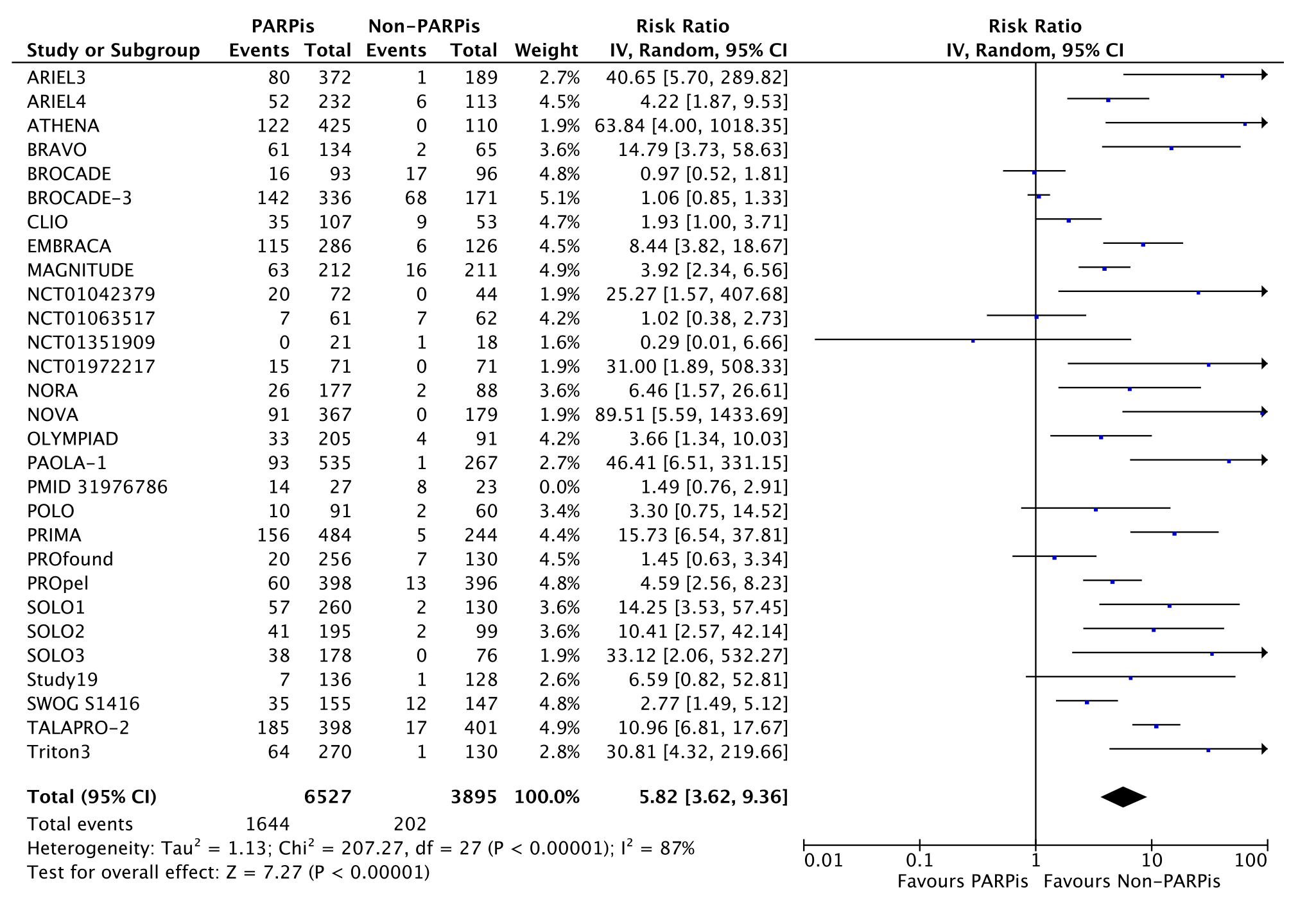 |
| 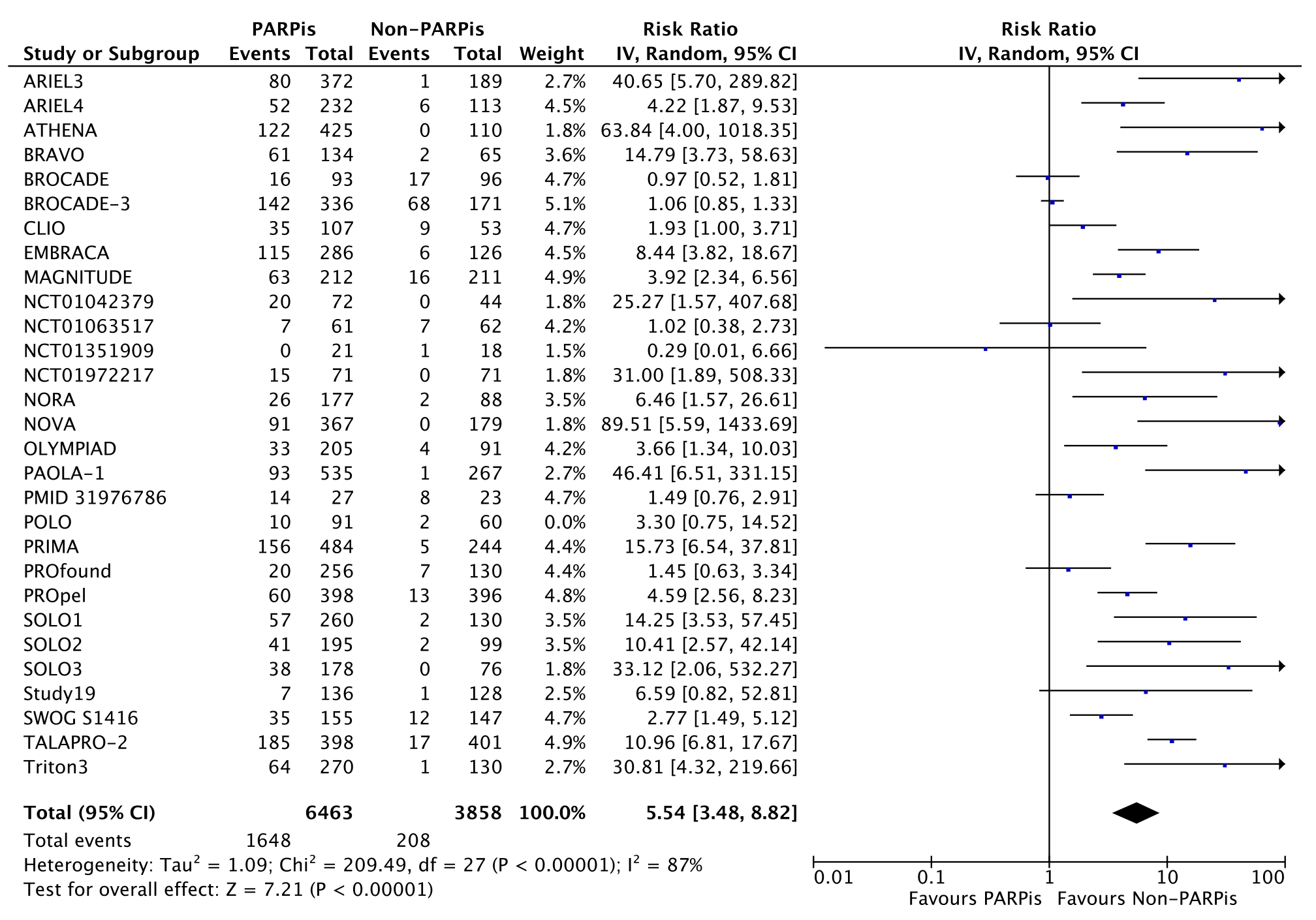 | 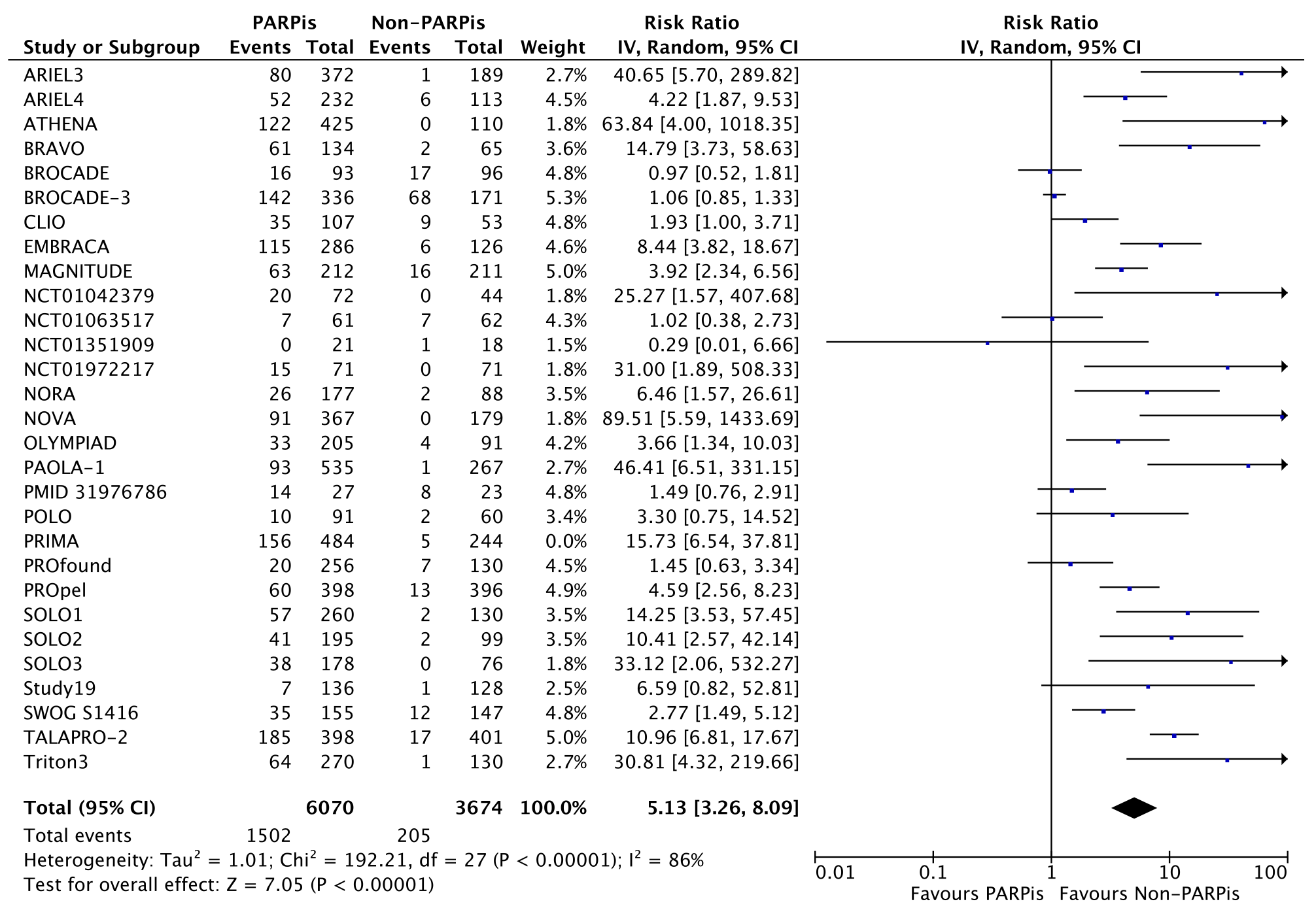 |
| 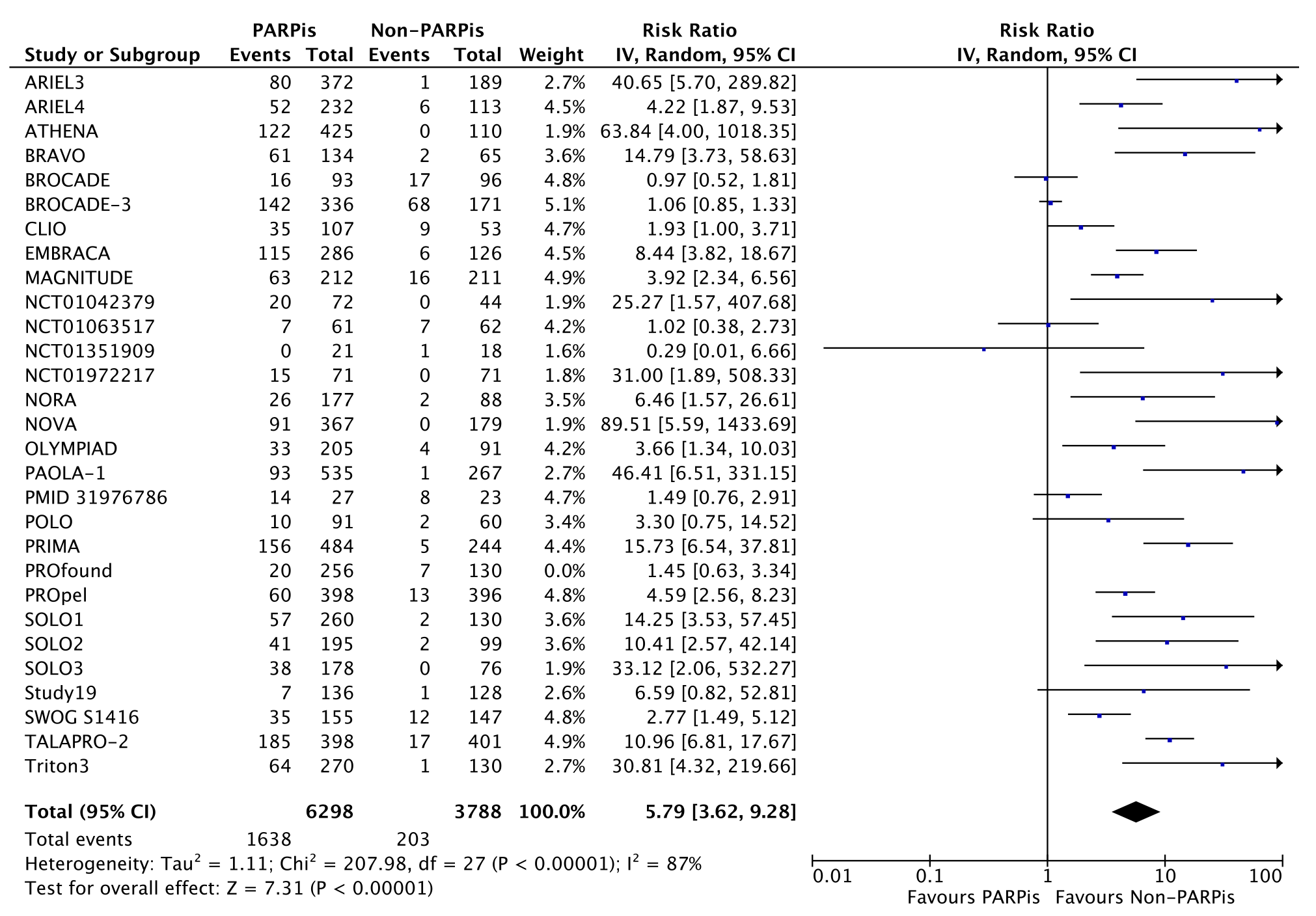 | 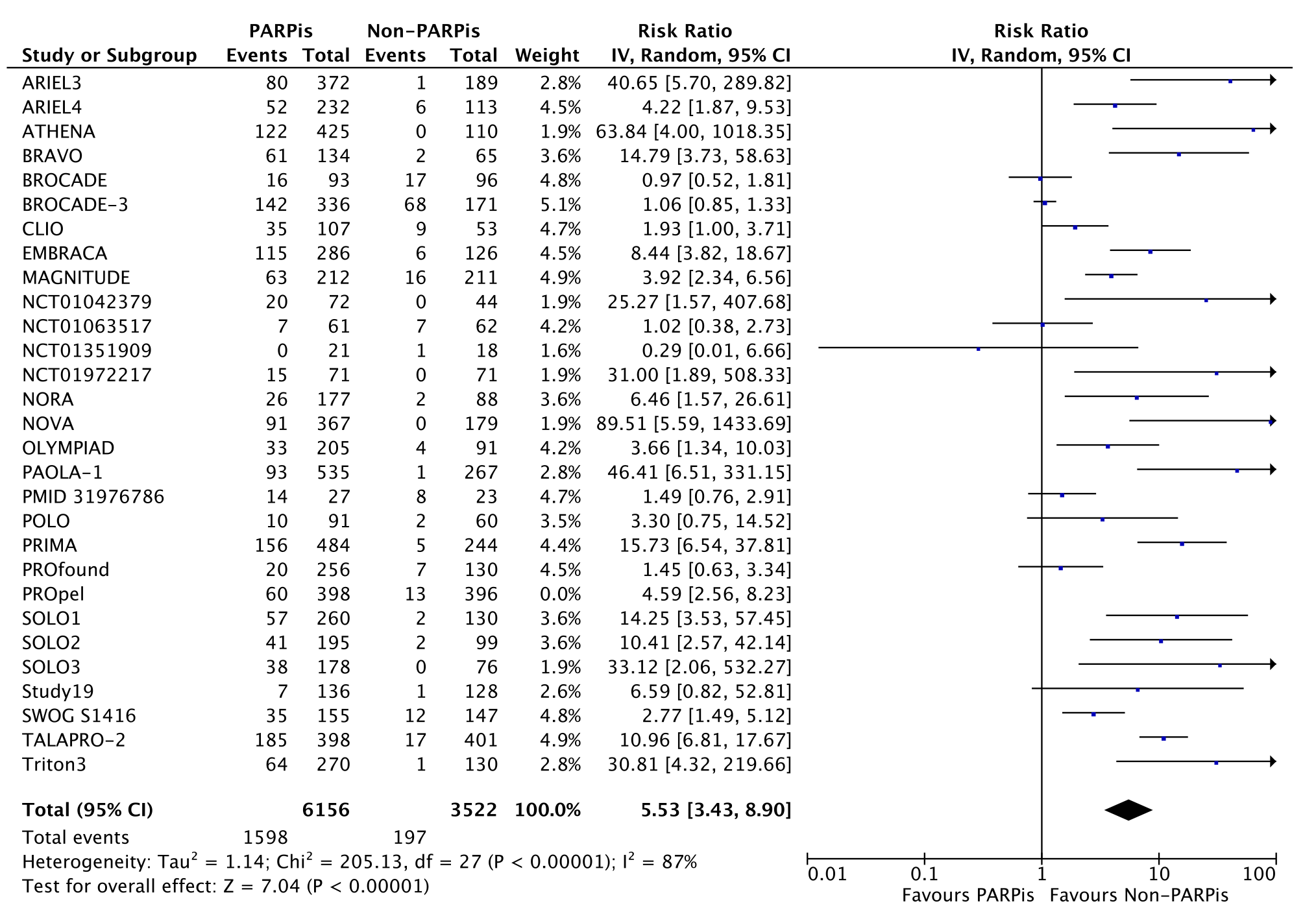 |
| 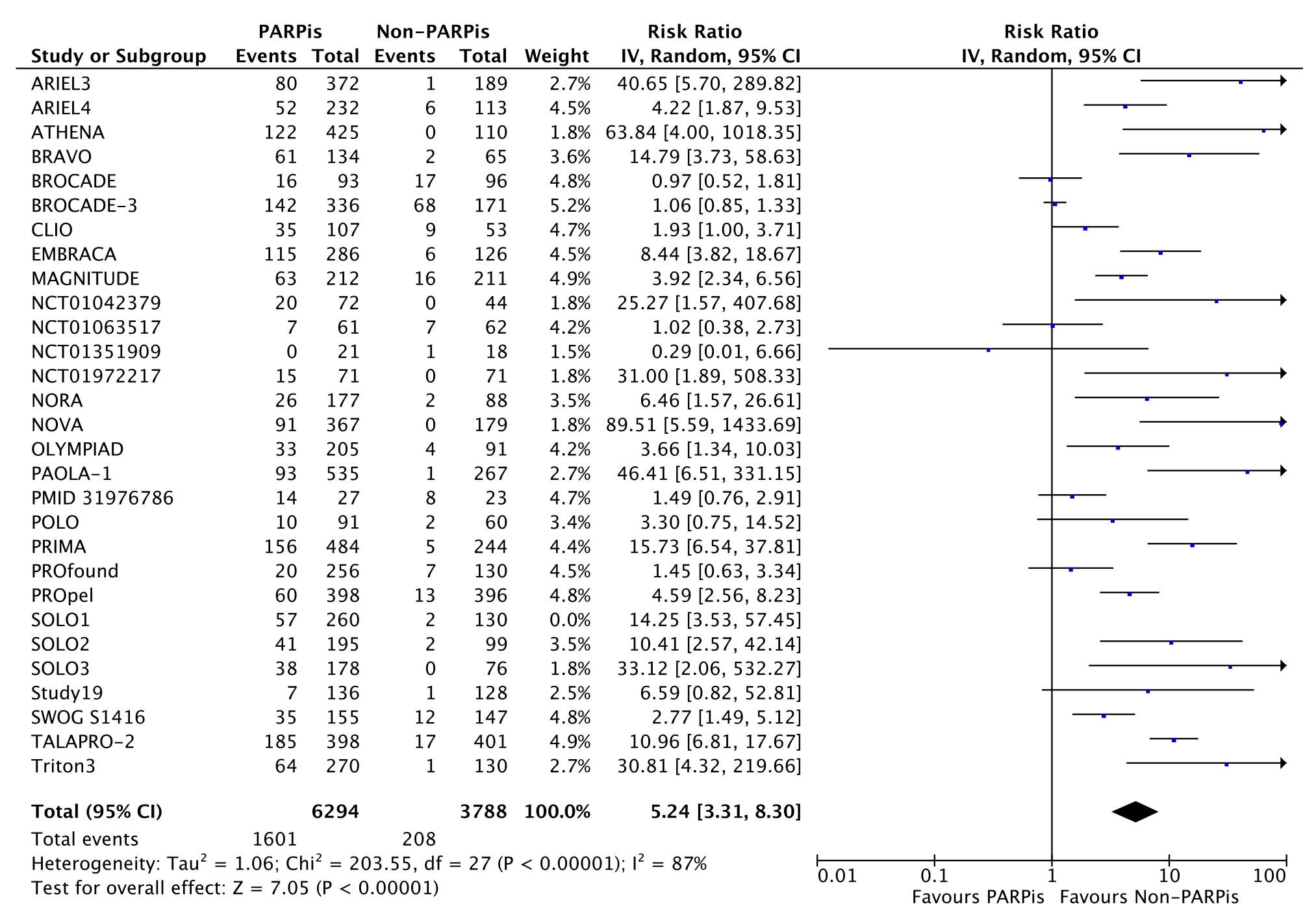 | 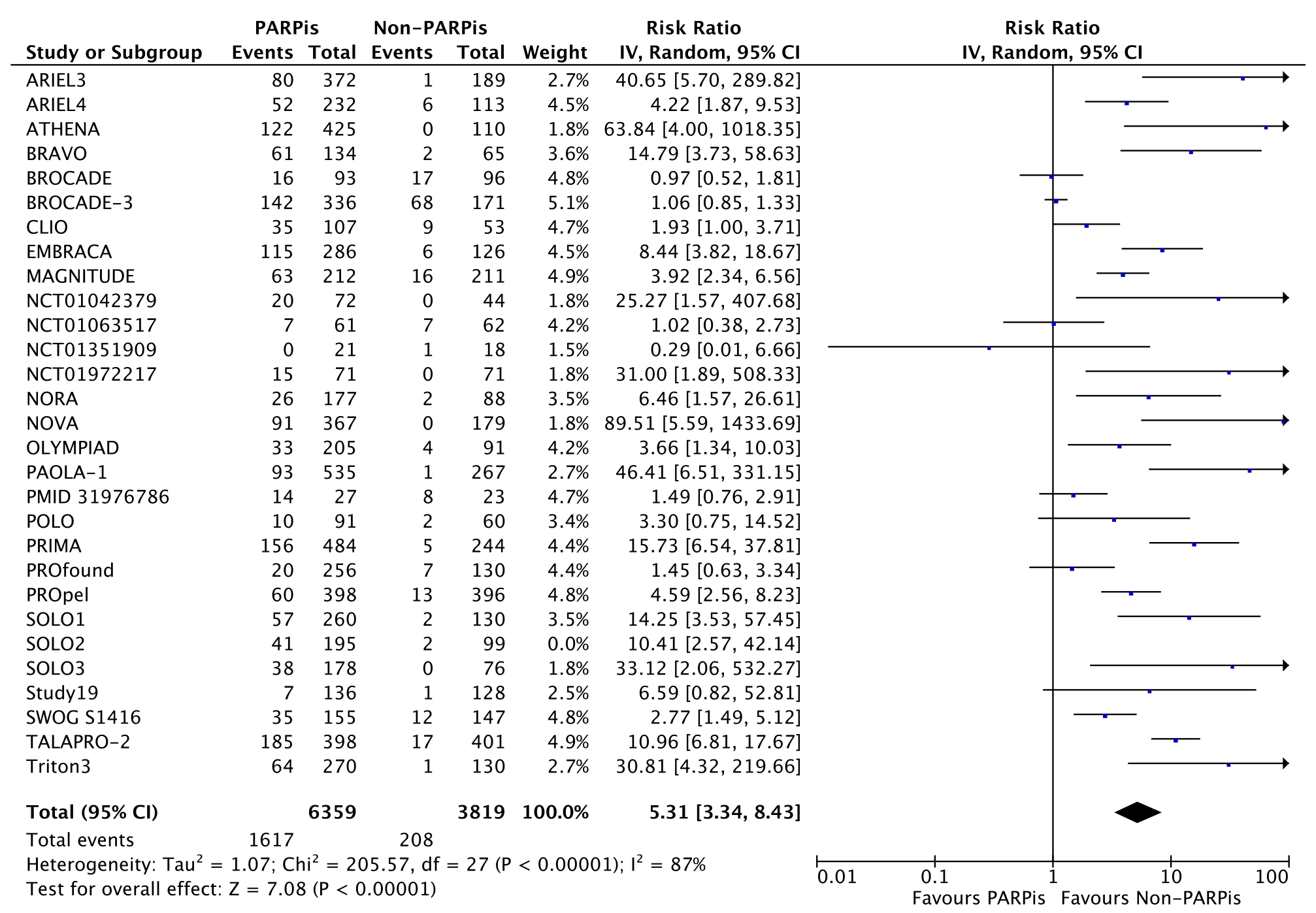 |
| 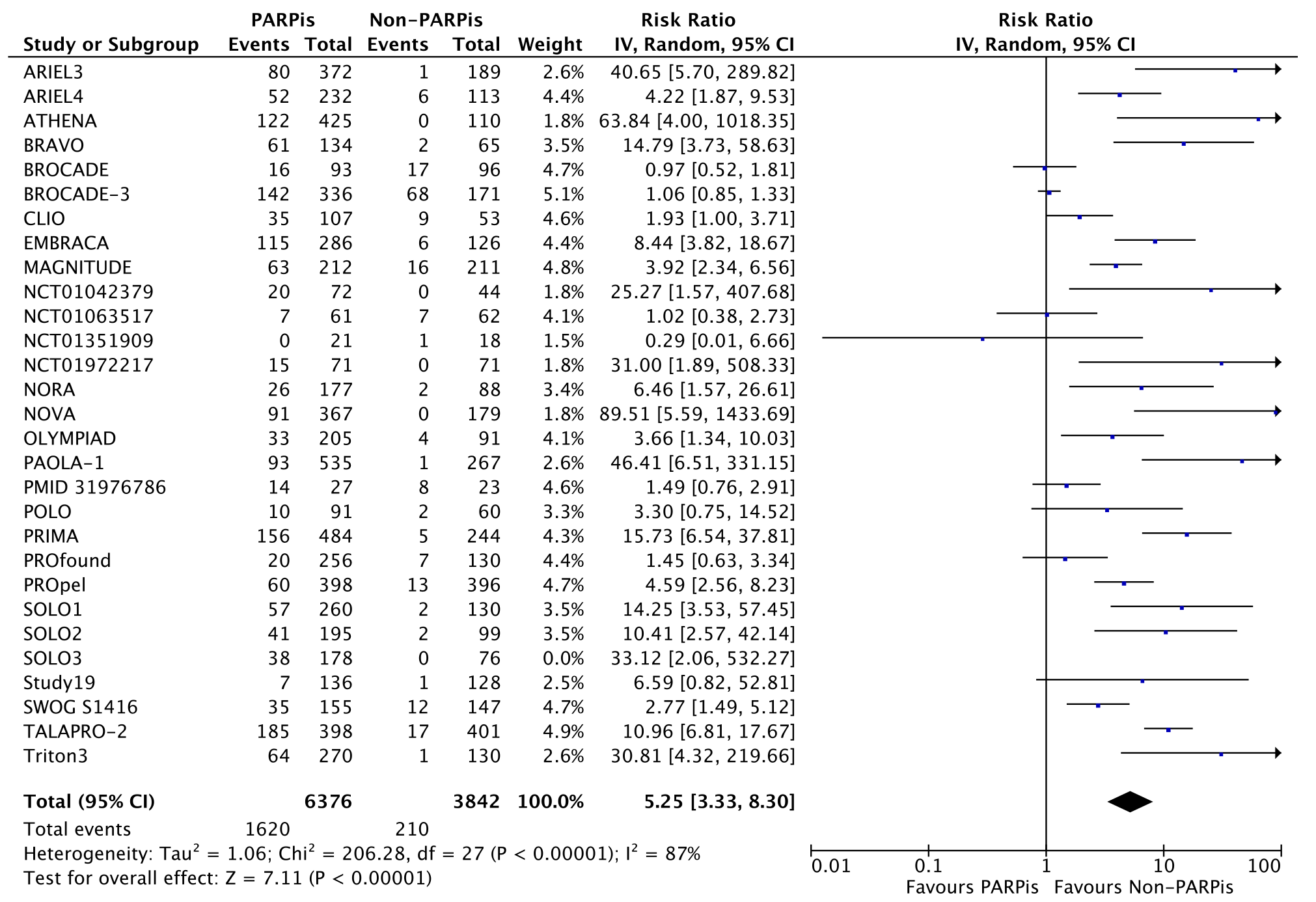 |  |
|  |  |
|  |  |

E

|  |  |
| --- | --- |
|  |  |
|  |  |
|  |  |
|  |  |
|  |  |
|  |  |
|  |  |
|  |  |
|  |  |
|  |  |
|  |  |

F

|  |  |
| --- | --- |
|  |  |
|  |  |
|  |  |
|  |  |
|  |  |
|  |  |
|  |  |
|  |  |
|  |  |
|  |  |
